# The Role of Near-Infrared Spectroscopy in Food Quality Assurance: A Review of the Past Two Decades

**DOI:** 10.3390/foods13213501

**Published:** 2024-10-31

**Authors:** Marietta Fodor, Anna Matkovits, Eszter Luca Benes, Zsuzsa Jókai

**Affiliations:** Department of Food and Analytical Chemistry, Institute of Food Science and Technology, Hungarian University of Agriculture and Life Sciences, 1118 Budapest, Hungary; matkovits.anna@phd.uni-mate.hu (A.M.); benes.eszter.luca@uni-mate.hu (E.L.B.); jokaine.szatura.zsuzsanna@uni-mate.hu (Z.J.)

**Keywords:** NIR, meat, meat product, milk, dairy product, honey, vegetable, fruit, tea, coffee, chocolate

## Abstract

During food quality control, NIR technology enables the rapid and non-destructive determination of the typical quality characteristics of food categories, their origin, and the detection of potential counterfeits. Over the past 20 years, the NIR results for a variety of food groups—including meat and meat products, milk and milk products, baked goods, pasta, honey, vegetables, fruits, and luxury items like coffee, tea, and chocolate—have been compiled. This review aims to give a broad overview of the NIRS processes that have been used thus far to assist researchers employing non-destructive techniques in comparing their findings with earlier data and determining new research directions.

## 1. Introduction

Preserving and monitoring food quality is an increasingly important part of a healthy diet. In addition, the issue of climate change is becoming more and more prominent. As a result of climate change, the stability of global food systems, food security, and diet quality are decreasing. Climate change affects, among other things, changes in soil fertility and yield, the composition of food, the bioavailability of nutrients, and resistance to pests [1]. Many chemicals are used to determine the most basic qualitative characteristics of our food—dry matter, protein, fat, carbohydrates, fibre, etc. The protein content is usually determined by conducting the Kjeldahl destruction process, which is a destruction process with concentrated sulfuric acid at a high temperature (380 °C) [2]. The fat content is determined by using a large amount of organic solvents (petroleum ether, hexane, chloroform, etc.) [3]. 

The residues of these techniques have a harmful effect on the environment. Although effective, these traditional analytical techniques require energy and are time-consuming.

To overcome these problems, a non-destructive and environmentally friendly chemical analytical method, near-infrared spectroscopy, offers the perfect solution. This is a secondary analytical technique which is based on mathematical relationships between the reference data and spectral results obtained by chemometric methods.

**Figure 1:**
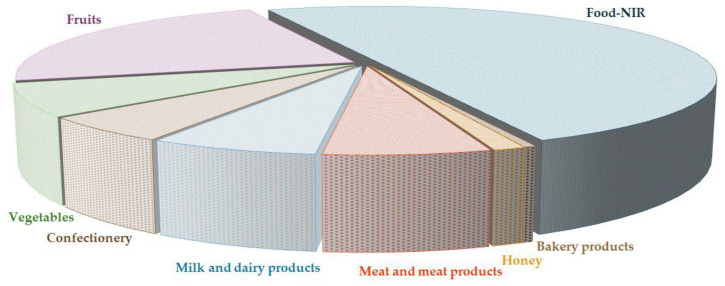
Publications on the topic from 2005 to 2024 (based on Scopus).

The technical advances in NIR instruments and the proliferation of chemometric computer software have made the technique one of the most used methods in the analytical toolbox. This is confirmed by the number of papers on the subject published over the past 20 years (Figure 1).

In this review work, the focus is exclusively on NIR spectroscopy techniques (NIRS). Other imaging techniques, such as hyperspectral or mid-infrared spectroscopy, are not discussed in this paper.

The basic principles of NIRS and the explanation of different chemometric methods are only partially described in this manuscript, given the vast literature available on these two topics. For a more detailed overview, attention is drawn to some previous summary works [4,5,6].

A rapid analysis and, after knowing the results, a quick intervention—such as those which goes into technical processes—are crucial during food quality control.

Conventional analytical techniques are unable to accomplish this. A protracted sample preparation and a measurement phase are features of both traditional and instrumental techniques. Traditional methods necessitate the operation of quality control laboratories, which call for skilled workers.

On the other hand, the NIR method can be applied offline, online, at-line, and in-line. In addition to not requiring the use of chemicals or sample preparation, it also operates without the need for skilled labour, which is crucial. When NIR sensors are positioned correctly in the technological process, we may quickly learn about the sample’s usual characteristics. 

The non-destructive technology uses a lot less energy than conventional analytical methods. 

The NIR method is not an absolute method, as its measurement accuracy depends on the accuracy of the reference method used.

Nevertheless, it can be stated that this fast, non-destructive technique plays an increasing role in the quantitative determination of key parameters of foods. Chemometric methods, which are developing more and more, offer the possibility to identify the origin based on the spectra, to determine the maturity status, and to detect possible adulteration.

## 2. Basics of NIR Spectroscopy

The electromagnetic radiation range of 12,500–3800 cm^−1^ (800–2500 nm) is the near-infrared radiation (NIR) region. 

**Figure 2:**
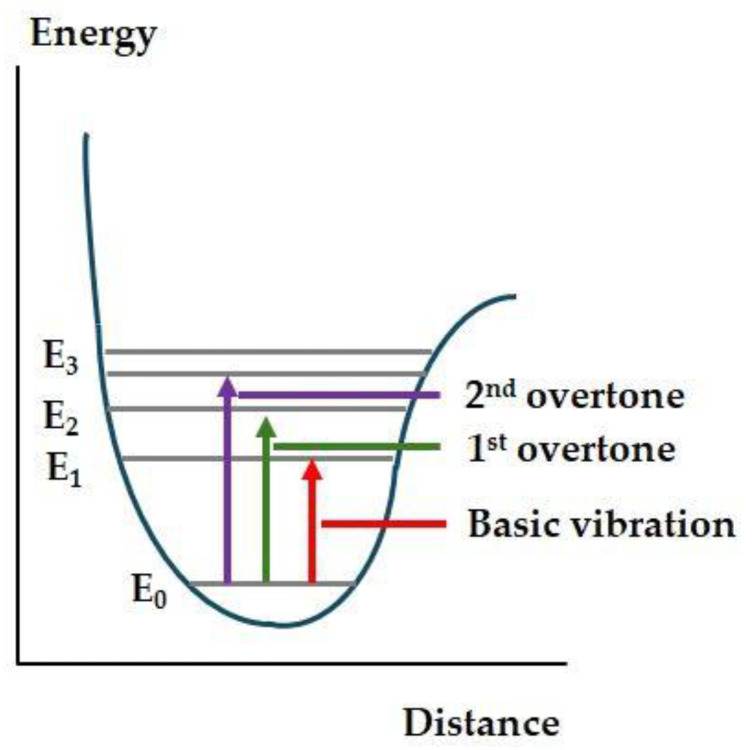
Excitation of vibration modes.

The energy in this range is no longer high enough to excite electron transitions, so only rotational and vibrational transitions can be detected. However, its energy is too high to detect these stretching and deformation vibrations (normal vibrations) clearly, so combinations and overtones of these appear in the NIR spectrum (Figure 2).

Infra-active molecules and molecular groups can be studied in this range, which change their dipole moment in response to electromagnetic radiation.

**Figure 3:**
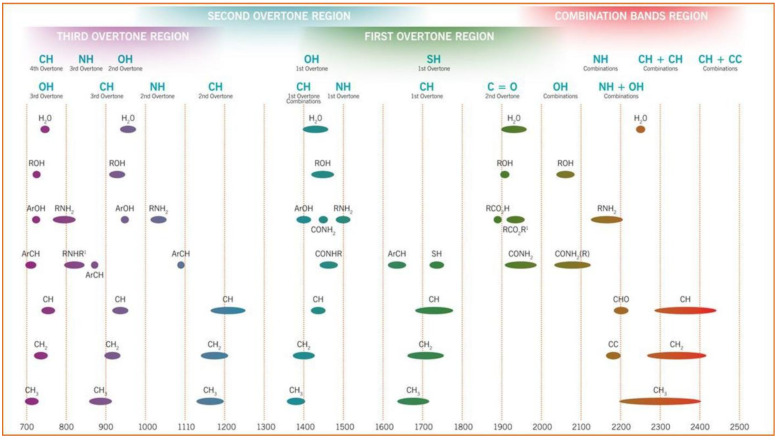
NIR band assignment [7].

The recorded NIR spectrum consists of overtones and combination vibrations of molecules that contain CH, NH or OH groups (Figure 3).

Therefore, NIR spectroscopy is suitable for the analysis of organic substances in food, agriculture, feed, chemical, and pharmaceutical products.

**Figure 4:**
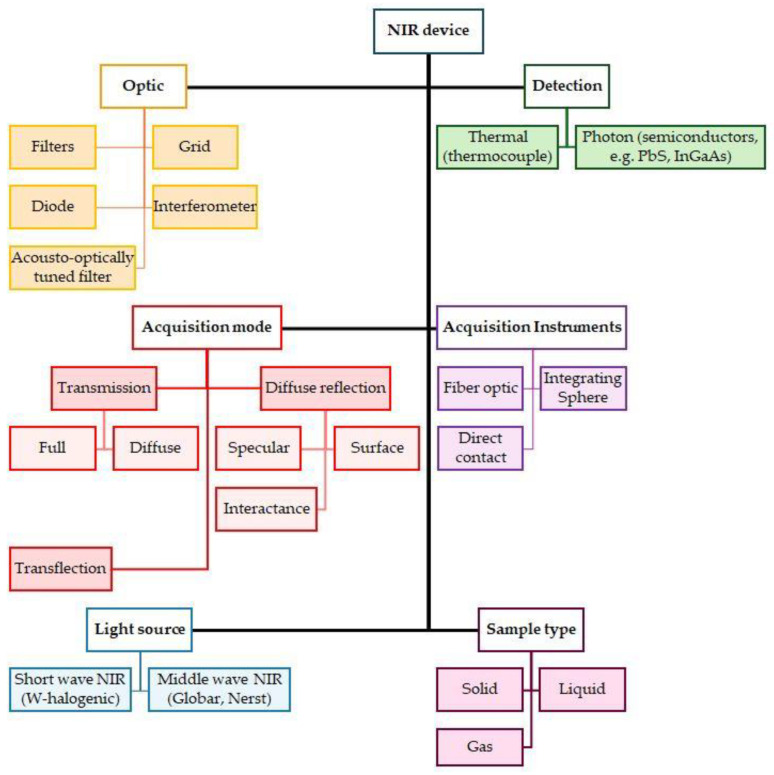
An overview of the NIR technique.

Figure 4 provides an overview of the NIR technique, including its optics, detection methods, spectrum recording options, light source, and sample type. 

**Figure 5:**
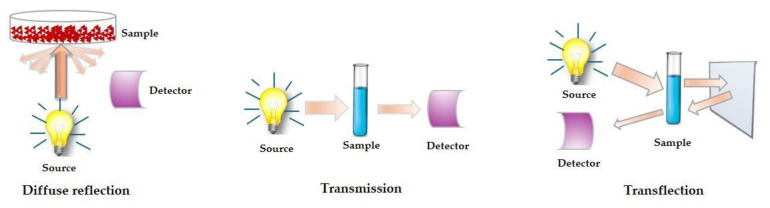
Measurement possibilities in NIR spectroscopy.

Focus should be placed on the spectra’s acquisition method (Figure 5).

Solid samples can be examined using the diffuse reflection method (PbS detector). Since the photon penetrates only a few millimetres deep into the sample in this instance, the layer thickness of the sample has no effect on the spectrum image. Although, in this instance, the particle dispersion needs to be carefully considered. A detrimental scattering phenomenon may result from an excessively diverse particle dispersion.

The transmission technique can be applied to liquids (InGaS detector) or to colloidal samples (Si diode). The homogeneity of the samples is crucial when dealing with liquids. Otherwise, harmful scattering phenomena may occur. Depending on the sample, the ideal layer thickness (optical path length) can be between 0.5 and 2 mm.

When examining colloidal samples, signal loss may occur due to inadequate layer thickness. If the layer thickness is too big, the infrared photon is absorbed and does not pass through the sample, while if the layer thickness is too small, the signal of the sample is detected, and, accordingly, we obtain a spectrum that is too noisy.

In food analyses, colloidal samples with questionable homogeneity are common. To provide an “average″ image, the spectra are obtained in this instance while the samples are rotating.

An insufficient spectrum is a common issue that arises when the transmission process is recorded. The transflection treatment can be conducted to get rid of this. It combines diffuse reflection and transmission. When measuring “problematic″ colloids, it is preferred.

A special technique is the attenuated total reflectance (ATR) phenomenon, which is also known to be utilized in the NIR range but is typically used in the mid-infrared (MIR) range.

It may be appropriate to obtain a summary of the most up-to-date infrared detection possibilities from Saleem et al.’s [8] summary study.

Infrared detectors that are currently in use are based on traditional inorganic semiconductors like Si, Ge, and InGaAs. 

The need for cutting-edge imaging technologies is growing in other industrial applications, including virtual reality, driverless cars, and healthcare. Consequently, processed semiconductor photodetectors have already surfaced, allowing for the creation of numerous excitations and a tunable spectrum response. 

Current studies deal with solution-processed infrared detectors and imaging devices based on colloidal quantum dots, perovskites, organic compounds and 2D materials.

Mobile near-infrared sensing is becoming an increasingly important method in many research and industrial fields. Jiang et al. provides a detailed overview of mobile near-infrared sensing prototypes, data ignition techniques, machine learning methods, and relevant application areas [9].

## 3. NIR Data Evaluation, Chemometric Methods

Evaluating the NIR spectrum is challenging because combinations and overtones of the chemical and deformation vibrations of the infrared bonding groups appear in the spectra, so the peaks cannot be assigned to a specific compound. 

The first step in the evaluation is to apply various data pre-processing techniques, such as “cleaning″ the spectra from various noises, separating overlapping peaks, etc.

A multiplicative scatter correction (MSC) is the most used scatter correction method that removes both additive and multiplicative effects in diffuse reflectance spectroscopy [10,11]. MSC is a model-based method in which all spectra are corrected by the average spectrum for the dataset. It works primarily in cases where spectral variations are due to scattering. A widely used variance correction method is standard normal variate (SNV) [11,12]. This method centres the spectral data, line by line (sample by sample), correcting for baseline shifts and then scales. This reduces variations due to differences in optical path length. Baseline deviations can also be corrected by straight line subtraction (SLS), where the algorithm fits a straight line to the spectrum and then subtracts these values from the original spectrum. Various other derivation or smoothing methods, such as the Savitzky–Golay algorithm [13], can also be used. Derivation methods are used both to improve the resolution and to correct the baseline for NIR spectra. By resolving overlapping absorption bands, the accuracy of the quantitative estimate can also be improved. For FT-NIR spectroscopy, the first derivative (FD) and the second derivative (SD) spectra are the most used ones, but it should be noted that the noise increases with the derivative. In addition to the individual data processing methods, a combination of them can improve the performance of mathematical models, e.g., FD + SNV, and SD + SNV.

Various chemometric techniques are used for qualitative or quantitative assessment, such as the principal component analysis (PCA), polar qualification system (PQS) [14], cluster analysis (CA), and partial least squares regression (PLSR). 

The NIR spectroscopy is most used for the quantitative estimation of various constituents based on a calibration model built from reference data and spectral data. Different, essentially linear, regression methods can be used for this purpose, given that NIR spectroscopy measurements are usually based on the Lambert-Beer law, which assumes a linear relationship between absorbance and concentration. The most used linear algorithms are: PLSR, PCR (principal component regression), and MLR (multiple linear regression).

Since the number of explanatory variables (spectral data) is significantly larger than the sample size, traditional linear regression methods are not applicable, and PLSR has become most widespread [15].

**Figure 6:**
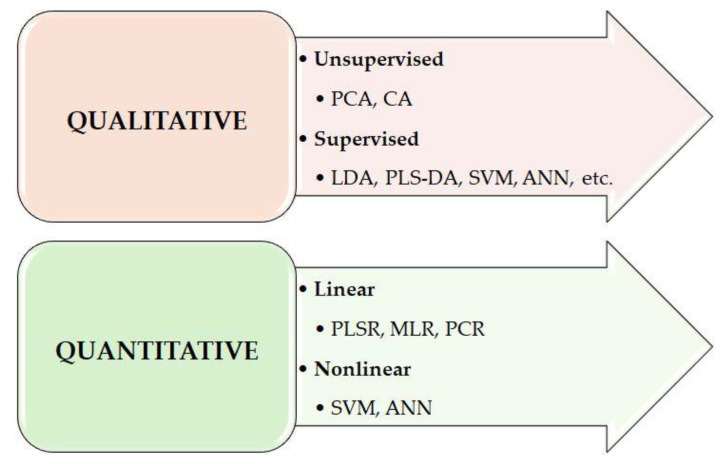
Multivariate data analysis methods.

The analysis of quality attributes (e.g., origin, type of product, identification of origin, adulteration, type of plant, etc.) is usually performed using classification methods, allowing the samples to be classified into classes. Non-linear models [16], such as artificial neural networks (ANNs), AdaBoost, local algorithm (LA) or support vector machines (SVMs), are commonly used to solve classification problems (Figure 6).

The classification model’s performance was assessed using standard metrics such as sensitivity, specificity, precision, and accuracy. These metrics were calculated from the counts of true positives (T_P_), true negatives (T_N_), false positives (F_P_), and false negatives (F_N_), employing the Equations (1)–(4) [17,18]:

(1) $$Sensitivity=\frac{T_{P}}{T_{p}+F_{N}}$$

(2) $$Specificity=\frac{T_{N}}{T_{N}+F_{P}}$$

(3) $$Precision=\frac{T_{P}}{T_{p}+F_{P}}$$

(4) $$Accuracy=\frac{T_{P}+T_{N}}{T_{P}+T_{N}+F_{P}+F_{N}}$$

**Figure 7:**
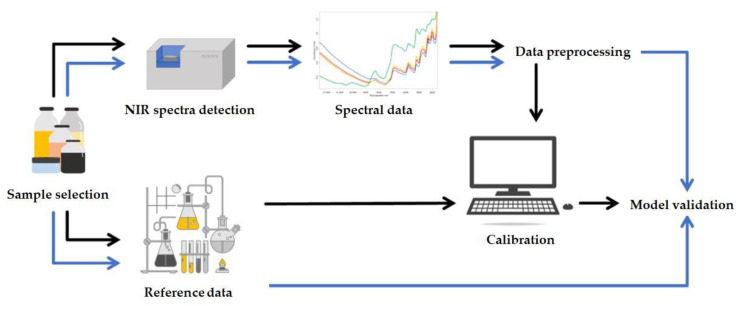
Main steps of model building [19]. 🢣 calibration; 🢣 validation.

Since each material has different spectral properties (fingerprint-like pattern), a separate model must be developed for each sample matrices. The data can be analyzed using many different methods, but the main steps of model building are the same (Figure 7): sample selection, spectral recording, reference data determination, data pre-processing, calibration, and model validation.

Among the multivariate regression procedures, parameters indicating the performance of the most commonly used PLS regression procedure are summarized in Table 1.

The root mean squared error (RMSECV for cross-validation; RMSEP for test-validation) is calculated according to the following relation:

(5) $$RMSECV\;or\;RMSEP=\sqrt{\frac{1}{N}\sum_{i=1}^{N}{\left(y_{i}-{\hat{y}}_{i}\right)}^{2}}$$

RMSECV or RMSEP: root mean square error of cross-validation or test validation (the unit of measurement is the same as that of the estimated parameter) 

y_i_: measured (reference) value of the i-th component

${\hat{y}}_{i}$: estimated value of the i-th component

N: number of samples tested

The minimum–maximum number of main components of PLS is not regulated, it basically depends on the number of samples. In most cases, the minimum value is set at three and the maximum value is set at ten to avoid under- or over-fitting.

Ratio of Performance to Deviation (RPD) is calculated according to the following relation [20]:

(6) $$RPD=\frac{S_{d}}{SEP}$$

where S_d_ is the standard deviation of the samples

(7) $$S_{d}=\sqrt{\frac{1}{N-1}\sum_{i=1}^{N}{\left(y_{i}-\bar{y}\right)}^{2}}$$

$\bar{y}$: the average of the measured (reference) values

SEP is defined as the standard error of prediction:

(8) $$SEP=\sqrt{\sum_{i=1}^{N}\frac{{\left({\hat{y}}_{i}-y_{i}-bias\right)}^{2}}{N-1}}$$

(9) $$bias=\sum_{i=1}^{N}\frac{\left({\hat{y}}_{i}-y_{i}\right)}{N}$$

NIRS is a fast and efficient analytical tool in the food industry. As an advanced chemometric tool, multipath analysis has great potential for solving a wide range of food problems and analyzing complex spectroscopic data. The development, advantages, and limitations of the multipath models used to analyze NIRS data and the various multipath models are summarized in Yu et al. [21].

## 4. Limitations of NIR Spectroscopy

The limits of NIRS include its low sensitivity due to low absorption coefficients, which causes the detection limit to be higher. NIRS is an indirect method that requires the development of a multivariate calibration model against a suitable reference method. Therefore, the accuracy of the NIR data depends on the precision of the reference measurements and shouldn’t be higher than that. However, the accuracy detection of reference data does not clearly mean that the parameter examined can be determined by NIR spectroscopy. The technique has a concentration limit. The parameter being examined, the matrix’s complexity, the reference’s sensitivity, and the NIR technology being employed all affect this limit. The detection limits for more complicated matrices (like food samples) are roughly 1000 mg/L (0.1%). For less complex matrices (e.g., milk, energy drink), this detection limit can also reach 50–100 mg/kg (ppm) [22].

In NIR spectra, the absorption bands come from combinations of overtones and/or normal vibration movements. They are wider and much less intense than basic absorption bands. Various data management procedures can reduce the signs caused by noise and separate overlapping peaks.

Temperature variations play a crucial role in developing predictive models with NIRS. They alter the location and intensity of the NIR spectral absorption bands, impacting the calibration models’ predictive accuracy. This issue can be addressed by employing local and global temperature compensation techniques. Local models tend to be vulnerable to temperature shifts, whereas a global model, which utilizes sample spectra across the full temperature spectrum, demonstrates robust predictive performance [23]. 

Measuring the moisture content of samples is a common task in food analysis. However, the moisture content in samples can pose challenges, particularly when assessing their protein and sugar content. For solid samples, methods like lyophilization or drying are suitable for addressing this issue. For liquid or colloidal samples, it is advisable to use a transflection spectrum rather than the conventional transmission spectrum [24]. 

NIR spectroscopy requires no or minimal sample preparation. This primarily means homogenization of fluid and colloid patterns. Diffuse reflection (DRIFTS, Diffuse Reflectance Infrared Fourier Transform Spectroscopy) is used to analyze powders and other solid matters. The collection optics in the DRIFTS accessory are designed to exclude spectral reflected radiation and collect the diffuse reflected light as much as possible [25].

About the challenges of nearly infrared spectroscopic measurements, Hong et al. published a detailed review [26].

## 5. Applications of NIRS for Quality Assurance

### 5.1. Bakery Products, Pastas, Biscuits, and Snacks

The application of near-infrared (NIR) technology is not yet common in the baking industry, unlike in the milling sector, where NIR technology is used to monitor raw materials, processes, and products [27]. 

#### 5.1.1. Bakery Products

Previous articles have mainly focused on nutritional analyses of bakery products, so the results are mainly related to the determination of protein, fat, and sugar content. 

Scientific literature primarily focuses on the nutritional analysis of baked goods made from various flours, such as wheat, rice, buckwheat, and corn. The analyses typically estimate the content of protein, fats, sugars, dietary fibre, ash, monounsaturated fatty acids (MUFAs), polyunsaturated fatty acids (PUFAs), and sodium. The total carbohydrate and energy content can be derived from NIR data [28,29,30].

Reference data from gas chromatography-flame ionization detection (GC-FID) are used for the NIR method to determine the ethanol content in packaged whole-grain bread [31].

In the baking industry, controlling the fermentation state of bread is crucial. The inline application of technology based on PLS-DA evaluation of NIR spectra offers a way to monitor the fermentation state during the production process, allowing for the filtering of potential defects before baking [32].

Edible coatings, such as those with probiotic, antimicrobial, or antioxidant properties, can be utilized to prolong the shelf life of products. The drying of the coating is a critical phase in this process. The spectra obtained from monitoring the drying process provide a detailed description, enabling the clear differentiation of various coatings and drying durations [33].

Two-dimensional correlation spectroscopy (2D-COS) was utilized to explore the processes of deterioration. The key structural factors in bread rancidity include the crystallization of amylopectin within the starch and the loss of water content through evaporation and diffusion from the core to the crust. Two-dimensional-COS enabled the distinction of the detailed sequence of structural events over the investigated time intervals: crystallization of amylopectin, evaporation of weakly and strongly hydrogen-bonded water, and reorganization of starch’s OH functions [34]. 

NIR and the electronic nose provide an ideal solution for assessing the volatility and texture of the dough, thereby testing the quality of sourdough bread [35].

The adulteration of fats also presents a challenge in the baking industry. A 1:1 adulteration model was created using commercially available margarine and butter samples. The act of adulteration was confirmed by PCA of Raman and NIR spectra, proving successful not only in the fat examination but also in the analysis of baked goods produced with them [36].

Foreign food contaminants, such as metallic iron, polypropylene plastic, and hair fibres, were detected in bread samples using NIR and computer vision (CV). The evaluations achieved an accuracy of over 92% using a discriminant analysis paired with Savitzky–Golay smoothing [37].

Table 2 presents a summary of the data pre-processing and chemometric methods employed in the research.

#### 5.1.2. Pastas, Biscuits, and Snacks

Although dry pasta is traditionally not considered to be a bakery product, it does fit neatly into any other food category, hence it is discussed here.

Following extrusion, the dough’s optimal moisture content was achieved through controlled drying, with the process monitored by NIR reflectance spectroscopy [38,39]. 

The NIR technique was also used to determine the nutritional value (energy, protein, fat, carbohydrate, sugar, and fibre) of dry pasta. A PLS regression was used in data processing to determine the correlation between reference and spectral data [40]. Nutritional analyses were performed by Cayuela-Sánchez et al. [41], and in addition to those already mentioned, the parameters studied were extended to determine of saturated fatty acids (SFA), monounsaturated fatty acids (MUFAs), and polyunsaturated fatty acids (PUFAs). Spectra were recorded from both intact and ground samples, and reference data were also determined for both conditions. 

For egg-based dry pasta, egg content is an important qualifying parameter, and its determination is therefore a key issue.

Traditional methods often recommend spectrophotometry, specifically the Lieberman-Burchard reaction. Chromatographic techniques like gas chromatography with flame ionization detection (GC-FID) or mass spectrometry (GC-MS), and high-performance liquid chromatography (HPLC) are also prevalent in food analysis. The Lieberman-Burchard reaction has a drawback: it measures sterol concentration without distinguishing cholesterol, which can be problematic for pasta with minimal egg content, such as two eggs, where the flour’s phytosterol content may significantly alter the results. Additionally, this method is a time-reaction, and its reproducibility is debatable. Chromatographic methods require extensive sample preparation, making them impractical for routine dry pasta testing. Addressing this issue, Fodor et al. [42] introduced a NIR method based on calculations. By considering the fat content of pasta ingredients like wheat and durum flour, and lyophilized eggs, they calculated reference values through a theoretical model and then achieved a successful correlation using a PLS regression. Bevilacqua et al. [43] utilized their samples with a known egg content and observed that the spectral profile was affected by the production process, especially the drying temperature and duration. They employed a multivariate data analysis technique (ASCA), which is based on the ANOVA concept, in conjunction with locally weighted PLS regression (LWR-PLS). This non-linear approach yielded a stronger correlation than the conventional PLS regression. Adulteration poses a problem in the case of pasta products as well. The most frequent form of fraud is the substitution of pure durum flour with a mix of durum and wheat flours. To detect this fraud, De Girolamo et al. [44,45,46] effectively used the FT-NIR method alongside various chemometric techniques, such as PLS-DA and LDA. The duration of heat treatment, and temperature of fresh unfilled egg pasta (tagliatelle, fettuccine, and tagliolini) were examined. The experiment demonstrated that an NIR analysis can be effectively used for the rapid monitoring of thermal processing parameters [47].

Xanthine (caffeine, theobromine, and theophylline) and polyphenols (catechins and epicatechins) are primarily responsible for the bitter taste of baked goods containing coffee, cocoa or chocolate. For the Fourier transform near-infrared (FT-NIR) spectroscopic method, the reference measurements were performed using liquid chromatography LC-ESI/mass spectrometry MS-MS method. This method can be directly applied to solid products and may extend to other flavour molecular markers like sugars, potentially for routine monitoring of standardized bitter taste quality in actual industrial production [48,49].

In assessing the physicochemical characteristics of fresh egg pasta made by extrusion and lamination, it became evident that these two techniques yield pasta with distinct properties, particularly in colour and starch gelatinization. Although, no notable difference was observed in water absorption during cooking. FT-NIR spectral classification procedures effectively differentiated between the two types of pasta [50].

The physicochemical attributes of fresh pasta, such as water activity, colour, water absorption index, and hardness, are crucial determinants of its stability, quality, and consumer appeal. FT-NIR analysis tracked the structural changes in dough stored under various temperatures and durations. These changes, linked to the interactions between water, starch, and proteins, were significantly influenced by storage temperature, impacting the dough’s physicochemical properties, like hardness [51].

In biscuit production, kneading and rolling are vital. The NIR technique, paired with the novel soft multiclass compatible classification method (PLS2-CM), effectively pinpointed defective products during these stages. During kneading, the method could distinguish well-kneaded dough from defective ones.

Although a reliable classification model for determining excess water was not achieved, the same doughs were modelled after fermentation and during rolling with complete sensitivity and precision (100%). This indicates that the physicochemical changes that occur during fermentation are critical in determining the absence of defects in kneaded biscuit doughs using NIR spectroscopy [52]. 

Foreign food contaminants, such as metallic iron, polypropylene plastic, and hair fibres, were detected in bread samples using NIR and computer vision (CV). The evaluations achieved an accuracy of over 92% using discriminant analysis paired with Savitzky–Golay smoothing [53]. The research results related to meat and meat products are summarized in Table 2.

The concept of snacks is rather complex, as it refers to sweet and salty snacks that are not eaten as a main meal. In the case of salty snacks, in addition to the fat and salt content of the macro components [54,55,56,57], an important issue is the determination of the acrylamide content [57,58], which is highly dangerous from a physiological point of view.

Several classification models have been developed for the technological process, the raw materials and the country of origin of the finished product [59].

**Table 2:** NIR test results for bakery products, pastries, dough, biscuits, cake, snacks.

Sample	Investigated Parameter	Concentration Range	Chemometrics Data			References
			Pre-Treatment, Regression	R^2^	Root Mean Square Error	
Bread	Moisture, %	49.05–53.85	MLR	0.92	0.46	[30]
			PCR	0.85	0.61	
			PLS	0.88	0.55	
		n.i.	PLS	top 0.963 bottom 0.937	2.49; 2.87 3.08; 3.15	[33]
	Protein, %	5.3–11.7	SNV, DT, 1st der. PLS; MH > 3.5	0.99	0.14; 0.17	[28]
		10.8–16.2	1st der. PLS; MH > 3.0	0.989	0.16	[29]
		1.93–8.89	MLR	0.99	0.29	[30]
			PCR	0.97	0.46	
			PLS	0.99	0.29	
	Fat, %	1.2–13.5	SNV, DT, 1st der. PLS; MH > 3.5	0.99	0.27; 0.33	[28]
		1.2–31.1	SNV, PLS; MH > 3.0	0.99	0.79	[29]
	Dietary fibre, %	2.8–9.4	SNV, DT, 1st der., PLS; MH > 3.5	0.89	0.60; 0.55	[28]
	Sugar, %	2.1–8.5		0.96	0.43; 0.54	
		0.9–10.9	MSC, PLS; MH > 3.0	0.988	0.28	[29]
	Ash, %	1.1–2.6	SNV, DT, 1st der., PLS; MH > 3.5	0.91	0.1; 0.15	[28]
	SFA, %	0.1–3.0		0.90	0.15; 0.16	
	MUFA, %	0.2–2.9		0.91	0.23; 0.25	
	PUFA, %	0.22–6.1		0.92	0.22; 0.31	
	Total carbohydrate, %	28.7–51.8		0.98	1.1; 1.17	
	Energy; kJ/100 g	738–1421		0.99	21; 19	
	Total carbohydrate	-	Calculated from NIR predicted data	n.i.	0.75; 0.91	
	Energy; kJ/100 g	-		n.i.	14; 14	
	Ethanol, %	0.0–3.45	MSC, DA, MLR	classification 100%		[31]
	Fermentation point		PLS-DA, ROC	sensitivity 86–88%		[32]
	Staling	n.i.	EMSC, MCR-ALS	explained variance 99.9994, sum of squared residuals 0.75776		[34]
	Contaminants		SGS, PCA, DA	accuracy: 92–95%		[37]
Pastry	Moisture	31.4–74.4	MSC, 1st der., PLS	0.956	2.4	[38]
		7.37–31.42	PLS	0.994	3.32; 3.41	[39]
	Egg content (pieces)	0.5–9.1 pieces	MSC, 1st der., PLS	0.907	0.6; 0.7	[42]
		n.i.	ASCA, LWR-PLS	n.i.	1.01; 1.25	[43]
	Authentication	durum wheat, mix wheat	PCA, PC-LDA, SVMc, PLS-DA	sensitivity 95%, sensitivity 95%, specificity and accuracy 94%		[44,45,46]
	Thermal treatments	1.78–3.31	2nd der., PLS	0.781	0.183	[47]
	Extrusion or lamination	-	PCA	Accuracy 100%		[50]
	Storage time, days, temp., °C	Time: 0–75 d Temp. 0; 5; 10	PLS	0.968 (0 °C) 0.974 (5 °C) 0.968 (10 °C)	4.5 (0 °C)) 4.1 (5 °C) 4.4 (10 °C)	[51]
Pastry doughs	Kneading or rolling		SNV, 1st der., PLS2-CM	sensitivity and specificity 100%		[52]
Biscuits, cake	Protein, %	5.3–12.2	RS, OLS, PLS, DA, kNN, NB	0.941	0.385	[53]
	Lipid, %	0.8–25.0	MSC, OLS, PLS, DA, kNN, NB, PLS-DA, PLS-kNN, PLS-NB	0.992	0.56	
	Fatty acid, %	0.2–17.0	RS, OLS, PLS, DA, kNN, NB	0.988	0.39	
	Carbohydrate, %	42.7–87.0		0.965	1.46	
	Fibre, %	0–21.6		0.906	0.72	
	Energy, kJ/100 g	1544–2135		0.986	25.1	
	Salt, %	0–2.8	SNV	0.9	0.182	
	Main cereals	five kinds	PLS-kNN	classification 100%		[36]
	Cooke type	14 kinds	PLS-kNN	classification 100%		
	Adulteration-fat	n.i.	SVD, PCA	classification 100%		
	Xanthines, mg/kg	1–1600	1st der., PLS	0.96	<10%	[48,49]
	Polyphenols, mg/kg	0–83		0.96	<10%	
	Bitter taste	<4–8	PAA	n.i.	n.i.	[48]
Snack	Cereal base and sucrose coated, %					[54]
	Sucrose	1.23–25.73	SGS, DT, PLS	0.97	1.47	
	Glucose	1.04–5.06	SGS, DT, PLS	0.95	0.36	
	Fructose	1.53–3.86	SGS, DT, PLS	0.59	0.2	
	fat, %	2.2–45.1	SNV, PLS	0.98	1.1	[55]
	carbohydrates, %	45.1–69.7	SNV, 1st der., PLS	0.92	1.9	
	sugar, %	1.7–8.6 g/100	1st der., PLS	0.93	0.47	
	protein, %	3.0–40.1	MSC, 1st der., PLS	0.98	0.93	
	salt, %	0.7–2.5 g/100	SNV, 1st der., PLS	0.91	0.16	
	energy, kJ/kg	1264.3-	SNV, PLS	0.87	92.03	
	Classification	Frying oil Raw material Production technology Origin	PRPropMLP	Accuracy 83% 98% 91% 90%		[59]
Chips—potato	Fat, %	1.2–4	MSC, PLS	0.98	1.21	[56]
		26.7–49.3	SNV, PLS	0.99	0.99	[57]
	Moisture, %	18–45	MSC, PLS	0.99	0.82	[56]
	Dry matter, %	82.9–98.6	SNV, PLS	0.97	0.84	[57]
	Acrylamide, mg/kg	40–1770	SNV, PLS	0.83	266	[57]
	μg/kg	56.7–789.7	CARS-PLS	0.71	61.1615	[58]

### 5.2. Meat and Meat Products

Meat is one of the most important foods because of its nutritional properties. It is mostly composed of water (~73%), amino acids (~23%), and fatty acids (~1.8%), and additionally it contains cholesterol, phospholipids, minerals, and vitamins [60,61]. 

As people’s standard of living continues to improve and the supply of meat on the market becomes more abundant, expectations for meat quality have also risen. Consumers are increasingly concerned not only with the nutritional value but also with the taste, texture, and appearance of meat, as well as factors like convenience, health, and safety [62]. The development of rapid, environmentally friendly, and non-invasive methods for predicting, certifying, and authenticating meat quality has become a priority in recent years.

In this context, near-infrared (NIR) techniques are most commonly used for meat analysis [63,64]. MSC and SVN are mostly used for data pre-processing, and it is equally important to test the derivatives, e.g., the 1st and the 2nd ones [65]. Furthermore, in data management, the first derivative is recommended for homogeneous products, while the second derivative is preferred for heterogeneous products, as it reduces scattering effects caused by variations in grain size [66].

As a result, the prompt identification of meat quality is essential not only in the laboratory but also in industrial settings, where it is used to monitor technological processes, reduce losses, and increase exports. A key example of this is the study conducted by Isaksson et al. [67], in which the quality parameters of minced meat—such as fat, water, and protein content—were determined under industrial conditions.

Building on this, in recent years, numerous studies have focused on the industrial testing of meat, with a growing emphasis on the utilization of specialized portable equipment to facilitate monitoring [60].

It is important to recognize that the actors in the food supply chain have different priorities and, therefore, different assessments of quality. Important factors include shape, flavour, freshness, and health safety. They pay attention to the appearance of meat, particularly colour and fat content, as these influence their perception of freshness and meat quality, although this may vary regionally.

Technological properties such as water retention, colour, and pH are important meat quality indicators that correlate with consumers’ sensory evaluation.

For example, a dry, dark, firm texture indicates DFD meat, while pale, soft, and flaky meat is referred to as PSE in the literature. The occurrence of these meat defects poses a significant issue for the meat industry due to their unappealing nature to customers and poor processing characteristics, such as lower yield and high spoilage potential, compared to normal meat [68].

#### 5.2.1. Beef Meat

A model was built by Tejerina et al. [69] for beef samples to predict some of the DFD meat parameters, such as colour (L*, a*, and b*), which offers a good opportunity for internal quality control in slaughterhouses. Samuel et al. (2011) [70] found that the Vis-NIR range was superior to the NIR range, as the Vis-NIR region of the spectrum contained abundant information about muscle pigments [71]. 

The moisture, fat, and protein content of bovine meat was determined by Dias et al. [72] using NIRS. 

In the case of beef, the quality of the meat was found to be largely dependent on intramuscular connective tissue (IMCT) components. The measurement of muscle and IMCT components were identified as important for quality determination and prediction. In a related study, Andueza et al. developed a NIRS method to predict IMCT components from fresh and lyophilized samples while investigating whether the accuracy of the model varies for meat from different body regions. The efficiency and reliability of the NIRS models were found to depend on the variability of reference values. Additionally, the meat was characterized by a high water content (75%), which can interfere with the absorption of other components in the NIR spectrum and, thus, affect the results of NIRS predictions [73].

Their study investigated Vis/NIRS models for FA prediction in fresh and lyophilized beef samples. No significant difference in performance between models for 16:0, 18:0, 18:1 n-9, 18:2 n-6, 20:4 n-6, 22:5 n-3, 22:6 n-3, saturated, monounsaturated FA, and total n-3 long chain PUFAs was found, but the standard error of total PUFAs, total n-3 PUFAs, total conjugated linoleic acid, 20:5 n-3, and 18:3 n-3, improved by an average of 21% in lyophilized samples [61]

Steer meat samples were examined, and a NIRS model was built to predict ether extract, among other parameters. An excellent result was obtained (R² = 0.92; RPD = 3.32), and it was found that ether extract and gross energy results are correlated, with better predictability of results achieved when MSC is applied to raw spectra. This improved predictability may be attributed to the difference in the refractive index of samples with varying ether extract contents [74].

#### 5.2.2. Pork Meat

The ability of NIRS to predict pork meat quality characteristics of early post-mortem samples was investigated, but it was found that no correlation was achieved with the PLS method. Although, promising results were obtained in predicting IMF (intramuscular fat) content [75].

Balage et al. [76] used NIR spectroscopy to predict meat pH, colour, IMF, and shear force (WBSF) to build classification models that can categorize meat based on tenderness and juiciness. They found that their PLSR- and Vis/NIRS-based models were inaccurate for IMF and WBSF, respectively, and needed further improvement. 

An NIRS method for fat characterization of live and slaughtered pigs was developed by Pérez-Marín et al. [77]. The spectra were collected in five analysis modes: live animal, carcass from slaughterhouse, subcutaneous fat sample, subcutaneous fat sample without skin, and transverse section. Calibrations were developed to predict the four main fatty acids (FA) (palmitic acid, stearic acid, oleic acid, and linoleic acid) in Iberian pig fat. The NIRS system that was developed allows for the analysis of live pigs and carcasses to predict fatty acid profiles without interrupting the processing system. 

Savenije et al. [78] studied three different breeds of pigs, and the accuracy and robustness of the calibration on independent samples were validated. Drip loss, colour value, pH, and IMF were investigated in chops. It was found that the breed of pig did not influence the accuracy of the calibration, and IMF was determined with good accuracy.

The nutritional value of meat is related to its composition of AA, FA, minerals, and vitamins. Although, excessive consumption of meat, especially red meat, can lead to diseases such as hypertension. Most of these diseases are related to the FA composition of meat, so awareness of this would be of paramount importance from a consumer perspective. FA is determined by GC as a standard. Several studies on the determination/prediction of FA composition using NIRS technology have been reported in beef [79,80,81], pork [82,83,84,85,86], sheep [87,88], chicken [89], and rabbit [90]. When predicting small FAs, PUFAs are difficult from beef because the strong absorption effect of water in the IR range affects the detection of the component found in small amounts. 

Cheng et al. used NIR-HSI (1000–2200 nm) in combination with chemometrics to predict the degree of lipid oxidation in pork (TBARS) during frozen storage. An interesting phenomenon they discovered was that good results in predicting TBARS value also showed that the chemical modification of pork during frozen storage was highly significantly correlated with the size and distribution of ice crystals [91].

To improve predictions, researchers are trying several models. For instance, Vasconcelos et al. [92] found that the SVMR-Poly predictive model cannot predict with high accuracy the aw, moisture, ash, fat, protein, pigments, collagen, WHC (water holding capacity), RT (raw texture), and CT (cooked texture) analyzed by NIR.

Besides this, the use of multi-techniques integrating NIRS, Computer Vision (CV), and Electronic Nose (EN) to significantly enhance the prediction performance has also been explored, particularly for TVB-N content in pork. The TVB-N content of meat serves as an important reference for evaluating its freshness alongside organoleptic qualifications and chemical parameters. In this study, NIRS, CV, and EN were combined to determine TVB-N, while BP-ANN was employed for the prediction model [93].

The ability to predict the protein, fat, and moisture content of meat samples by NIR spectroscopy was discussed in previous reviews. Visible/near-infrared (Vis/NIR) spectroscopy for online prediction of fresh pork meat quality characteristics (IMF, protein, and water content, pH, and shear force value) was tested. It was found that the 1st derivative for the quality parameters they investigated eliminated the negative effect of translation errors, independent of the wavelength of the reflectance spectra caused by varying slice thicknesses, and when combined with MSC, this derivative gave the best calibration results [94].

Barbin et al. took hyperspectral images of whole and minced meat, determined protein, moisture, and fat content using classical methods, and then combined the spectral information with PLS. The results showed that PLS regression models developed from wavelengths associated with characteristics from ground samples predicted protein, moisture, and fat with reasonable accuracy, with a coefficient of determination R^2^_P_ > 0.88 [95].

#### 5.2.3. Lamb Meat

Additionally, the potential for predicting the organoleptic properties of lamb meat using the Vis/NIR technique was investigated. Samples were scored by sensory judges on a taste panel, with 25 extreme cases—best and worst—being selected. It was shown that NIRS could effectively discriminate samples with extreme sensory properties. The range between 890 and 1000 nm was identified as particularly useful for this, as it was found to significantly correlate with the water and IMF content of the meat samples [96]. Protein, being a key functional and nutritional component of meat and meat products, has been the focus of numerous studies involving the development of predictive NIR models. However, comparisons of the reported errors in protein measurements are often challenging, as these errors are expressed either as a percentage of fresh or dry matter and are determined through cross-validation or separate validation sample sets. In certain instances, only calibration errors are reported, which further complicates the evaluation of the model’s predictive accuracy.

#### 5.2.4. Poultry Meat

Marchi et al. examined whole chicken breasts 48 h after slaughter, aiming to explore the capability of NIR technology in estimating the physical and chromatic characteristics of chicken meat. This was achieved by directly applying a fibre-optic probe to the breast muscle. Their research revealed that the prediction of a CIE index was closely related to absorption at wavelengths between 1230 and 1400 nm. Furthermore, the prediction of the a* value, which is influenced by meat water content and myoglobin concentration, was effectively linked to the visible NIR regions [97].

Viljoen et al. developed a NIRS method for predicting the chemical composition of freeze-dried lamb meat [98]. For this purpose, samples were scanned at wavelengths ranging from 1100 to 2500 nm. It was found that the freeze-dried samples provided more accurate calibrations than previously published research results, likely due to the homogeneous nature of the samples and the absence of moisture. Although, it was emphasized that changes in temperature also affect the chemical composition of the samples. The model developed was deemed suitable for the determination of K, P, Na, Mg, Fe, and Zn minerals [99]. Additionally, Dixit et al. [100] developed a method to predict the IMF content of lyophilized ground lamb.

Research confirmed that NIR can be successfully used to estimate the chemical composition of fresh and lyophilized minced meat. In addition to chemical composition, they were also able to distinguish the AA (amino acid) profile depending on the genetic group. The most important amino acids used to distinguish the genetic groups were alanine, aspartic acid, and methionine [101].

#### 5.2.5. Adulteration and Classification

The issue of meat authenticity concerns not only consumers but also producers and distributors. Meat adulteration can cause harm not only to human health but can also raise religious concerns, as in some countries pork is considered an unclean animal. To protect consumers and prevent unfair competition in the meat trade, fast and reliable methods must be applied to detect adulteration [102].

Kuswandi et al. [103] developed a method for detecting adulteration in beef meatballs with pork using NIR spectra coupled with chemometric techniques (PLS and LDA). A quantitative prediction of pork adulteration in beef meatballs can be achieved using the PLS model built on first derivative spectra. Meanwhile, a classification of clean and pork-adulterated beef meatballs can be performed using the LDA model.

Schmutzler et al. [104] developed a method for detecting adulteration in pork meat. In developing this method, adulterations between 10 and 51% were analyzed. Principal component analyses (PCA) were designed for each setting using pre-processing steps of the data, including wavelength selection, variance corrections and spectral data derivation. PCA scores were used as input data for classification and validation using support vector machines (SVM). Measurements were also performed directly through polymer packing of the samples and compared to measurements through quartz slides. Meat and fat adulteration were detected at contamination levels as low as 10% in both laboratory and industrial fibre optic set-ups, with measurements made through quartz and polymer packaging.

Consumers are placing more and more emphasis on quality-related attributes, such as animal breed, husbandry, feeding, etc. For this reason, there is a need for a method to ensure that foodstuffs are classified in this respect. Clear differences in location, feeding conditions, breed, and soil characteristics may contribute to variations in the organic composition (protein, fat, and carbohydrate) and structure of meat. This information is reflected in the NIR spectra measured at different locations. NIR spectroscopy was used to identify breed and age, in this case, to compare aspects such as colour, fat, protein, and moisture, as well as technological properties, e.g., cooking loss and purge loss [105], in another study, Iberian pig half carcasses were analyzed after slaughter according to three feeding methods using a microelectromechanical system (MEMS) spectrometer. The classification results for Iberian pigs fed with three different feeds were 93.9%, 96.4%, and 60.6% [106]. 

The classification of lambs from pastoral and agricultural regions was investigated. D-PLS and LDA analyses correctly classified 100% of samples from both pastoral and agricultural regions, with overall correct classification rates of 88.9% and 75% for the five different regional samples [107].

Researchers tried to classify meat according to its origin, and a NIRS method was developed to investigate the origin of chicken meat. The spectra were used to distinguish between fresh and thawed meat and the growing conditions of the chickens (rearing method and feeding) using the RSDE (random subspace discriminant ensemble) method, achieving a classification accuracy of over 95% [108].

In addition, studies have been carried out to classify post-harvest techniques, e.g., storage conditions [109,110].

The possibility for NIR-based discrimination of meats originating from the extensively-reared autochthonous breed of Mangalica and intensively-reared commercial genotypes (Landrace, Large White, Landrace × Large White crossbreed) was investigated. The classification is based on the considerable difference between the intramuscular fat content of Mangalica and intensively-reared meats (average of 19.1 DM% vs. 9.3 DM%, resp.) [111].

#### 5.2.6. Meat Products

Processing plays a major role in NIR analyses of meat and meat products, as researchers have found that meat prepared by mincing is more homogeneous than meat tested whole. The energy absorbed is lower when examining minced meat, thus producing a higher reflectance that is easier to measure [112,113]. The ability of NIR techniques to discriminate pork chop roasting methods based on other methods (roasting and confit) and conditions (temperature and time) was demonstrated by González-Mohino et al. [114].

A NIRS model for the determination of hydroxyproline content in pork sausages and dry-cured beef using a remote reflectance fibre-optic probe was developed by González-Martín et al. [115]. The method allowed for the determination of hydroxyproline in the range of 0–0.74%.

The use of near-infrared spectroscopy (NIR) to predict the drying parameters (aw, moisture, and NaCl) of fermented sausage was evaluated by Collell et al. Both methods demonstrated high predictive accuracy, suitable for online monitoring [116]. 

The use of NIR spectroscopy combined with chemometric analyses to detect the treatment of dry fermented sausage with ionizing radiation was investigated by Varrá et al. [117]. The irradiation of food products, which can increase shelf life, is allowed up to a maximum dose of 10 kGy according to Directive 1999/3/EC. The study demonstrated the feasibility of simple and rapid detection of dry fermented sausages treated with irradiation doses of 0.5–3 kGy through chemometric analysis combined with NIR spectroscopy. OPLS-DA results showed 100% clear discrimination of the samples by irradiation treatment.

A near-infrared spectroscopy technique was developed to monitor the production process (curing) of an alternative salted ham. In this study, lean cuts of meat were salted on a tray, and the fatty cuts of meat were salted in a tub. During the curing process of lean hams, the accurate determination of moisture and protein parameters was enabled by the developed calibration models, with RPDs of 5.8 and 3.4, respectively, being achieved. For fatty ham, good predictive capacity was archived for protein, water activity, and proteolysis index parameters, with values ranging between 2.5 and 3, while moisture was well predicted with an RPD of 10.4 [118].

Meat products from meat depend on external factors such as rearing, feeding, sanitary and environmental conditions, transport, preslaughter conditions and post-slaughter storage. Internal factors such as genetics, age, slaughter weight, sex and physiological condition also affect quality [92].

Building on this understanding of the factors affecting meat quality, further research has focused on developing more precise models to assess key chemical parameters in meat.

A model for the determination of major chemical parameters of prad-based meat products was developed by Ritthiruangdej et al. [119]. Good results were achieved using a PLS regression calibration model with MSC pretreatment in predicting protein (RPD = 7.6), moisture (RPD = 9.8), and fat content (RPD = 9.5). Although, the determination of residual nitrite content proved to be challenging.

Texture problems can also arise in the production of dry-cured hams. A crust may form on the surface of the ham, reducing the possibility of drying out [120]. The resulting calibration models allow for the monitoring of the resting and drying process, which may be useful in avoiding crust formation [121].

A NIRS method was developed to predict the sodium content of dry-cured ham slices. As reference data, the sodium content of the sample was determined by ICP-AES. PLS regression was used to perform the calibration. The models gave acceptable results with cross-validation correlation coefficients (R^2^_CV_) ranging from 86.2 to 90.2%. The prediction capacity achieved in external validation was 3.63 with a standard prediction error of 0.12% Na [122].

The prediction of storage temperature and storage time was investigated. It was found that a handheld NIRS instrument combined with PLS-DA could be used as a suitable tool to discriminate the temperature at which sliced Duroc dry-cured ham was preserved (4 °C vs. 20 °C). In addition, reliable discriminatory models were obtained to predict the storage time of samples (under conventional refrigeration conditions or at room temperature) at 0, 3, and 5 months. These results have practical implications for self-monitoring and logistics [110].

In summary, these advancements in NIR spectroscopy, from predicting protein and moisture content to distinguishing cooking methods and monitoring sodium levels, demonstrate the versatility and growing precision of the technique in meat quality analysis. While challenges remain, such as improving the accuracy for certain parameters and accounting for sample preparation, the continued refinement of calibration models and processing approaches highlights the potential of NIRS as a reliable tool for the meat industry.

The research results related to meat and meat products are summarized in Table 3 and Table 4.

### 5.3. Milk and Dairy Products

Milk is one of the most important sources of nutrients widely consumed around the world, either in its natural form or through dairy products. Therefore, in the dairy industry, quality and safety control is essential to ensure that products meet legal requirements and customer needs. 

Milk is a nutrient-rich complex liquid, 87% of which is water, so it also acts as a solvent for various nutrients. The remaining 13% contains nutrients that are essential for human health, such as lactose, which makes up about 4–5% of milk, is critical for supplying energy, and contributes to the distinctive taste of dairy products. Proteins make up about 3% of the composition of milk and can be divided into two classes: caseins and whey proteins. Caseins make up 80% of milk proteins, are insoluble, and form complexes called micelles, which can trap calcium and phosphorus. Whey proteins, which make up about 20% of milk proteins, are soluble, and are known for their high levels of branched-chain amino acids, which support muscle maintenance and immune function. Milk contains between 3% and 4% fat, 98% of which is made up of triglycerides, with more than 400 different fatty acids. This fat fraction is predominantly composed of 70% saturated fatty acids, including significant amounts of palmitic, myristic, and stearic acid, and 30% unsaturated fatty acids, mainly oleic acid. Milk also contains a small proportion of polyunsaturated fatty acids such as linoleic acid and alpha-linolenic acid. Milk fat includes bioactive compounds as well, such as conjugated linoleic acid, known for its cardiovascular support and anti-cancer effects. Although the micronutrient composition of milk is significantly influenced by the cow’s diet and the conditions of dairy technology, in general, it has a mineral content of about 0.8%, the main constituents of which are calcium and phosphorus, essential for bone and tooth structure and metabolic processes. In addition, milk provides significant amounts of magnesium and zinc selenium, supporting a range of physiological functions. Of the vitamins, both fat-soluble vitamins (A, D, E) and water-soluble B complex vitamins are found in milk, in total 0.1% [145].

One of the most prominent applications of near-infrared spectroscopy is in the milk and dairy industry, dating back to the late 1970s. This chapter reviews publications on the use of NIR in the dairy industry from 2004 to 2024. Most of the publications in this period deal with the quality assessment of milk and dairy products. In these cases, an estimation model is built to quantify the major compositional parameters, including protein [146,147,148,149,150,151,152,153,154,155,156], fat [146,147,148,149,150,152,153,154,155,156,157,158,159,160,161], lactose [146,147,148,149,152,153,154,155,162,163,164,165], moisture [166,167,168] and other quality attributes, like fatty acids [149,169,170,171,172,173,174], titratable acidity [163,175], pH [147,163,168], somatic cell count [146,149,155,160,176], vitamins [162,170], minerals [177,178,179], freezing point [147,155], density [147] in the final product or during dairy technological steps for monitoring and quality control purposes. In addition, there are several studies on the use of NIR in the detection of adulteration of dairy products, the classification of the products tested, and the quantification of the adulterant. Some publications report on NIR methods used to identify the animal species or geographical origin of dairy products. The key publications on the application of NIR in the dairy industry are summarized in Table 5.

There are some comprehensive reviews on the application of NIR in the dairy industry, providing valuable information for the quantification of major and minor components of milk and dairy products. The potential of non-destructive techniques for the determination of the quality of dairy products was presented by Karoui et al. [180]. Wang et al. [181] summarized the research developments of NIR in the field of liquid foods. A recent review discussed the use of multivariate chemometric modelling of NIR, MIR, fluorescence and Raman spectral data and the use of data fusion strategies for milk analyses [182]. 

Most of the publications in the period 2004–2024 focused on analyzing different milks, as shown in the Table 4.

Melfsen et al. [149] published their results about robustness of NIR calibration models for the prediction of milk fat, protein, and lactose. Different calibration models (fully random internal calibration, internal calibration, external calibration, and a combination of internal and external datasets) and different validations (internal and external) were used to estimate fat, protein, and lactose content. Excellent calibration results were obtained in the case of the fully random internal calibration sets; RPD values of around 10, 5 and 3 for the prediction of fat, protein, and lactose, respectively, were achieved. An application of internal calibration showed much poorer prediction results, especially for the prediction of protein and lactose. They also found that the prediction accuracy improved when a validation was conducted on the spectra of the external dataset. The effect of temperature on the accuracy of FT-NIR measurements was investigated by Dvorák et al. [183]. The samples were measured in a reflectance mode at 18, 20, 22, 24, and 40 °C. The results underlined that temperatures do not generally affect dry matter and lactose content in milk; responses to changes in temperature are probably caused by changes in the composition of fats and proteins. Therefore, milk should be measured at the same temperature as the calibrated instruments. Benedictis et al. [184] demonstrated an approach for optimizing near-infrared spectra with experiment designs. The investigated factors are layer thickness, number of scans, and temperature during measurement. The response variables were absorption intensity, signal-to-noise ratio, and reproducibility of the spectra. Optimized factorial combinations have been found to be 0.5 mm layer thickness, 64 scans, and 25 °C ambient temperature, for liquid milk measurements. Pu et al. [185] published a review article about advances in portable and handheld NIRs, focusing on recent developments and their latest applications in the field of dairy, including chemical composition, on-site quality detection, and safety assurances in milk, cheese, and dairy powders. Guerra et al. [155] reviewed the application of a short-wave pocket-sized near-infrared spectrophotometer to predict fat, protein, casein, lactose, urea, freezing point, SCC, and fat to protein ratio in cow milk. A total of 331 individual milk samples were collected for chemical determination and spectral collection by using two pocket-sized NIR spectrophotometers working in the range of 740 to 1070 nm, and modified partial least squares regression models were developed. The results revealed that short-wave pocket-size NIR spectrophotometers have the potential to predict milk fat, protein, casein, and fat-to-ratio while the poor models obtained for lactose, SCC, MUN, and freezing point could be related to a lack of information in this short-wave NIR region. Portable NIR was used by Yang et al. [153] to determine fat, protein, lactose, and total solids in milk using PLSR models. The effect of several spectral pre-processing methods on prediction performance were evaluated, and the results indicated that Savitzky–Golay smoothing (SGS) and SGS combined with standard normal variate proved the best spectral pretreatment method for raw milk and for homogenized milk, respectively. 

The characterization of milk with NIR is not limited to estimating the quantity of the main constituents. Allende-Prieto et al. [186] used the NIR to detect bacteria in milk. The combination of PCA and PLS-DA was used to distinguish the contaminated and the uncontaminated samples. The results suggested that NIR technology can be used to accurately classify contaminated and uncontaminated milk samples, regardless of the type of bacteria causing contamination, even at low concentrations. However, the spectral analysis was not capable of distinguishing between the four studied contaminating bacteria. Tsenkova et al. [176] summarized their results about disease diagnosis and pathogen identification in milk samples. They have developed spectroscopic models for the simultaneous measurement of somatic cell count and electrical conductivity, as well as for identification of the main mastitis-causing bacterial pathogens in milk. These results highlight the potential of NIR spectroscopy as a powerful technology for in vivo health monitoring, disease diagnostics at the molecular level, and bacterial identification. 

A good example of the use of the NIR technique for monitoring specific processes in dairy technologies is found in the work of Grassi et al. [187] about monitoring milk renneting during cheese manufacturing. A multivariate curve resolution optimized by alternating least squares (MCR-ALS) was used for data analysis and development of multivariate statistical process control (MSPC) charts. The models described the coagulation processes (explained variance ≥99.93%; lack of fit <0.63%; and standard deviation of the residuals <0.0067) well. Lactic acid fermentation process monitoring was investigated by the same research team Grassi et al. [188]. Some rheological and conventional quality parameters (microbial counts, pH, titratable acidity, lactose, galactose, and lactic acid concentrations) were used as reference values to assess the findings with FT-NIR spectroscopy. The results showed that near-infrared spectroscopy is a useful tool for real-time assessment of curd development during fermentation. Lyndgaard et al. [189] published a paper which focuses on the extraction of real-time, meaningful information from NIR reflectance measurements of coagulating milk. 

In addition to milk, there have also been a few publications on the study of cheese. A comprehensive review regarding the application of NIR for predicting the chemical composition of cheese was written by Bittante et al., by providing the results of 71 papers. In addition to estimating the quantification of the main components, NIR was widely used in cheese to monitor technological processes and determine specific properties. Cheese ripeness was predicted based on the ratio of water-soluble nitrogen to total nitrogen as an index of cheese maturity by Currò et al. [190]. The prediction of sensory attributes of cheese via NIR was studied by González-Martín et al. [191]. Nicolau et al. published an application of NIR for the estimation of clotting and cutting times in sheep cheese manufacture. [192].

Comparatively few publications have been published on NIR analysis of other dairy products such as yoghurts and butters. Butter is mainly measured for fat and fatty acids [159,173], while yoghurts are measured for fat [156,160], protein [156], sugar [162] and pH [160] using NIR. 

NIR is also widely used in the dairy industry to detect adulteration. According to a 2013 European Parliament report, milk was one of the four foodstuffs considered to be the most common target of economically-motivated adulteration. Milk and dairy products are foods with high nutritional value, largely consumed by the general population and play an important role in the diets of certain consumer groups, notably children and pregnant women. Due to their high demand and value, fraud in the dairy industry has become a widespread problem [193]. 

More review articles cover this topic, giving a good overview [180,193,194,195,196]. 

There have been reports of several types of in the dairy industry. Most of them can be detected by NIR, including dilution with water [197,198,199,200,201], addition of whey rennet [197,199,202], substitution of milk fat or protein [203,204,205], addition of fillers [202,203,206,207], substitution of milk from one species with a lower valued one [183,199,208,209,210,211], and addition of nitrogen-rich adulterants like melamine [206,207,212,213,214,215,216,217] or urea [198,199,206,207,218] to increase protein content.

The practise of mislabelling, either in terms of geographical origin or animal species origin, is also considered adulteration. Classification models based on NIR can distinguish dairy products by geographical origin [219,220,221] and animal species by origin [222], with high accuracy.

In conclusion, one of the most widespread uses of NIR is the qualification of milk and dairy products, monitoring of dairy technological processes, and detection of adulteration, with many present results and several future improvement opportunities.

The detailed data are summarized in Table 5 and Table 6.

**Table 5:** NIR test results for milk.

Sample	Investigated Parameter	Concentration Range	Chemometrics Data			References
			Pre-Treatment, Regression	R^2^	Root Mean Square Error	
Milk	Sugar—lactose, %	2.06–5.06	RS, PLS	0.83	0.26	[146]
		3.30–4.29	1st der., PLS	0.90	0.11	[147]
		2.92–5.22	transmittance, 1st der., OSC, RiPLS	0.883	0.115	[148]
		3.9–5.2	normalization, PLS	0.92	0.06	[149]
		3.09–4.70	ANN	0.8822	0.238	[163]
		3.98–5.1	MSC, 1st der., OSC, Rt-RiPLS	0.689	0.077	[152]
			Log base, 1st der., OSC, PH-RiPLS	0.644	0.092	
	raw	3.97–4.89	SGS, PLS	0.78	0.11	[153]
	homogenized		SGS, SNV, PLS	0.71	0.12	
		4.14–5.25	RS, PLS	0.13	0.87 (RPD)	[155]
	Sugar, %		SNV, MC, PDS, PLS			[165]
	sucrose	14.20–43.69		0.7973	5.04	
	lactose	0.000–38.99		0.9411	4.22	
	Lactose free, %	0–1	1st der., SNV, PLS	0.79	0.1984	[164]
	Carbohydrate, g/100 mL	2.5–13.5	SGS, 2nd der., PLS	0.883	0.639	[154]
	Fat, %	1.01–7.39	RS, PLS	0.95	0.25	[146]
		5.66–11.06	1st der., PLS	0.73	0.66	[147]
		2.72–7.94	transmittance, SNV, GA-PLS	0.997	0.043	[148]
		0.7–12.3	normalization, PLS	0.998	0.09	[149]
	Gerber Röse-Gottlieb	0.13–7.25	PLS	0.98	0.232	[158]
				0.992	0.148	
		0.1–3.7	SGS, 2nd der., PLS	0.969	0.216	[154]
		0–3.9	1st der., MSC, PLS	0.98	0.002	[157]
		1.54–6.25	DT, 2nd der., OSC, PH-RiPLS	0.989	0.078	[152]
			DT, 2nd der., Rt-FiPLS	0.989	0.083	
	raw homogenized	2.09–5.76	SGS, PLS	0.97	0.18	[153]
			SGS, SNV, PLS	0.99	0.11	
		1.03–5.02	MC, SNV, SGS, SSDL	0.95	0.22	[161]
		1.86–5.96	DT, PLS	0.93	3.73 (RPD)	[155]
Milk	Fatty acids, mg/mL		SGS, SNV, SVM			[174]
	C4:0	0.08–0.325		0.87	0.03	
	C6:0	0.004–0.21		0.83	0.02	
	C14:0	0.019–1.208		0.82	0.11	
	C16:0	0.044–3.381		0.74	0.35	
	C18:1C9	0.048–1.75		0.84	0.12	
	SFA	0.128–6.553		0.83	0.59	
	MUFA	0.056–2.128		0.87	0.15	
	SCFA	0.011–0.505		0.88	0.04	
	BCFA	0.004–0.141		0.83	0.01	
	PUFA. mg/g: C18:2	0.63–59.88	2nd der., MSC, MPLS	0.58	8.40	[169]
	C22:6	0.05–0.16		0.75	0.01	
	ω6	0.63–60.09		0.58	8.41	
	ω6/ω3	3.51–12.34		0.76	0.94	
	Total fatty acid, %		SNV, DT, MSC, 1st der., MPLS			[170]
	SFA	36.74–78.06		0.96	2.03	
	MUFA	17.73–50.65		0.81	4.13	
	PUFA	2.02–14.08		0.80	0.95	
	trans FA	0.35–29.05		0.84	2.95	
	SFA	59.7–89.5	1st der., PLS	0.72	1.86	[171]
	MUFA	9.30–38.2	2nd der., SNV, PLS	0.83	2.12	
	PUFA	1.21–7.20	2nd der., SNV, PLS	0.55	1.97	
	SCFA	2.97–9.87	2nd der., SNV, PLS	0.87	2.25	
	MCFA	40.61–71.77	2nd der., SNV, PLS	0.43	1.79	
	Acidity, °T	16.0–24.8	ANN	0.9709	0.380	[163]
	pH	6.50–7.01	1st der., PLS	0.42	0.105	[147]
	Protein, %	2.77–4.38	RS, PLS	0.72	0.15	[146]
		5.30–7.00	1st der., PLS	0.84	0.21	[147]
		2.65–5.01	reflectance, 1st der., OSC, GA-PLS	0.959	0.099	[148]
		2.4–4.0	normalization, PLS	0.98	0.05	[149]
		6.45–6.95	ANN	0.9645	0.0202	[163]
		2.61–4.77	SNV, PLS	0.77	1.84 (RPD)	[155]
Milk	raw	2.88–3.59	MSC, PLSR-UVE-PLS	0.92	0.06	[151]
	homogenized			0.96	0.04	
		2.63–4.34	SNV, 1st der., OSC, PH-FiPLS	0.947	0.08	[152]
			DT, 2nd der., OSC, Rt-RiPLS	0.894	0.11	
	raw	2.94–4.33	SGS, PLS	0.85	0.16	[153]
	homogenized		SGS, SNV, PLS	0.90	0.13	
		1.3–7	SGS, 2nd der., PLS	0.883	0.290	[154]
	Casein, %	2.03–3.70	DT, PLS	0.70	1.80 (RPD)	[155]
	Urea, mg/100 mL	10.41–15.73	RS, PLS	0.53	1.5	[146]
		13.6–33.2	1st der., OSC, GA, PLS, RiPLS	n.i	RPD < 1.2	[148]
		12.1–38.0	normalization, PLS	0.82	1.932	[149]
		5.10–31.70	SNV, DT, PLS	0.43	1.18 (RPD)	[155]
	Freezing pont, °C	−0.66–−0.47	1st der., PLS	0.90	0.02	[147]
		−0.503–−0.548.	SNV, PLS	0.22	0.64 (RPD)	[155]
	SCC, cell/μL	7.00–2837	RS, PLS	0.03	0.22 (RPD)	
	Log SCC, log cells/mL	3.78–5.84	RS, PLS	0.68	0.28	[146]
		3.5–6.0	normalization, PLS	0.85	0.18	[149]
	Fat:protein ratio	0.82–3.43	DT	0.71	1.74	[155]
	Total solid content, %	9.42–15.12				[153]
	raw		SGS, PLS	0.96	0.28	
	homogenized		SGS, SNV, PLS	0.98	0.21	
	Carotenoids, μg/mL; cis9-β-carotene, β-cryptoxanthin	0.11–1.04	SNV, MSC, DT, 1st der., 2nd der., MPLS	>0.50	0.01	[170]
	Vitamin A, μg retinol/mL	0.03–1.33	SNV, DT, MSC, MPLS	0.34	0.15	
	Density kg/m^3^	1029.66–1039.94	1st der., PLS	0.88	1.07	[147]
	Fat free dry matter, %	9.53–12.45	1st der., PLS	0.90	0.29	
	Ash, %	0.87–1.14	1st der., PLS	0.89	0.03	
	Contamination	4–9 log cfu/mL	MC, SNV, PLS			[223]
	*E. coli*			0.936	0.284	
	*P. aeruginosa*			0.597	0.0202	
	*E. coli* + *P. aeruginosa*			0.8822	0.584	
Milk	Progesterone (real-time), ng/mL	0.10–12.61	2nd der., PLS	0.93	1.06	[224]
		3.92–21.37		0.89	1.22	
		0.03–10.78		0.93	0.92	
		0.01–4.86		0.91	0.43	
	Classification	adulterated	MSC, 2nd der., DPLS	Accuracy: 100%		[197]
		lactose (no or yes)	PLS-DA	Sensitivity: 90% or 100% Specificity: 100% or 90%		[154]
		*E. coli, P. aeruginisa*	MC, SNV, PLS-DA	correct prediction 99%		[223]
		*Salmonella* sp.	2nd der., PLS-DA	Sensitivity: 100% Specificity: 100%		[225]
		geographical origin	SGS, FUDT, kNN	accuracy 98.67%		[220]
		geographical origin	SGS, SNV, kNN, FD-LDA	accuracy: 97.33%		[221]
		water	EPO, RSDE	accuracy: 98%; reliability: 98%		[200]
		water	RS, DTC or RFC, or kNN	accuracy: 100%		[201]
		melamine	RS, PLS-DA	Sensitivity and specificity 100%		[214]
		melamine	OPLS-DA	R^2^X: 0.996, R^2^Y: 0.964, Q^2^: 0.933		[217]
	Adulteration with water, %	1–97	MSC, PLS	0.997	2.159	[197]
		0–70	MC, SNV, SGS, SSDL	0.80	0.12	[161]
		1–30	RS, BRT	0.95	0.58	[200]
		0–40%	RS, kNN, SVML	0.999	0.353	[201]
	Adulteration with melamine, %	0.001–0.29	OCPLS	Sensitivity. specificity, accuracy 90%.; 88%; 89%		[215]
		1–20	SNV, PLS	0.98–2.99 matrix dependent		[217]
	Adulteration with whey, %	2.15–48.40	MSC, PLS	0.999	0.244	[197]
		0.01–0.29	1st der., UVE-PLS	0.97	0.015	[214]
Flavoured milk drink	Moisture, %	77.13–80.83	2nd der., PLS and ANN	0.982; 0.989	0.778; 0.744	[168]
	Water activity	0.963–0.982	2nd der., PLS and ANN	0.996; 0.984	0.764; 0.725	
	Total soluble solids, %	19.16–22.86	2nd der., PLS and ANN	0.687; 0.946	0.727; 0.754	
	pH	6.35–6.66	2nd der., PLS and ANN	0.955; 0.955	0.723; 0.711	
	Colour, BI	15.915–19.630	2nd der., PLS and ANN	0.988; 0.978	0.703; 0.713	
Milk brands	Classification	MSC, EELM		accuracy: 100%		[226]
		SGS, PCA, LDA, iNLDA, FiNLDA, KNN		accuracy: 74.7% (LDA), 88% (iNLDA), 94.76% (FiNLDA)		[227]
Human milk	Moisture, %	83.18–94.26	2nd der., PLS	0.90	0.5149	[167]
	Fat, g/100 mL	1.56–6.37	2nd der., PLS	0.70	0.4274	
		0.51–5.30	RS, PLS	0.841	0.51	[228]
	Ash	0.09–0.40	2nd der., PLS	0.64	0.0507	[167]
	Protein, g/100 mL	0.45–5.04	2nd der., PLS	0.70	0.3581	
		0.27–2.50	RS, PLS	0.512	0.21	[228]
	Carbohydrates, g/100 mL	2.73–10.63	2nd der., PLS	0.70	0.6063	[167]
		2.34–8.80	RS, PLS	0.741	1.35	[228]
	Total solid content, g/100 mL	3.27–14.60	RS, PLS	0.686	2.42	
	Energy, kcal/100 mL	33.80–87.04	2nd der., PLS	0.83	3.7848	[167]
		15.60–86.00	SNV, 1st der., PLS	0.830	9.60	[228]
	Classification—lactation phases	Colostrum Transition Mature	MSC, PLS-DA	Sensitivity, Specificity 87.5%, 90.3% 56.3%, 71.9% 93.8%, 93.8%		[167]
		Bovine colostrum adulterated milk	MSC, PLS-DA	Sensitivity, Specificity, Accuracy 84.62%, 100%, 94.74%		[210]
Infant formula	Moisture, %	2–13	PLS	0.99	0.62	[229]
	Storage time, months	0–3–6–12	PLS	0.97	n.i.	[230]
	FAST index	1.88–21.54	RS, PLS	0.78	n.i.	
	Soluble protein, %	0.77–5.29	RS, PLS	0.86	n.i.	
	Fat, %	24.94–28.65	SNV, PLS	0.74	n.i.	
	SFF, %	0.02–2.60	PLS	0.88	n.i.	
	Classification		PLS-DA	accuracy 100%		
	Adulteration with melamine, μg/g	17.3–2000	1st der., MC-OSC, ANN, SVR, LS-SVM	n.i.	6.1	[212]
Milk powder	Carbohydrates, %	50.73–60.28	SNV, LS-SVM	0.982	0.384	[231]
	Fat, %	15.93–21.80	RS, LS-SVM	0.981	0.247	
	Protein, %	14.82–18.14	SNV, LS-SVM	0.984	0.148	
		18.0–32.6	SNV, MRMR-PLS	0.99	0.37	[232]
	Moisture, %	4–10	MC, 2nd der., PLS	0.9822	0.1730	[166]
	Mineral content, Ca-mg/100 g	243.1–722.8	SGS, SNV, UVE-SPA-LS-SVM	0.85	0.18	[178]
	Classification	brands	MRMR-PLS-DA	accuracy: 100%		[232]
Milk powder	Adulteration, %	14.6–2000	1st der., MC-OSC, Poly-PLS	n.i.	0.28	[212]
	melamine, μg/g					
	corn starch wheat flour	0–30	MSC, 1st der., PLS 2nd der., MSC, PLS	0.74 0.82	9.70 8.38	[202]
Goat milk	Lactose, %	2.06–5.06	RS, PLS	0.935	0.050	[175]
	Fat, %	2.27–5.61	RS, PLS	0.924	0.154	[175]
		0.9–4.2	13MM-LBC, iSPA-PLS	0.96	0.20	[209]
	Protein, %	2.33–3.41	RS, PLS	0.888	0.111	[175]
		2.95–5.03	13MM-BO-LBC, PLS	0.96	0.047	[209]
	Total solid content, %	10.30–13.76	RS, PLS	0.899	0.334	[175]
	Fat free solids, %	7.19–8.81	RS, PLS	0.812	0.191	
	Freezing point, °C	−0.599–−0.527	RS, PLS	0.833	0.005	
	Titratable acidity, °SH	4.60–8.20	RS, PLS	0.878	0.469	
	pH	5.69–6.92	RS, PLS	0.703	0.076	
	Adulteration,	1.0154–100	SPA, PLS	0.9955	3.66	[209]
	cow milk, %					
	water, urea, bovine, whey or cow milk	0–20%	1st der., MC, SNC, PLS-DA	for authentication and adulteration sensitivity and specificity 100%		[199]
	Classification	adulterated	PLS-DA	accuracy: 100%		[209]
Goat milk powder	Adulteration, %					[207]
	urea	0.5–10	area normalization, PLS	0.992	0.321	
	melamine	0.01–10	Area normalization, PLS	1.000	0.042	
	starch	1–30	smoothing, PLS	1.000	0.139	
Goat dairy products	adulteration with cow milk, %	10; 15; 20%	MC, 2nd der., PLS-DA with iPLS	Sensitivity, specificity 100% for both sample groups		[211]
yoghurt						
cheese						
cheese		0–50	PLS	0.783	2.454	[183]
Camel milk	Adulteration with cow milk, %	0–20	1st der., PLS	0.92	1.32	[208]
	Classification	pure or adulterated	PLS-DA	0.97	0.08	
Plant milk	Sugar, % (glucose)	0.5–7.6	1st der, MNSN, iPLS	0.84	0.98	

**Table 6:** NIR test results for dairy products.

Sample	Investigated Parameter	Concentration Range	Chemometrics Data			References
			Pre-Treatment, Regression	R^2^	Root Mean Square Error	
Cheese	Fat, %		SNV, MPLS MSC, MPLS			[191]
	summer	25.55–50.97		0.936	1.68	
	winter	19.97–61.29		0.871	3.23	
	lyophilized	19.1–55.6	MSC, 1st der., PLS	0.99	1.0	[150]
	Total fatty acid, %		SNV, PLS; SVM			[172]
	total FA	47.57–472.44		0.86; 0.59	28.87; 24.40	
	SFA	33.22–325.04		0.84; 0.88	21.66; 18.32	
	MUFA	10.02–114.1		0.75; 0.83	9.11; 7.47	
	PUFA	0.00–10.15		0.0; 0.1	2.78; 2.32	
	SCFA	2.54–26.95		0.80; 0.89	1.94; 1.36	
	MCFA	7.14–55.51		0.22; 0.55	5.34; 4.88	
	BUFA	0.9–5.29		0.78; 0.79	0.61; 0.55	
	Protein, %—lyophilized	24.7–60.7	SLS, SGS, 1st der., PLS	0.972	1.4	[150]
	Minerals, %		PLS			[179]
	Ca	0.229–0.510		0.75	0.02	
	K	0.023–0.167		0.37	0.17	
	Mg	0.009–0.020		0.82	0.00	
	Na	0.024–0.290		0.89	0.02	
	P	0.187–0.370		0.82	0.01	
	Classification	geographical origin	normalization, FDA	accuracy:		[219]
				Austrian: 100%		
				Finnish: 66.7%		
				German: 76.9%		
				French: 83.3%		
				Swiss: 94.7%		
		summer or winter	DPLS	Accuracy 97 and 96%		[191]
		Species of origin	SNV, SG, PCA	Accuracy 76%		[222]
Butter cheese	Classification		PLS-DA	Accuracy 94.44%		[233]
	Adulteration with soybean oil, %	5–100	RS, PLS	0.941	7.202	
Yoghurt	Fat, %	0.12–14.69	PLS	0.978	0.968	[160]
		2.6–4.4	2nd der., SNV, MC, PLS	0.990	0.25	[156]
	Sugar, %	10.75–13.25	MSC, SGS, PC-ANN	0.91	0.41	[162]
Yoghurt	Lactose free, %	0–1	1st der., SNV, PLS	0.98	0.0609	[164]
	Protein, %	3.2–3.5	2nd der., SNV, MC, PLS	0.80	0.16	[156]
	Total solid content, %	10.32–22.48	PLS	0.989	0.46	[160]
	Titratable acidity, °SH	11.88–58.91	PLS	0.979	2.47	
	pH	4.00–4.24	PLS	0.788	0.038	
		3.97–4.27	MSC, SGS, PLS	0.90	0.04	[162]
	Adulteration, %		SNV, OCPLS	Sensitivity: 90% Specificity: 91.9%		[203]
	edible gelatine	1–8				
	industrial gelatine	0.5–5				
	soy protein	0.5–5				
	Dry matter, %		1st der., iPLS			[159]
	reflectance	39.7–80.7		0.9730	2.224	
	transmittance	19.5–59.7		0.9488	2.2399	
	Fat, %		1st der., iPLS			
	reflectance	19.3–61.3		0.9772	1.9955	
	transmittance	17.7–57.5		0.9245	2.8545	
	Fatty acid—trans, %	0.24–0.62	PLS	0.98	0.46	[173]

### 5.4. Vegetable

The concept of a vegetable plant cannot be precisely defined. Generally, it refers to horticulturally-derived food with high biological value, rich in vitamins, mineral salts, and aromatic substances.

Detailed research results in vegetables are summarized in Table 7, Table 8, Table 9, Table 10, Table 11 and Table 12.

#### 5.4.1. Nightshades (Solanaceae)

The tomato (*Solanum lycopersicum*) is among the most extensively studied vegetables within the nightshade family.

NIR tests on tomatoes primarily focus on measuring the water-soluble dry matter (SSC) and titratable acid (TA) content of the fruit, as well as the SSC/TA value, which correlates with taste [234,235,236,237,238,239,240,241].

In addition to examining the SSC value, estimation models have been developed for the quick and non-destructive determination of glucose and fructose content in tomato samples, as well as the titratable acidity and the concentration of ascorbic acid and citric acid [242,243]. 

In addition to the quality attributes, evaluations of the texture and shelf life of extremely fragile tomatoes are also important [236,244].

To identify the most advantageous varieties, different classification models have been developed [236,239,242]. The PCA procedure was used for the classification, and the prediction models were performed using the PLS or wave number selection PLS regression method. The NIR technique combined with chemometric methods has been utilized to monitor quality alterations during storage. Classification tests have been performed on data from surface and liquid biopsies [245].

Quality characterization of tomatoes based on sensory attributes is time-consuming and very expensive. For this reason, it is not included in routine phenotyping. The root square error of prediction (RSEP) values for sensory properties (flavour and aroma intensity, texture, juiciness, and flouriness) were low. This can be explained by the fact that only 55 samples were tested for sensory properties. Despite the poor result, it is suggested that the estimation function can be improved for a larger sample population [243]. 

The NIR technique was successfully applied to the monitoring of lycopene concentration in addition to changes in quality attributes during ripening and storage [246,247,248]. In addition to the lycopene, titratable acidity (TA), and total phenolic content (TPC) of four dehydrated tomato varieties, a successful method was developed for determining the total sugar content and antioxidant capacity using near-infrared (NIR) spectroscopy. data obtained from the FRAP (Ferric Reducing Ability of Plasma), DPPH [2,2-di(4-tert-octylphenyl)-1-picrylhydrazyl], and ABTS (2,2′-Azinobis-(3-ethylbenzo-thiazoline-6-sulfonic acid) methods served as reference for antioxidant capacity determinations [249].

Duckena et al. [250] carried out comprehensive research on the NIR estimation of quality attributes in 80 different tomato cultivars. Besides the commonly tested dry matter content (DM) and taste index (SSC/TA), the method development was also expanded to include the estimation of lycopene, beta-carotene, total polyphenol, and flavonoid concentrations. 

Li et al. [251] proposed a novel prediction method utilizing segmentation of Vis-NIR spectral graph features to assess the activity of tomato polyphenol oxidase (PPO). The experimental outcomes indicated that this algorithm enhances the modelling effect, simplifies the modelling process, and increases the efficiency of the model [251].

Although, the use of various plant protection insecticide and the determination of their residues pose significant challenges in horticultural products. Typically, residues are measured using capillary gas chromatography (GC) and/or high-performance or ultraperformance liquid chromatography (HPLC or UPLC) coupled with mass spectrometry (MS) methods [252], following complex sample preparation. The NIR technique has been successfully applied to monitor lycopene.

Nazarloo 2021 et al. [253] conducted experiments to determine if the Vis/NIRS technique is suitable as a pesticide residue prediction method. Samples with different pesticide residual (Pre-Harvest Interval -PHI) concentrations of 2 per 1000 L were infected. The tests were performed at different times after spraying (without spraying, 2 h after spraying, after 2 days, after a week, after two weeks). At the same intervals, the tests were also carried out by washing the samples after spraying. GC-MS measurements were also used to verify residue concentrations. Using different variable selection and data management procedures, it was established that the most favourable correlation was given by the ANN model combined with the successive prediction algorithm (SPA) (Table 7). 

De Brito et al. [254] compiled a comprehensive summary manuscript presenting the determination of various tomato attributes using the NIR technique for the period 2010–2022.

**Table 7:** Overview of NIR Results for Tomato (*Solanum lycopersicum*).

Investigated Parameter		Concentration Range	Chemometrics Data			Ref.
			Pre-Treatment, Regression	R^2^	Root Mean Square Error	
Soluble solids content—SSC; °Brix		4.10–5.60	MN, MSC, PCA, PLS	0.80	0.210	[234]
		≈5.0–8.6	EPO, PLS	0.9072	0.302	[235]
		3.0–5.9	PCA, PLS	0.97	0.22	[236]
		n.i.	SGS, MSC, CARS, PLS	0.828	0.17	[237]
		n.i.	Smooth, PCA, BPN	0.8328	0.5711 (MAD)	[238]
		4.20–6.80	MSC, ELM	0.75	0.27	[239]
		3.0–6.7	MSC, PLSR	0.72	0.58	[240]
		3.2–6.8	OSC, PCA, PLSR	0.66	0.3227	[241]
		2.92–11.22	MC, Smooth, 2nd der., PLS	0.89	0.52	[242]
		4.20–11.60	2nd der., PLS	0.97	0.24	[243]
		3.87–3.99	PLS	0.93	0.366	[244]
		3.4–6.3	PLS	0.86–0.91	0.07–0.4	[246]
		4.4–6.1	SNV, 1st der., CARS; RF-PLS	0.812	0.211	[247]
Dry matter		4.55–13.15	PLSR	0.83	0.98	[250]
Textural property			MSC, PLS			[234]
Puncture test, N		1.96–6.08		0.902	0.35	
		1515–1612 (W_p_)	PLS, var. selection	0.92	579	[244]
		59.47–62.41 (F_int_)		0.91	14.2	
Firmness/hardness, N		11–23	PCA, PLS	0.71	0.7	[236]
Lycopene; mg/kg		≈50–118	EPO, PLS	0.8238	7.14	[235]
		79.4–287.5	MSC, PLSR	0.68	15.07	[240]
		3.69–50.05	PLS	0.73–0.84	0.91–0.92	[246]
‘Provence’		26.43–264.77	SNV, LARS-PLSR	0.95	7.34	[248]
‘Jingcai No. 8′		7.65–119.36		0.96	13.44	
mg/kg DW		240–415	RBF-NN	0.939	16.1	[249]
		0–83.8	PLSR	0.85	9.5	[250]
β-carotene		0.4–117.3	PLSR	0.85	10.1	
Glucose, g/100 g		4.68–39.12	Norm, 2nd der., PLS	0.87	2.91	[242]
		0.85–3.95	2nd der., PLS	0.98	0.09	[243]
Fructose, g/100 g		8.65–39.12	Norm, 2nd der., PLS	0.87	2.83	[242]
		1.06–3.82	2nd der., PLS	0.98	0.08	[243]
Ascorbic acid, mg/100 g		3.77–77.91	Norm, 2nd der., PLS	0.82	4.09	[242]
Citric acid, g/100 g		0.11–1.10	Norm, 2nd der., PLS	0.87	0.07	
Titratable acid—TA, %		0.1–1.7	PCA, PLS	0.89	0.20	[236]
		4.58–7.12	PLS	0.91	0.646	[244]
		0.5204–0.6320	PLS	0.74–0.77	0.0084–0.013	[246]
		4.19–6.15	PLS	0.88	0.18	[249]
Total sugar, %		21.3–43.4	PLS	0.972	1.22	
Dry matter		5.17–11.55	1st der., PLS	0.98	0.26	[243]
Taste (SSD/TA)		0.58–0.85	PLS	0.71	0.038	[244]
		2.8–22	PCA, PLS	0.94	1.5	[236]
		0.86–1.52	PLSR	0.77	0.1	[250]
Classification		Maturity of three species	MSC, PCA	Correctness 96.85%		[234]
		Maturity of five species		Classification success		[239]
			1st der., PCA	94.62%		
			SNV, PCA	76.92%		
			MSC, PCA	62.69%		
			MSC, PCA	78.85%		
			MSC, PCA	89.962%		
Storage condition		surface	SVM	AC = 92%; SENS = 86%; SPEC = 98%		[245]
		liquid biopsies		AC = 94%; SENS = 74%; SPEC = 95%		
Total polyphenol concentration (TPC), mg GAE/100 g		16.77–60.91	PLSR	0.5	6.33	[250]
g GAE/100 g DW		1.03–1.94	PLSR	0.954	0.08	[249]
Antioxidant activity μmol trolox/100 g DW	FRAP	57.9–118	RBF-NN	0.936	3.89	[249]
	DPPH	30.9–54.8		0.939	2.82	
	ABTS	47.7–108		0.968	3.44	
Flavonoid, mg QE/100 g		1.09–11.02	PLSR	0.8	1.31	[250]
Polyphenol oxidase (PPO) act., U/mL		8.0–45.0	ASR, MLR	0.97	1.99	[251]
Pesticide residues, mg/kg		n.d.–34.0	PCA, SPA-ANN	0.982	0.166	[253]

#### 5.4.2. Brassicas (Brassicaceae)

*Brassicaceae* family includes a variety of cabbages such as Chinese cabbage, cauliflower, and kale, along with the traditional white and red cabbage.

The primally goal of NIR examinations are to determine the quality attributes of fresh products and those occurring during storage, such as moisture, SSC, ascorbic acid content, colour, firmness, and freshness. Following various data processing, the best estimation models were developed using PLS or SVR regression [255,256,257].

The protein content of lyophilized broccoli, Brussels sprouts, curly kale, white cabbage, red cabbage, cauliflower, and white kohlrabi was studied by Szigedy et al. [258].

Determining the nitrogen content of the samples is crucial in addition to the quality attributes, as it allows for the monitoring of proper nutrient management and necessary interventions. This ensures the production of an adequate yield and a high-quality product [259].

Successful classification models have been developed based on NIR spectra for determining freshness through colour and for differentiating various Brassica species [233,256].

In the case of red cabbage samples, a high concentration of bioactive components is typical. These include polyphenols (TPC) and anthocyanins (TAC), as well as the antioxidant capacity associated with these compounds.

Antioxidant capacity can be measured using various methods, including ORAC (oxygen radical absorbance capacity), TEAC (Trolox equivalent antioxidant capacity) and DPPH (α, α-diphenyl-β-picrylhydrazyl). Caramês et al. [260] and de Olivera et al. [261] carried out a successful model development using near- and mid-infrared technology. This is also very important because the determination of antioxidant capacity using different methods expresses the antioxidant capacity based on different properties, so the results obtained by different methods are not comparable.

The purple Chinese kale has an extremely high concentration of anthocyanidins, which have notable physiological effects. UHPLC-UV measurements confirmed that cyanidins are present at the highest concentration among the anthocyanidins when compared to other varieties.

Classical methods for anthocyanidin determination are time-consuming both in terms of sample preparation and chromatographic determination. Successful NIR method development has proved to be highly effective not only in quality control but also in vegetable cultivation [262].

Glucosinolates, which are secondary metabolites found in nearly all plants of the *Brassicales* order, make the determination of their concentration in brassicas an important matter. A spectral reflectance technique was developed which is used to quantify the functional components and can be characterized by appropriate chemometric qualification, which replace the chemical-intensive and lengthy classical methods [263,264,265]. 

An estimation function was developed for the quantitative measurement of the residues the pesticides such as profenofos [266], avermectin, dichlorvos, and chlorothalonil [267] using kale, cabbage, and cauliflower as samples. Different chemometric data as well as processing and prediction procedures were compared (Table 8).

**Table 8:** Overview of NIR Results for Brassica (*Brassicaceae*).

Investigated Parameter		Concentration Range	Chemometrics Data			Ref.
			Pre-Treatment, Regression	R^2^	Root Mean Square Error	
Moisture, %		93.35–95.82	2nd der., PLS	0.74	0.25	[255]
Soluble solids content—SSC; °Brix		3.45–5.53	2nd der., PLS	0.64	0.22	
Protein		12.9–32.5	MSC + 1st der., PLS	0.988	0.76	[258]
Ascorbic acid, mg/g		3.8–10.8	2nd der., PLS	0.38	1.3	[256]
		29–68	MSC, PLS	0.95	3.19	[255]
Weight loss rate		0.5–18	SNV, PLS	0.96	1.432	[256]
Surface colour—L*, mg/100 g		64–74	MSC, SVR	0.82	2.013	
Firmness		13–26	autoscale, SVR	0.60	2.453	
Freshness		A; weight loss rate < 30%, L* > 71	SVC	Accuracy A; 93.3%		[256]
		B; 30% ≤ weight loss rate < 50%, 68 < L* ≤ 71		B; 86.6%		
		C; weight loss rate ≥ 51%, L* ≤ 68		C; 86.6%		
		mean 73,777 °C·min	SWSR, PLSR	0.753	22,651	[257]
Classification		Three species	SGS, PLS-DA	SEN 100%, SPEC 95.7%, AC 93.6%		[233]
			SGS, iPLS-DA	SEN 100%, SPEC 97%, AC 94.9%		
N content; g/kg		15.4–48.4	SMLR	0.726–0.846	3.71–4.4	[259]
			PLS		3.84–4.31	
TAC; mg/g		3.04–7.41	1st der., MSC, MC, PLS	0.85	0.47	[260]
TPC; GAEq/g		3.87–6.97	1st der., MSC, MC, PLS	0.78	0.41	
mg GAE/L		101.32–595.72	PLS-OPS, PLS-GA	0.99	10.74	[261]
Antioxidant capacity						
μmol trolox/g	ORAC	434.11–1741.18	1st der., MSC, MC, PLS	0.87	116.34	[260]
μmol trolox/g	TEAC	3.79–6.46		0.85	0.29	
μm trolox/100 g	DPPH	91.01–209.85		0.80	11.47	
μmol trolox/mL	DPPH	0.85–4.79	PLS-OPS,	0.99	0.22	[261]
μmol trolox/mL	ABTS	0.70–5.75	PLS-GA	0.99	0.12	
Cyanidin; μg/g		93.5–12,802.4	DT + 1st der., PLS	0.941	684.969	[262]
		0.02–217.56	RS, PLS	0.56	60.37	[268]
Malvidin; μg/g		0.07–11.82	RS, PLS	0.91	1.04	
Pellargonidin; μg/g		0.02–0.25	RS, PLS	0.74	0.03	
Glycosinolates (total);		7.46–46.50 μg/cm^2^	Exp(Ref), SMLR	0.39	8.067	[264]
Total aliphatic glucosinolates		0–220.94 μmol/g	n.i.	0.9	15.11	[265]
Total indolic glucosinolates, μmol/g		0–30.83	n.i.	0.97	2.35	
Glucoraphanin, μg/cm^2^		1.22–16.02	Ref^2^; 1/Ref, SMLR	0.946	1.12	[264]
4-methoxyglucobrassicin, μg/cm^2^		1.63–7.57	$\sqrt{Ref}$; Exp(Ref), SMLR	0.892	0.646	
μmol/g		0–23.58	SNV-DT, MPLS	0.96	1.82	[265]
μmol/g		0.02–2.58	SNV-DT, SMLR	0.84	0.24	[268]
Neoglucobrassicin, μg/cm^2^		0.28–4.96	1/R; Exp(R), SMLR	0.893	0.386	[264]
μmol/g		0.03–1.56	Ln(Ref), SMLR	0.87	0.11	[268]
Sinigrin, μmol/g		0.03–1.56	Ln(Ref), SMLR	0.86	1.32	
μmol/g		0–132.44	SNV-DT, MPLS	0.99	6.39	[265]
Gluconapin, μmol/g		0.13–1.69	$\sqrt{Ref}$/Exp(Ref)/1/Ref /Ln(Ref), SMLR	0.89	0.12	[268]
μmol/g		0–171.47	SNV-DT, MPLS	0.95	9.06	[265]
Glucobrassicin, μmol/g		0.05–16.77	1/Ref or Ln(Ref), SMLR	0.92	0.88	[268]
Glucoalyssin, μmol/g		0–2.87	SNV-DT, MPLS	0.92	0.34	[265]
Glucoiberin, μmol/g		0–13.18	SNV-DT, MPLS	0.98	2.4	
Pesticide residues; mg/kg						
Profenofos in Chinese kale,		0.60–106.28	SNV + 1st der., PLS	0.97	5.25	[266]
Profenofos in cabbage		0.53–105.36	1st der., PLS	0.88	11.00	
Avermectin		0.25–2.0	RS, RC; LV-SVM	AC 98.33%; PRE 98.46%		[267]
Dichlorvos		0.25–2.0	RS, PLS-DA	AC 98.33%; PRE 95.26%		
Chlorothalonil		0.25–2.0	RS, CARS, PLS-DA	AC 93.33%; PRE 93.57%		
Chlorpyrifos		0.011–2.184	MN, PLS-DA,	AC 100%; PRE 99%		[269]
			RS or MN/SNV-DT/MSC, SVM,	AC 100%; PRE 100%		
			RS, PC-ANN	AC 100%; PRE 100%		
Bacterial contamination						
for thestomacher solution, og CFU/g		2.85–7.08	PLS	0.95	0.46	[270]
for the washing solution, og CFU/g			PLS	0.92	0.44	

#### 5.4.3. Leaf Vegetables 

##### Spinach (Spinacia)

Green colour, texture, and dry matter content are important indicators in assessing the freshness and quality of spinach. Modified partial least squares regression models based on NIR spectra of whole spinach leaves were developed to assess these characteristics, including colour (a* and b* values), texture (measuring maximum breaking strength, toughness, stiffness, and displacement), and dry matter content. The calibration model of the dry matter content was suitable for the quantitative evaluation, the texture parameter models were suitable for screening, while in the case of the colour-related parameters, the models allowed a rough screening of the test samples. This method can be a useful tool for on-site analysis, aiding in the optimization of fertilization and irrigation, as well as assessing quality at the time of harvest [271].

NIR models have been developed for on-site quality assessment in the field, during harvest and storage, and for an online analysis of the processing chain. The models were used to predict crop texture, dry matter, soluble solids content, ascorbic acid content, and safety parameters, such as nitrate content. The further development of these methods has allowed real-time monitoring of the spinach plant growth process. The PLS-DA method was employed to ascertain if a pattern of spinach-usage (fresh, quick-frozen) could be detected based on spectra and nitrate content. [77,272,273,274]. 

The use of the non-linear regression method (LOCAL) for the determination of nitrate concentration led to a model with more favourable statistical properties [275].

The microbiological spoilage (*Pseudomonas*) of baby spinach through various non-destructive approaches, such as the NIR technique, have been investigated. The data were analyzed using PLS and SVR algorithms. The findings suggest that with the appropriate sensor and algorithm, this method could be universally applied to all food products [276] (Table 9).

##### Lettuce (*Lactuca*)

A non-destructive measurement method based on Vis-NIR spectra has been developed for the determination of chlorophyll, carotenoid, and anthocyanin in three different varieties of lettuce (*Lactuca sativa* L.): crystal—green crinkled leaves, Regina 2000 plain green leaves, and Mimosa—slightly red, crinkled leaves [277].

Boros et al. [278] investigated the nitrate content of five varieties of lettuce applied FT-NIR technik (batavia, butterhead, lollo, and oak leaf; (both red and green coloured) during autumn and spring harvesting, as well as under open field and greenhouse cultivation conditions. 

Wu et al. employed a variable selection and GA-LDA to develop a classification model that effectively differentiates between organic and non-organic vegetables using the Vis-NIR spectrum data from the stems and leaves of leafy vegetables (water spinach, amaranth, lettuce, and pakchoi) [279].

A method was developed for the non-destructive and accurate qualitative detection of pesticide residues in vegetables, specifically tested on lettuce leaf samples for fen valerate and chlorpyrifos residues. Following data preparation and variable selection, a classification was performed based on the transmission spectra [280].

Biological contaminants in fresh-cut lettuce, like worms, have been detected using multispectral imaging algorithms combined with Vis-NIR and NIR techniques. Following variable selection, the worm detection algorithms for both Vis-NIR and NIR imaging demonstrated high prediction accuracy [281]. 

When examining lettuce samples, the identification of not only bacterial infections (mainly *Escherichia coli*) but also fungal infections (*Aspergillus niger, Fusarium oxysporum* and *Alternaria alternata*) is of particular importance. Different chemometric classification methods, including SIMCA, SNV, PLS-DA, PCA, and HCA, were used to analyze and distinguish between safe and unsafe samples in the different microbial loads on the spectra [282]. Fungal infections mainly occur in lettuce grown in aquaponic systems, where chemical control of fungal pests is not possible, as it can be fatal to fish. The tested pathogens had a statistically significant effect on the water content of lettuce leaves and the water band index (WBI). The distinct spectral changes induced by each pathogen might potentially provide a way to not only detect infection but also identify the type of pathogen involved. Plant senescence reflectance index and WBI were significantly different for plants infected by *A. niger* and *A. alternata*, and could serve as key indicators for these specific pathogens. Among Vis-NIR reflectance spectra and vegetative indices, WBI proved to be the most reliable in distinguishing between infected and healthy plants [283] (Table 9).

#### 5.4.4. Root Vegetables

Artificial Neural Networks (ANNs) have been utilized to forecast the content of completely dissolved solids, polyphenols, and antioxidant capacity in root vegetables, such as celery, fennel, carrots, yellow carrots, purple carrots, and parsley. These assessments were conducted on samples that were fresh, conventionally dried at 50 °C and 70 °C, as well as freeze-dried. Extractions were carried out using two distinct solvents [284].

A non-destructive method has been developed to determine the reducing sugar and protein content of sweet potatoes. A stepwise regression, combined with the regression coefficient (SRRC) method, was used to select optimal wavelengths for optimizing full-band PLS models [285,286]. 

Near-infrared reflectance spectroscopy combined with chemometric is suitable for analyzing and differentiating between powdered, pure, and adulterated samples of purple and white sweet potatoes. In addition to detecting falsification, the total anthocyanin content and antioxidant activity of the samples were evaluated, and the established estimation functions demonstrated a high residual prediction deviation (RPD) (Table 10) [287]. 

#### 5.4.5. Pumpkins (Cucurbitaceae)

A NIR method was developed to determine the β-carotene content in pumpkin flesh, peel, and seed samples, with acetone as the extraction solvent. The highest concentration of β-carotene was found in the peel, followed by the flesh. The β-carotene content in pumpkin seeds could not be detected using the NIR technique [288].

The applicability of Vis-NIR spectroscopy and colour spectroscopy has been investigated to determine the total carotenoid and flavonoid content of three different cucumber varieties. The study examined how varying concentrations of ethephon (0, 150, and 300 ppm) influenced spectral characteristics and pigment prediction accuracy. It was demonstrated that non-destructive measurement techniques, utilizing a colour spectrophotometer and Vis/NIR spectroscopy, yield reliable predictions of total carotenoid and flavonoid content [289]. 

Classification models were developed using the Vis-NIR spectra of zucchini, bitter gourd, squash, cantaloupe, chayote, and cucumber to distinguish between these products. A comparison of various classification algorithms revealed that only one of the zucchini sample was incorrectly classified [290] (Table 11).

**Table 9:** Overview of NIR Results for Leaf vegetables.

Sample	Investigated Parameter	Concentration Range	Chemometrics Data			Ref.
			Pre-Processing, Regression	R^2^	Root Mean Square Error	
Spinach	Dry matter, %	7.35–18.83	SNV, DT, 2nd der., MPLS	0.70	1.58	[271]
		4.10–19.12	SNV, DT, 1st der., 2nd der., MPLS	0.66	1.22	[272]
		6.12–20.34	SNV, DT, 1st der., 2nd der., MPLS	0.68	1.27	[274]
	Texture					
	Maximum puncture force, N	0.37–4.51	SNV, DT, 2nd der., MPLS	0.62	0.83	[271]
		1.03–4.57	SNV, DT, 1st der., 2nd der., MPLS	0.3	0.41	[272]
	Tughness, mJ	0.38–8.73	SNV, DT, 2nd der., MPLS	0.63	1.50	[271]
	Stiffness, N/mm	009–1.03	SNV, DT, 2nd der., MPLS	0.65	0.20	
	Displacement, mm	0.57–6.05	SNV, DT, 2nd der., MPLS	0.50	1.2	
	Colour					
	a*	−17.32–(−10.78)	SNV, DT, 2nd der., MPLS	0.31	1.09	[271]
	b*	13.77–23.02	SNV, DT, 1st der., MPLS	0.13	2.22	
	Soluble solids content—SSC; °Brix	5.6–14.25	SNV, DT, 2nd der., MPLS	0.86	0.59	[77]
		4.10–11.45	SNV, DT, 2nd der., MPLS	0.80	0.67	[272]
		5.8–14.4	SNV, DT, 1st der. 2nd der., PCA, PLS	0.62	1.0	[274]
		5.2–15.2	SNV, DT, 1st der., 2nd der., MPLS	0.68	1.0	[273]
	Ascorbic acid, mg/100 g	157–454	SNV, DT, 1st der., MPLS	0.25	55.19	[77]
	Nitrate content, mg/kg	109–5177	SNV, DT, 2nd der., MPLS	0.41	834.27	
		41–3526	SNV, DT, 1st der., 2nd der., PLS	0.59	725	[274]
		67–3844	SNV, DT, 1st der., MPLS	0.51	567.79	[272]
		70–3875	SNV, DT, 1st der., 2nd der., MPLS	0.62	688	[273]
		41–3845	SNV, DT, MPLS	0.45	920	[275]
		623–3845	SNV, DT, LOCAL	0.60	758	
	Microbiological spoilage, log CFU/g	6.8–9.0	random data partitioning, SVR	0.4	0.6	[276]
Lettuce	Pigments	5.0–8.5	DT, PLS	0.8	0.495	[277]
	Chlorophyll, mg/kg					
	Total carotenoid, mg/kg	0.9–1.8		0.76	0.105	
	Anthocyanins, mg/kg	0.1–4.0		0.89	0.592	
	Nitrate content, mg/kg fresh w.	1200–2750	SNV, MSC, PLS	0.90	99.4	[278]
	Classification	variety types	LDA			
		red and green leaved variants of lollo and oak leaf variety types	LDA	AC 100%; PRE 100%		
		organic and no-organic	SG, LDA	AC 96.4% (leaf); 96.9% (stem)		[279]
			SS/RF/ANOVA, GA-LDA	AC 92.1/84.9/80.5%		
	Potassium, mg/100 g	165–480	1st der., CARS, PLS	0.83	39.7	[291]
	Green leaves (mixed samples)		1st der., RBF-NN	0.86	38.06	
	Petioles (mixed samples)		CARS, PLS	0.71	31.20	
			RBF-NN	0.88	27.63	
	Pesticide residues	n.i.	SGS, SNV, CARS-IRIV-SGS, SNV, GSA-SVM	AC = 98.33%		[280]
	Biological investigations	n.i.	ANOVA, HSI	AC 97% (Vis-NIR), 100% (NIR)		[281]
	Worms					
	*Escherichia coli*		SNV + 2nd der., PLS-DA	0.958	0.257	[282]
		0.1, 0.2, 0.3 mL	SVM	AC = 100%		

**Table 10:** Overview of NIR Results for Root Vegetable and Sweet Potatos.

Sample	Investigated Parameter	Concentration Range	Chemometrics Data			Ref.
			Pre-Treatment, Regression	R^2^	Root Mean Square Error	
Root vegetable	Fresh		Hidden/Output activation function, ANN, MLP			[284]
	Total dissolved solids, mg/L	10–690	Tanh/Exponential, ANN, MLP	0.9101	0.0165	
	Polyphenol content, TPC	3.5–800	Exponential/Logistic, ANN, MLP	0.7864	0.0141	
	Antioxidant capacity, DPPH mmol Trolox/g	0.04–0.33	Tanh/Identity, ANN, MLP	0.7356	0.0234	
	Dried 50 °C		Hidden/Output activation function, ANN, MLP			
	Total dissolved solids, mg/L	5–460	Tanh/Exponential, ANN, MLP	0.7625	0.0262	
	Polyphenol content, TPC	1–29	Exponential/Logistic, ANN, MLP	0.8090	0.0363	
	Antioxidant capacity, DPPH mmol Trolox/g	0.02–0.19	Tanh/Identity, ANN, MLP	0.8409	0.0017	
	Dried 70 °C		Hidden/Output activation function, ANN, MLP			
	Total dissolved solids, mg/L	30–500	Tanh/Exponential, ANN, MLP	0.8141	0.0167	
	Polyphenol content, TPC	1–13.5	Exponential/Logistic, ANN, MLP	0.7772	0.0128	
	Antioxidant capacity, DPPH mmol Trolox/g	0.05–0.15	Tanh/Identity, ANN, MLP	0.8452	0.0029	
	Lyophilized		Hidden/Output activation function, ANN, MLP			
	Total dissolved solids, mg/L	60–620	Tanh/Exponential, ANN, MLP	0.8201	0.0117	
	Polyphenol content, TPC	1–30	Exponential/Logistic, ANN, MLP	0.8457	0.0188	
	Antioxidant capacity, DPPH mmol Trolox/g	0.01–0.27	Tanh/Identity, ANN, MLP	0.8246	0.0143	
Sweet potatoes	Reducing sugar content, %	0.35–3.31	SRRC-KM-PLS	0.952	0.264	[285]
	Protein content, %	2.53–6.87	2nd der., PLSR	0.96	0.29	[286]
	Total anthocyanins	0.449–0.563 (PSP) 0.027–0.084 (WSP)	RBF-PLS	0.985	0.031	[287]
	Total antioxidant activity,		RBF-PLS			
	DPPH, μmol trolox/100 g DW	570.0–585.0 (PSP)		0.975	1.602	
		554.6–562.0 (WSP)				
	ABTS, μmol trolox/100 g DW	2.593–3.108 (PSP)		0.974	0.148	
		0.713–1.195 (WSP)				
	Fe^2+^ chelate., mg EDTA/g DW	3.736–3.891 (PSP)		0.991	0.02	
		3.371–3.446 (WSP)				
	Classification		SPA, kNN,	RR 100%, PR 94.9%		
			SPA, LDA	RR 100%, PR 97.4%		
			kNN, GA-PLS	RR 100%, PR 97.4%		
			LDA, GA-PLS	RR 100%, PR 100%		

**Table 11:** Overview of NIR Results for Pumpkins (*Cucurbitaceae*).

Investigated Parameter	Concentration Range	Chemometrics Data			Ref.
		Regression	R^2^	Root Mean Square Error	
β-Carotene—pumpkin, µg/g		n.i.	AC 92.0–96.0%		[111]
Flesh	289–313				
Peel	376–451				
Seed	n.i.				
Total Carotenoid Content, mg/100 g—cucumber	3.86–410.68	+150 ppm ethephon	0.91	51.27	[289]
Total Flavonoid Content, mg/100 g—cucumber	26.12–349.84	+150 ppm ethephon	0.87	41.67	
Classification	zucchini, bitter gourd, ridge gourd, melon, chayote, and cucumber	SNV, kNN, Bayes, DT, SVM	accuracy rate 99%		[290]

**Table 12:** Overview of NIR Results for Legumes, Soybean.

Sample	Investigated Parameter	Concentration Range	Chemometrics Data			**Ref.**
			Regression	R^2^	Root Mean Square Error	
Legumes	Gross energy, kcal/g	4.149–4.511	1st der., PLS	0.966	0.0248	[292]
	Fatty acids, %	0–63.18	MSC, SNV, MPLS	0.59–0.93	0.08–3.55	
	Mineral content, mg/100 g					[293]
	Mg (ground)	65.77–164.74	DT + 2nd der., MPLS	0.82	63.29	
	Ca (whole/ground)	23.87–123.74	2nd der./2nd der., MPLS	0.98/0.73	145.09/128.4	
	Fe (whole/ground)	6.98–38.07	MSC + 1st der/DT + 2nd der., MPLS	0.67/0.66	15.48/14.68	
Soybean	Moisture, %	8.16–18.10	SNV, PLS	0.80	1.55	[294]
	Ash, %	4.32–6.14		0.63	0.38	
	Lipid, %	12.55–26.96		0.71	1.20	
	Protein, %	31.52–43.48		0.81	1.61	
	Carbohydrate, %	13.34–27.50		0.50	3.71	
	Dietary fibre, %	10.6–19.2	2nd der., PLS	0.80	0.86	[295]
	Total fatty acid, mg/g	40.25–365.03	SNV, DTT, MPLS	0.94	8.76	[296]
	Tocopherol, μg/g	39.57–860.81	raw, MPLS	0.83	35.28	
	Saponin, Abs/g	0.34–2.89	DT, MPLS	0.66	0.33	
	Total flavonoid, Abs/g	0.15–42.30	SNV, DTT, MPLS	0.91	1.27	
	Total isoflavone, μg/g	246.79–2511.65	SNV-DT, MPLS	1	121.58	
	Anthocyanins	0.01–1.97	SNV-DT, MPLS	0.8	0.13	

#### 5.4.6. Legumes (Fabaceae)

Using FT-NIR reflectance spectroscopy, the gross energy content of several legumes (beans—*Phaseolus vulgaris* L, peas—*Pisum sativum* L., lentils—*Lens culinaris* L and soybeans—*Glycine max* L) was studied. An adiabatic bomb calorimeter was used to determine the reference data [292]. 

The plant known as lentils (*Lens culinaris Medicus*) contains a high amount of minerals, including calcium, iron, and magnesium, and a low amount of fat, comprising mostly polyunsaturated fatty acids. Samples of whole and powered brown, green, black, and red lentils were analyzed for their fatty acid composition, fatty acid profile, and mineral content (Mg, Ca, Fe) to develop the NIRS approach. The results show that the fatty acid and mineral content of lentils may be accurately predicted using NIR spectroscopy [293].

Although they are categorized as legumes by nature, soybeans are distinguished from other “traditional″ legumes by their own special qualities. Soybeans have also been the focus of a great deal of research. Researchers have developed correlations with chemometric features suitable for quantitative evaluations through the development of several methodologies. The non-destructive measurement of soybean physiological processes, moisture, ash, carbs, lipids, proteins, dietary fibre, water-soluble proteins, fatty acids, anthocyanins, proanthocyanidins, isoflavones, tocopherol, and saponins may all be carried out using these correlations [294,295,296,297,298]. There are several alternative classification models that can be used to identify cultivars, group beans according to the temperature and length of storage, and distinguish between intact and damaged beans as well as Roundup Ready and regular beans [299,300,301] (Table 12).

### 5.5. Fruit

In the last two decades, the development of fast and non-destructive techniques for fruit quality analysis received considerable emphasis. The most investigated properties include soluble solids content (SSC), titratable acidity (TA), pH and bioactive compounds, as well as freshness, maturity, texture and spoilage, including external and internal effects, for example, the presence of the pathogen. These tests apply to both fresh and stored fruit. Nicolai et al. [302] were among the first to summarize NIR methods for fruit analysis during this period. Recently, several comprehensive reviews [303,304,305,306,307,308] were published on this topic. These reviews provide detailed NIR results for a variety of fruits, including apples, peaches, plums, mangoes, tangerines, kiwis, watermelons, pineapples, and more. The versatility of NIR techniques is proven by the fact that it is not only suitable for determining the previously listed internal properties. It offers a fast and non-destructive method for determining the vitamin C, polyphenol, total carotene, α-, β-, γ-carotene, lutein content of fruits, as well as for testing fruit freshness, ripeness and possible damage. Table 6 offer a detailed summary and comprehensive overview of the NIR methods used for the analysis of fruit samples. Detailed data on fruits are summarized in Table 13, Table 14, Table 15, Table 16, Table 17 and Table 18.

#### 5.5.1. Pome Fruits (*Maloideae*): Apples (*Malus*) and Pears (*Pyrus*)

Apples are among the most consumed fruits globally, and the challenges posed by climate change and human environmental impact underscore the importance of sustaining quality apple production.

The evaluation of apple samples commonly includes measuring the water-soluble solids content (SSC), total acid content (TA), and the SSC/TA ratio to assess ripeness. For pear samples, a hardness test is also conducted. In addition to these intrinsic properties, Grabska et al. [309] summarized the various techniques and approaches used in Vis/NIR testing over the past five years, including authenticity, provenance, identification, counterfeiting, and quality control. 

The models were developed using various variable selection procedures (synergy interval—si, genetic algorithm—GA, random frog—RF, Competitive Adaptive Reweighted Sampling—CARS, Successive Projection Algorithm—SPA) and regression methods (back-propagation artificial neural networks -BP-ANN, PLSR, PCR, MLR). Orthogonal signal correction (OSC) and various derivation steps were used as data processing [307,310,311,312,313,314,315,316,317,318,319,320,321,322,323,324].

When recording spectra from an entire fruit, it is crucial to consider the impact of the spectrum recording’s location and orientation on the model’s accuracy. Compensation models using PLS and LS-SVM were developed to determine the SSC for each measurement position separately (local models) and for the combined dataset of all positions (global position model). Similar methodologies were applied to pear samples, where, besides SSC, firmness was also assessed. For this purpose, models were constructed using PLS, SVM, and Ridge Regression techniques [243,313,318]. 

The models have been created by including as many varieties of pears as possible. Convolutional Neural Network (CNN), PLS, and SVR approaches were used to create single-culture models and multi-species universal models. Multivariate universal models were built using the full spectra and important variables extracted by gradient-weighted class activation mapping (GradCAM) [248,325,326]. 

A notable application of NIR spectroscopy is in estimating α-farnesene and conjugated trienols (CTols; CT258 and CT281) levels. The synthesis and degradation of a-farnesene, e.g., to conjugated trienols (CTols) in apple skin, is closely related to surface scald, a physiological disorder that affects apples during and after storage. Using a PLS regression, a positive correlation was found for α-farnesene and CTols. A global model, independent of CTols type and year, was developed [327].

Data transfer between different spectrometers is an important technical issue, since this way the methods can be made device-independent. The transferability of calibration methods for the most important quality parameters (SSC, TA, pulp density, starch-iodine index, etc.) were investigated using a table-top (XDS) and hand-held ultra-compact spectrometer (MicroNIR) [328]. Others have created a model transfer platform with an internal quality terminal and an interactive cloud data system by developing an autoencoder (AE) neural network model [329].

Classification models were developed for apple samples based on SSC and TA [330] and using colour data to classify the maturity status (unripe, semi-ripe, ripe, or overripe). A hybrid artificial neural network simulated annealing algorithm (ANN-SA) was employed for the classification [331].

The ability to determine the quality of multiple species with a common calibration would be advantageous in certain situations. Based on the similar physical and chemical properties of apples; pears; peaches and apples; as well as pears and persimmons, universal models were developed for fruits’ SSC measurements. The effective wavebands of the three species were selected using moving window partial least squares (MWPLS) regression, there were identified using SPA and MLR model was developed [332,333]. 

During cultivation, harvesting, and storage, fruits are exposed to mechanical damage, microbial infections, and other types of damage that reduce fruit quality, increase the risk of fungal infections, and greatly affect food safety. Therefore, the timely identification of damaged fruits is essential. The classifications of apple samples of different varieties and freshness were investigated using different pattern recognition techniques (principal component analysis—PCA, partial least squares discriminant analysis—PLS-DA). Using variable importance in projection (VIP) variable selection to discriminate between fresh and stored apples, the model for both cultivar and freshness discrimination showed good classification performance [334,335]. 

He et al. [336] and Pandiselvam et al. [334] published a comprehensive summary of work on the detection of fruit damage using non-destructive techniques. 

Bitter pit (BP), sunburn, as well as internal meat and seed browning processes are physiological disorders that develop mainly after harvesting and during storage. The NIR technique, combined with multivariate analysis (PLSR and PLS-DA and iPLS-DA), offers the possibility to predict the occurrence and severity of BP in apples, sun damage symptoms, and seed browning processes [314,317,337,338]. Discrimination models were created by combining different wavelength selection algorithms (CARS, CARS-SPA, MC-UVE and MC-UVE-SPA) and classification (SVM, ELM, kNN and LDA-kNN) methods to detect and predict apple fungal diseases [138]. Others have developed an LS-SVM model based on the transfer component analysis (TCA) method for this problem [339].

Models were developed to predict damage to pear samples caused by insect pests, enabling online, real-time detection [340]. (Table 13).

#### 5.5.2. Stone Fruits (Prunoideae, Anacardioideae)

Stone fruits studies (cherries (*Prunus avium* L.), sour cherries (*Prunus cerasus* L.), peaches (*Prunus persica* L./Batsch), apricots (*Prunus armeniaca* L.), plums (*Prunus domestica* L.), and mangoes (*Mangifera indica*) primarily focus on the quantitative determination of dry matter, soluble solid content (SSC), titratable acidity (TA), pH, phenolic compounds, pectin, and parameters of flesh firmness and colour. 

These basic qualifying parameters also enable the inference of fruit ripeness status, which is crucial for both harvesting and storage. During model development, various data processing procedures (Norris-Williams Smoothing (NWS), Savitzky–Golay Smoothing (SGS), Continuous Wavelet Derivative (CWD), Multivariate Scattering Correction (MSC); and Variable Sorting for Normalization (VSN), SNV, 1st der., 2nd der. and their combination), variable selection methods (Monte Carlo Uninformative Variable Elimination (MCUVE), SPA, CARS, regression coefficients (RC)) and linear and non-linear regression procedures (PLS), kernel partial least squares (KPLS) PCR, Sparse Partial Least Squares Regression (SPLSR), Sparse Partial Robust M Regression (SPRMR), BP-ANN, latent variables analysis (LVA) and independent component analysis (ICA) Feedforward Neural Network (FNN), Linear Deep Belief Network (LDBN, etc.), were applied [319,341,342,343,344,345,346,347,348,349,350,351,352,353]. 

The Kakadu plum (*Terminalia Ferdinandiana*) is an endemic plant in Australia that contains high concentrations of vitamin C, ellagic acid and other bioactive compounds. Due to its special content values also investigated the applicability of NIR spectroscopy to predict the vitamin C content of fruit [354,355].

For some fruits (e.g., mango), linear regression (PLS) was used, while for other fruits (e.g., peaches), non-linear models (LS-SVM) proved to be better [319,356].

In the case of stone fruits, they also tried to develop a universal model for determining SSC. In the case of peaches and nectarines, the model development was successful, but the model was no longer ideal for estimating plum samples [357]. 

Various types of hand-held devices were also developed to directly apply checks on the fruit plantations [351,358,359]. 

Storability, optimal storage conditions, packaging choices, and quality variable monitoring during storage, are also crucial for stone fruits [360,361,362,363]. 

Various classification models (PCA, PLS-DA, KNN, LS-SVM, SVM, LDA, QDA, MDA, CNN, etc.) were developed to distinguish fruits of different maturity states online before harvesting or throughout the processing chain [344,364,365,366,367,368]. 

These models facilitate variety identification [369] and geographical origin determination [370], as well as the detection of potential counterfeiting (e.g., pumpkin for apricot, or pumpkin for peach) [371] and the assessment of physical damage like bruising [334,336,372].

Maturity, harvest, and post-harvest technologies fundamentally determine the relatively short shelf life of plums which is often threatened by *Monilinia* spp. NIRS combined with an electronic tongue is suitable for the detection of *M. fructigena* fungal infection of plums and for the quantitative determination of this fungal contamination [373] (Table 14).

**Table 13:** Overview of NIR Results for Pome Fruits (*Maloideae*).

Sample	Investigated Parameter		Concentration Range	Chemometrics Data				Ref.
				Pre-Processing, Regression		R^2^	Root Mean Square Error	
Apple	Soluble solid content—SSC, %		11.0–14.0	GA		0.911	0.251	[310]
			7.63–18.60	SNV, MSC, CARS-PLS		0.971	0.429	[313]
			8.00–13.60	SGS, 2nd der., CARS-SPA-PLS		0.850	0.443	[318]
			11.0–17.0	SGS, 1st der., SPA, MNLR		0.953	0.754	[323]
			7.8–24.1	SNV, LS-SVM		0.73	0.7	[328]
			9.13–15.66	CARS/PLS		0.9402	0.5079	[329]
	Complex model with pear		11.1–15.2	2nd der., PLS		0.88	0.43	[332]
	Complex model with pear and peach		10.20–15.60	SPA-MWPLS		0.96	0.46	[333]
				MLR		0.96	0.46	
	Titratable acidity—TA, %		0.9–28.4	SNV, LS-SVM		0.68	0.89	[328]
	Firmness, kg/cm^2^		1.5–12.7	SNV, LS-SVM		0.74	0.99	
	Starch-Iodine Index		2–10	SNV, LS-SVM		0.73	0.84	
	Visual ripeness index, VRPI		n.i.	LS-SVR		0.925	0.168	[324]
	RPI		n.i.	PLS		0.777	0.191	
	IQI		n.i.	PLS		0.951	0.291	
	Streif index		n.i.	PLS		0.768	0.082	
	α-farnese, μmol/m^2^		15–1816	NCL, PLS		0.81–0.92	139	[327]
	CTols	CT258	14–502	1st der. BCAP, PLS		0.90; 0.94	59–60	
	CT281		1–450			0.91; 0.78		
	Maturity estimation		SSC, TA, firmness, anthocyanin	SGS, SNV, MSC, SLS, 1st der. 2nd der. PLS, PCR, SMLR, GA-PLS		0.22–0.97	n.i.	[319]
	Internal flesh browning		93 good, 203 defect		PLS	0.83	0.63	[314]
	Sunscald		161 shaded and sun-exposed 100 mild sun damaged	MSC, 2nd der., PLS, iPLS-DA		0.454 0.594-	0.211 0.317	[317]
	Classification		Internal flesh browning		LDA	accuracy >95%		[314]
	Damage		Bruise, Mouldy core Sunburn Internal browning		PLS-DA, SPA-PLS, SELFS, iPLS-DA LDA	accuracy > 90%; 92% R^2^_cv_ = 0.59 accuracy 90%		[336]
			Maturation level—colour		ANN/SA	accuracy 100%		[331]
			Variety; Freshness; Variety, freshness		PCA, VIP, PLS-DA	misclassification 0%; 5.8%; 2.0–3.9%		[335]
			Bitter bit (BP)	269 BP 719 non BP	PLS-DA	accuracy 60–80%		[337]
			Origin	TCA, LS-SVM		accuracy 90.91%		[339]
			Fungal infection	SNV, CARS, SPA, KNN, LDA, LS/SVM, RF		accuracy 98.75%		[138]
Pear	Soluble solid content—SSC, %		8.6–13.8	PLS		0.912	0.662	[311]
			8.6–11.3	SGS, SNV, 1st der., var.sel. PLS		0.58	0.65	[312]
			10.8–14.6	SGS, PLS		0.92	0.41	[315]
			8.6–13.6-	aver. spectra, FWs PLS		0.8611	0.6314	[316]
			9.8–16.8	SGS, MSC, siPLS		0.9657	0.2265	[320]
			13.4–16.9	PCA, Si-GA-PLS,		0.9406	0.165	[321]
			7.20–19.5	SpectraNet–32		0.58	1.08	[322]
			8.2–16.5	SNV, 2nd der., SVM		0.71	0.7	[338]
			11.3–18.5	OSC-PLS		0.85	0.46	[374]
			11.3–18.5	OSC-MLR		0.86	0.46	
	6 cultivars		10.2–25.0	Grad-CAM, SVR, CNN		n.i.	0.33–1.64	[326]
	Complex model with apple		9.2–13.8	2nd der., PLS		0.88	0.43	[332]
	Complex model with apple and peach		10.90–16.90	SPA-MWPLS, MLR		0.96 0.96	0.46 0.46	[333]
	Dry matter		11.4–21.8	SGS, SNV, 1st der., var.sel. PLS		0.65	1.06	[312]
	Firmness		4.2–11.3	PLS		0.854	1.232	[311]
			28.4–127.1	PCA, Si-GA-PLS,		0.9119	5.5003	[321]
			5.0–71.0	SNV, SVM		0.68	7.66	[338]
			15.00–35.86	PLS		0.58–0.845	2.65–3.98	[325]
			1.9–71.2	OSC-PLS		0.68	8.18	[374]
			1.9–71.2	OSC-MLR		0.56	9.28	
	Maturity estimation		SSC, firmness, lignin cont.	SGS, SNV, MSC, OSC, 1st der., 2nd der., siPLS, UVE, MS-UVE-SPA, PLS, MLR, LSSVM, NIPALS		0.61–0.96	n.i.	[319]
	Classification		internal browning	PLS-DA		sensitivity 76%		[338]
			Insect-affect	SGS CBAM-CNN		accuracy 92.71%		[340]

**Table 14:** Overview of NIR Results for Stone Fruits (*Prunoideae, Anacardioideae*).

Sample	Investigated Parameter	Concentration Range	Chemometrics Data			Ref.
			Pre-Processing, Regression	R^2^	Root Mean Square Error	
Peach	Soluble solid content—SSC, %	7.8–14.5	ICA- LS-SVM	0.9537	0.4155	[341]
		11.20–17.0	SNV, PLS	0.849	0.44	[345]
		≈7–23	2nd der., PLS	0.754–0.951	0.566–0.695	[348]
		7.5–13.4	CARS-LDBN	0.9346	0.4409	[353]
		7.30–14.43	PCA, BP-ANN	0.90	0.691	[356]
		13.0–29.7	2nd der., PLS	0.726–0.89	0.612–0.792	[358]
		6.3–17.6	MSC, SNV, PLS	0.45	1.04	[365]
		7.0–16.0	raw spectra, CARS, RC, PLS	0.7747	0.6915	[366]
			PCR	0.7237	0.7576	
	static	10.1–15.2	SPRMR	0.987	0.161	[368]
	online			0.967	0.244	
	Titratable acidity—TA, %	0.53–1.02	PLS	0.4267	0.101	[345]
	pH	4.12–4.88	ICA- LS-SVM	0.9638	0.0497	[341]
		3.69–4.23	PLS	0.521	0.084	[345]
	Dry matter, %	≈7–25	2nd der., PLS	0.786–0.945	0.542–0.734	[348]
		17.67–31.62	2nd der., PLS	0.67–0.725	0.687–0.911	[358]
	Phenols, mg/100 g		SNV, PLS			[345]
	Flesh	16.29–49.71		0.368	1.62	
	Skin	43.81–159.80		0.681	15.7	
	Pectin, μg/g	n.i.	KPLS	0.628	0.069	[349]
	Flesh colour, °hue	68–91	SGS, PLS, MLR	0.92	1.35	[364]
	Firmness (flesh), N	≈1–10.5	2nd der., PLS	0.039–0.656	0.848–1.368	[348]
		8.93–34.10	LOGSIG, MSC, BP-ANN	0.453	3.844	[356]
		4.9–111.7	SGS + 1st der. PLS	0.40	13.2	[365]
	Complex model with pear and peach	6.30–12.00	SPA-MWPLS MLR	0.96 0.96	0.46 0.46	[333]
	Maturity estimation	SSC, pH, TA, firmness	SGS, VN, SNV, MSC, DT, 2nd der. PLS, PB ANN, SVM, LS-SVM	0.73–0.98	n.i.	[319]
Nectarine	Soluble solid content—SSC, %	≈9–23	2nd der., PLS	0.919–0.938	0.589–0.614	[348]
		−17	raw spectra, PLS PCR	0.8473 0.8249	0.77390.7228	[366]
	Dry matter, %	≈9–22	2nd der., PLS	0.928–0.984	0.65–0.7	[348]
	Firmness (flesh)	≈1–11	2nd der., PLS	0–0.496	1.032–1.537	
Apricot	Soluble solid content—SSC, %	≈4.5–20	2nd der., PLS	0.759	1.983	[348]
	Dry matter, %	≈9–20	2nd der., PLS	0.811	1.168	
	Firmness (flesh)	≈1.8–10	2nd der., PLS	0.438	1.379	
Cherry	Soluble solid content—SSC, %	8.7–30.3	PLS LS-SVM	0.97 0.98	1.15 1.27	[347]
		8.7–22.4	SNV + 1st der., PLS	0.897	0.99	[350]
	Titratable acidity—TA, %	0.39–3.04	1st der., PLS	0.938	0.19	
	Total anthocyanin, %	0–164.1	SNV + 1st der., PLS	0.902	16.9	
	Cyanidin, mg/100 g	3.52–80.44	MSC, PLS	0.83	20.58	[343]
	Maturity index (SSC/TA)	3.74–36.14	1st der.	0.939	1.59	[350]
	Dry matter, %	14.70–36.01	SNV + 1st der., PLS	0.939	1.46	
	Classification	maturity degree	QDA	accuracy 98.44%		
		bruise degree	LS-SVM	accuracy 97.3%		[372]
Plum	Soluble solid content—SSC, %	12.43–16.99	PCA, PLS	0.9456	0.456	[363]
		7.90–19.40	SNV + 1st der., PLS	0.965	0.61	[352]
		≈18–24	2nd der., PLS	0.931	0.377	[348]
	powder pure	4.7–6.8 5.3–6.8	2nd der., PLS	0.70 0.72	0.20 0.58	[354]
	Titratable acidity, %	0.07–0.25		0.7702	0.0183	[363]
		0.50–1.70	SNV + 1st der., PLS	0.949	0.07	[352]
	pH	3.42–4.32		0.8299	0.1010	[363]
	Firmness, N	2.15–5.89		0.825	0.532	
		≈1.5–5.5	2nd der., PLS	0.336	0.459	[348]
	Maturity index, MI = SSC/TA	83.52–117.6		0.7663	15.6	[363]
		5.20–38.80	SNV + 1st der., PLS	0.951	1.50	[352]
	Colour (L*)	29.75–53.83		0.867	3.02	[363]
	Dry matter, %	16.32–28.61	SNV + 1st der., PLS	0.882	0.65	[352]
		≈18–23		0.881	0.498	[348]
	Moister, %		2nd der., PLS			[354]
	powder	81.4–86.0		0.71	0.59	
	pure	81.2–86.0		0.86	0.68	
	Vitamin C, mg/100 g	227.4–28,954	2nd der., PLS	0.91	4773	[355]
Plum	Classification			accuracy 100%		[352]
	Mature/immature	TA	MDA, QDA			
		SSC	LDA, MDA, QDA			
		MI	LDA, MDA, QLDA			
	Cultivars		LDA, MDA, QDA			
	*Monilia fructigena*	injury; intact	PCA/LDA	accuracy 91.67% (24 °C); 85.71% (24 °C)		[373]
Mango	Soluble solid content—SSC, %	6.90–21.30	SNV, PLS	0.81	1.07	[351]
		3.8–21.0	SNV + 1st der., PLS	0.87	1.39	[359]
		19.36 ± 1.31	PLS	0.88	0.90	[342]
		7.7–26.3	SNV, 1st der, PLS	0.9	1.2	[344]
	Titratable acidity—TA, %	0.09–4.60	raw spectra, PLS	0.82	0.36	[351]
		0.07–3.03	MSC, 2nd der., PLS	0.74	0.38	[344]
	pH	2.73–6.94	SNV, PLS	0.80	0.45	[351]
	Firmness, N	0.80–56.30	SGS + 1st der., PLS	<0.8	-	
	Dry matter—DM, %	9.68–18.69	SNV, PLS	<0.80	-	
		11.3–22.1	SNV + 1st der., PLS	0.84	0.88	[359]
		15–25	2nd der., MLR	0.92	1.48	[367]
	Firmness, N	4.94–37.10	MSC, 2nd der., PLS	0.72	4.22	[344]
	Textura					[346]
	Average firmness, N/mm	1.19–4.4	raw spectra, PLS	0.70	0.56	
	Toughness, N/mm	20.39–65.69	SLS, PLS	0.53	1.03	
	Rupture force of peel, N	8.47–22.12	raw spectra, PLS	0.75	2.37	
	Rupture distance, mm	4.02–8.75	raw spectra, PLS	0.26	1.25	
	Penetration force in the pulp, N	0.53–3.32	SLS, PLS	0.71	1.98	
	Penetration energy in the pulp, N/mm	2.12–13.24	SLS, PLS	0.71	0.50	
	Maturity estimation	SSC, DM, TA, firmness	SGS, SNVMSC, EMSC, 1st der., 2nd der., PLS, MLR, SVM, ANN, PCR	0.50–0.97	n.i.	[319]
	Ripening index	0.8–6.8	MSC, 2nd der., PLS	0.8	0.8	[344]
	Classification	Maturity based on dry material	KNN/SVM	accuracy 88.2%		[367]
		Ripening status based on SSC	DA	correctly classified: over ripe 81.1% correctly classified: ripe 80% correctly classified: half ripe 59.6% correctly classified: unripe 87.5%		[344]

#### 5.5.3. Soft Fruits

The term “berry fruits″ does not correspond to a classical botanical classification. Based on the shape of the fruits, we classify the strawberries (*Fragaria x ananasa*), currants (*Ribes rubrum* L, *R. nigrum* L.), blackberries (*Rubus caesius* L.), raspberries (*Rubus ideus* L.), blueberries (*Vaccinium ocycoccos* L.), and kiwifruit (*Actinidia chinensis*) into one group.

Strawberries are the most grown berry in the world. Its characteristics are the SSC value and the TA, from which the ripeness can also be inferred; the bright red colour, the characteristic texture, and, finally, its compounds with bioactive, antioxidant properties (vitamin C, anthocyanin and phenolic acid). Given that it is a very fragile fruit, it is advisable to use NIR estimation models for rapid quality control [303,304,308,319,375,376,377,378,379,380]. Research encompassing various genotypes has shown that the spectral data of these genotypes do not differ, suggesting that these models are universally applicable [334,381,382]. 

Rapid monitoring of colour, SSC, TA content, textural changes, and sensory shelf life is crucial for this perishable fruit during refrigerated storage [383,384,385]. 

Strawberries have a brief shelf life and are highly prone to tissue infections, particularly *Botrytis cinerea*. A correlation has been observed between the SSC value of the fruit and its vulnerability to *B. cinerea*, allowing these models to be utilized for screening purposes [382]. 

An NIR estimation model was developed to determine the SSC and anthocyanin content of fresh raspberry samples [386]. 

During the near-infrared spectroscopic analysis of blueberries, non-invasive detection models based on NIR spectroscopy are often limited and unstable due to biological variability factors (variety, season, changes from harvest to sale, etc.). The detection accuracy of the SSC value of packaged and unpackaged products can be improved by using global modelling procedures and appropriate data processing and neural networks [387,388,389]. 

Blueberry leaves are very rich in bioactive compounds. Therefore, special attention has been paid to the NIR estimation of total phenol (TPC), total flavonoid (TFC), and total antioxidant capacity (TAC) [390,391]. Classification models based on NIR spectra were prepared to categorize blueberries by texture (hard and soft) and to detect foreign substances in frozen products [392,393]. 

For kiwi fruit, key selection and pre-harvest grading characteristics include soluble solids content (SSC), flesh firmness (FF), dry matter (DM), and for yellow-fleshed varieties, flesh colour. The NIR technique offers the opportunity to develop accurate models for predicting internal quality characteristics [394,395,396]. 

The balance between soluble solids in the grape berry and titratable acidity and phenolic ripeness, such as anthocyanin concentration, is a key factor in the production of quality wines. NIR estimation models are useful in monitoring both technological maturity parameters and anthocyanin concentration and grape berry composition [397]. The reliability of models that can be applied directly in the vineyard is disturbed by changes in temperature and sunlight (due to their effect on the spectra). 

Developing a global model can correct these influences, so the handheld NIRS device is suitable for outdoor use to assess the quality of the grape cluster [398] (Table 15).

**Table 15:** Overview of NIR Results for Soft Fruits.

Sample	Investigated Parameter	Concentration Range	Chemometrics Data			Ref.
			Regression	R^2^	Root Mean Square Error	
Strawberry	Soluble solid concentration—SSC, %	4.8–9.9	SGS, 1st der., LOCAL PLS	0.83	0.70	[375]
		6.1–11.0	SVM	0.69–0.85	0.98–1.21	[376]
		5.09–7.37	1st der., PLS	0.52	0.7926	[377]
		3.0–9.5	SNV, PLS	0.96	0.291	[379]
	reflectance	7.50–13.70	SGS, SNV, PLS	0.773	0.633	[380]
	transmittance	7.50–13.30	SGS, MSC, PLS	0.906	0.467	
	Titratable acidity, %	0.68–0.96	1st der., PLS	0.3647	0.1140	[377]
		0.387–0.887	SNV, PLS	0.91	0.032	[379]
	Firmness, N	236–826	SGS, 1st der., LOCAL PLS	0.54	0.11	[375]
	external	0.75–1.53	1st der., PLS	0.282	0.3325	[377]
	internal	0.20–0.44		0.1688	0.1075	
	reflectance transmittance	0.97–3.86	SGS, PLS	0.78 0.81	0.43	[378]
	Moisture, %	87.7–92.7	SVM	0.64–0.77	0.89–1.34	[376]
	Brittleness, N	0.81–3.40	SGS, PLS	0.77 0.78	0.33 0.33	[378]
	Total anthocyanin content, mg/kg	803–2355	SNV, PLS	0.9	132.3	[379]
	Chroma colour	33.98–49.11	SNV, PLS	0.93	0.819	
	Lightness	28.25–54.03	SNV, PLS	0.92	1.71	
	Classification	intact, two varieties	LOCAL, PLS-DA	correct class. rate57%, 78%		[383]
		storage shelf-life	CARS-PLS-DA (0.05; 0.1; 0.15 m/s)	95.1; 97.4; 93.3%		[384]
Raspberry	Soluble solid concentration—SSC, %	7.1–16.0	PLS	0.77	0.76	[386]
	Anthocyanin, mg/L	16.0–184.0	SNV, PLS	0.77	12.57	
Blueberry	Soluble solid concentration—SSC, %	8.80–16.90	SGS, MSC, PLS	0.744–0.974	0.383–3.032	[387]
	three cultivars	9.0–16.90	PLS			[388]
	global cultivar			0.874–0.935	0.483–0.639	
	global season			0.83–0.951	0.442–0.494	
	global variation			0.861–0.950	0.48–0.634	
	PE packed	6.9–17.8	BP-PLS	0.947	0.414	[389]
			SNV, UVE-CARS-IRIV, PLS	0.758	0.883	
	Classification	four cultivars	SVM	accuracy: 100, 93.3, 95.6, 100%		[387]
	Hardness	soft-hard	random	accuracy 78%		[393]
	Total phenol concentration, mg/100 g	39.6–272.8	PLS	0.98	6.9	[391]
	Total flavonoid concentration, mg catechin/g	41.2–269.1	PLS	0.97	6.7	
	Total antioxidant activity, mmol Trolox/g	22.6–124.8	PLS	0.98	2.9	
Berry fruit	Total phenol concentration, mg/100 g	39.4–479.5	PLS	0.98	35.48	[390]
	Antioxidant activity, DPPH mmol/100 g	1.7–10.1	PLS	0.99	2.2	
Kiwifruit	Soluble solid concentration—SSC, %	13.18–15.68	SNV, PLS	0.93	0.259	[394]
		4.00–19.70	PLS	0.94	0.97	[396]
	pH	3.45–4.13	SNV, PLS	0.94	0.076	[394]
	Firmness (flesh), N	0.12–10.87	PLS	0.866	9.41	[396]
	Flesh hue, °H	94.96–115.60	PLS	0.843	1.82	
	Dry matter—DM, %	13.526–18.757	GA-siPLS	0.9020	0.5315	[395]
		13.62–21.77	PLS	0.854	0.64	[396]
	Maturity estimation	DM, SSC, TA, Firmness	SNV, MSC, VN, SGS, 2nd der. PLS, LDA, SVMR, LSSVM, MLR, PCR	0.73–0.98	n.i.	[319]
Grape	Soluble solid concentration—SSC, %	13.8–23.6	2nd der., PLS			[398]
	EPO + GLSW corr. for temperature interference			0.90–0.91	0.96–0.98	
	EPO correction for sun			0.98	0.50	
	Maturity estimation	Phenolic comp, TA, pH, colour, BrimA	SGS, SNV, MSC, DT, 1st der., 2nd der., PLS, MPLS, MLR, LS-SVM	0.6–0.982	n.i.	[319]

#### 5.5.4. Citrus Fruits

NIR testing across various citrus fruits, such as lemons (*Citrus* × *limon*), oranges (Citrus sinensis), mandarins (*Citrus reticulata*), limes (*Citrus aurantiifolia*), and grapefruits (*Citrus* × *paradisi*), is aimed at assessing ripeness, like other fruits. The goal is to swiftly and non-destructively determine soluble solid content (SSC), pH, titratable acidity (TA), and the maturity index derived from these measurements [303,304,308,319,334,399,400,401,402].

The peel thickness of citrus fruits can pose challenges during spectral recording. Investigations have been conducted to identify the optimal location for spectral fixation, considering the stem, equator, and navel positions. While peel thickness can interfere with the spectral data collection of the flesh layer, the prediction model’s accuracy and robustness can be enhanced by integrating spectral data from multiple regions. Hence, more focus on the fusion of multi-information sets is warranted to develop a practical model. Citrus fruits with different peel thicknesses are the primary subjects of the NIR penetration capacity analysis. It was discovered that permeability is influenced by the shell’s composition in addition to its thickness. By prolonging the integration period, the penetration potential can be somewhat increased. Compared to long-wave near-infrared light (LWNIR), higher-energy short-wave near-infrared light (SWNIR) penetrates more deeply. Furthermore, SWNIR is a better option for evaluating the fruit’s internal quality because the peel’s absorption peaks are primarily in the LWNIR range [403,404].

The non-destructive method has also been successfully employed to detect surface damage and fungal infections in citrus fruits [336].

Postharvest rind pitting (RP) is a progressive physiological disorder of the rind that affects citrus fruits during postharvest storage, diminishing their external quality. This disorder manifests 3–5 weeks after harvest, complicating its detection during the grading and sorting processes on commercial packing lines. Principal component analysis has effectively differentiated fruits based on canopy position and their susceptibility to rind pitting disorder. Vis/NIR spectroscopy, in conjunction with chemometric analysis, is suggested as an alternative method for clustering fruits according to canopy position, which is beneficial for identifying fruits with a higher risk of RP, as the incidence of RP is greater in fruits from the outer canopy [405] (Table 16).

**Table 16:** Overview of NIR Results for Citrus Fruits.

Sample	Investigated Parameter	Concentration Range	Chemometrics Data			Ref.
			Regression	R^2^	Root Mean Square Error	
Orange	Soluble solid content—SSC, %	6.80–15.30	LOCAL; MPLS	0.81; 0.75	0.80.; 0.97	[400]
	pH	3.01–4.15	LOCAL; MPLS	0.25; 0.15	0.16; 0.18	
	Titratable acidity-TA, %	0.36–1.02	LOCAL; MPLS	0.45; 0.47	0.11; 0.11	
	Maturity index, SSC/TA	8.24–40.03	LOCAL; MPLS	0.65; 0.67	3.56; 3.70	
	BrimA	4.29–13.31	LOCAL; MPLS	0.82; 0.80	0.85; 0.89	
Mandarin	Soluble solid content—SSC, %	9.95–15.65	LOCAL; MPLS	0.57; 0.39	0.71; 0.84	
	pH	2.08–3.80	LOCAL; MPLS	0.74; 0.74	0.11; 0.11	
	Titratable acidity-TA, %	0.68–2.15	LOCAL; MPLS	0.76; 0.65	0.13; 0.18	
	Maturity index, SSC/TA	5.41–17.27	LOCAL; MPLS	0.79; 0.68	1.13; 1.38	
	BrimA	2.93–10.33	LOCAL; MPLS	0.75; 0.68	0.70; 0.79	
Orange, mandarin	Soluble solid content—SSC, %	6.8–15.65	LOCAL; MPLS	0.78; 0.72	0.86; 0.95	
	pH	2.08–4.15	LOCAL; MPLS	0.72; 0.64	0.15; 0.17	
	Titratable acidity-TA, %	0.36–2.15	LOCAL; MPLS	0.84; 0.75	0.14; 0.18	
	Maturity index, SSC/TA	5.41–40.03	LOCAL; MPLS	0.77; 0.72	2.98; 3.52	
	BrimA	2.93–13.31	LOCAL; MPLS	0.78; 0.73	0.84; 0.94	
Citrus species	Soluble solid content—SSC, %	5.2–14.7	Full-ANN	0.823	0.560	[402]
	Stem, Equator, Navel	10.70–16.90	MN, PLS	0.8424	0.5901	[403]
	Equator, Navel	10.80–16.90		0.8507	0.6015	
	Classification	surface damage	LDA	accuracy 97.80%		[336]
		fungal infection	SVM	accuracy > 90.8%		
Lemon	Soluble solid content—SSC, %	6.32–9.71	PLS	0.84	0.42	[401]
	Titratable acidity—TA, %	4.74–7.29	PLS	0.72	0.45	
Grapefruit	Total antioxidant capacity, mgAS/g	n.i.	normalization, PLS	0.71	0.17	[405]
	β-carotene,	n.i.	SNV, PLS	0.99	0.17	
	Total carotene	n.i.	SNV, PLS	0.91	0	
	Chlorophyll-a, μg/g	n.i.	SNV, PLS	0.86	2.69	
	Chlorophyll-b, μg/g	n.i.	SNV, PLS	0.92	0.01	
	Dry matter, %	n.i.	SNV, PLS	0.88	0.01	
	Carbohydrates	n.i.	SNV, PLS			
	sucrose			0.79	0.03	
	glucose			0.88	0.02	
	fructose			0.92	0.03	
	Rind pitting	n.i.	normalization, PLS	0.89	5.21 × 10^−4^	

#### 5.5.5. Pumpkin Fruits (Cucurbitaceae)

Melon (*Cucumis melo* L.) and watermelon (*Citrullus lanatus*), which are part of the cucurbit family, originate from Asia and Africa, respectively (watermelon is considered a vegetable in terms of cultivation technology).

NIR models are basically total soluble solids content (TSS, an indicator of sweetness), acidity (an indicator of sourness), dry matter (sometimes an indicator of maturity), moisture content (an indicator of juiciness), lycopene content [304,319,406,407,408,409] texture properties, e.g., are aimed at a quick and non-destructive determination of strength and toughness [410]. A study was carried out over two years for cut and intact melons. For cantaloupe, the model derived from two years of data for intact samples was used, whereas for watermelon, the model based on a single year’s data gave superior statistical attributes [411]. The possibilities of rapid measurement of water activity and colour changes during the solar drying of melon slices were also investigated. [412]. Due to the fruits’ thick skin, finding the optimal measuring position is crucial. The mesocarp’s TSS is highest around the equator of the fruit and increases towards the seed cavity, while the inner mesocarp’s TSS levels decrease towards both the proximal and distal ends of the fruit [413,414]. 

Although melon rinds are not consumed, the determination of surface pesticide residues is a key task. A one-dimensional convolutional neural network, with a deep feature fusion structure to capture multi-scale spectral information, has a better identification of pesticide residues on the melon surface. The model is suitable for answering the question “Does it contain pesticide residues or not″, but it was not accurate for estimating imidacloprid and pyraclostrobin residues [415] (Table 17).

#### 5.5.6. Tropical Fruits

“Tropical fruits″ primarily include pineapple (*Ananas*), avocado (*Persea americana*), papaya (*Carica papaya* L.), banana (*Musaceae*), passion fruit (*Passiflora edulis*), and pomegranate (*Punica granatum* L.). Numerous summary articles have presented NIR measurement models for these fruits, assessing attributes such as total soluble solids content (TSS), titratable acidity (TA), maturity index (TSS/TA), pH, firmness, dry matter, vitamin C, polyphenols, pigments, starch content, and colour [302,303,305,319,334,416,417,418,419,420].

In the case of pineapple, a well-liked tropical fruit, spectra recorded from the whole fruit and its slices are used to determine SSC and nitrate content, thereby aiding quality control and sorting processes [421,422,423]. Additionally, a NIR model for passion fruit was created to measure soluble solids content (SSC), titratable acidity (TA), ascorbic acid content (ASC), ethanol concentration (EtOH), peel firmness (PF), and pulp percentage (PP) [424].

Determining the optimal harvest maturity for avocados is crucial. Traditionally, this has been carried out by destructively measuring the oil, dry matter, or moisture content of the mesocarp. However, the Vis-NIR model, introduced as a non-destructive alternative [425,426], has changed this approach. Similarly, for pomegranates, a Vis-NIR model using TSS, pH, and hardness as reference values for quality assessment was developed [427], and for papayas, SSC and starch values were used [428]. In addition to chemical and microbiological parameters, a principal component analysis (PCA) was utilized on the second derivative of the spectra to reveal molecular changes during storage. This analysis clearly distinguished between “fresh″ and “old″ samples, and established a stability time that marks the onset of freshness loss at various temperatures [429] (Table 18).

**Table 17:** Overview of NIR Results for Pumpkin Fruits (*Cucurbitaceae*).

Sample	Investigated Parameter	Concentration Range	Chemometrics Data			Ref.
			Regression	R^2^	Root Mean Square Error	
Watermelon	Soluble solid content—SSC, %	5.3–13.7	SNV, PLS	0.707	1.4	[407]
	cut	2.00–11.50	2nd der., MPLS	0.84–0.88	0.61–0.65	[411]
	intact		1st der., PLS	0.72–0.76	1.89–2.05	
	Lycopene, mg/kg	2.65–151.75	SNV, PLS	0.805	16.19	[407]
	β-carotene, mg/kg	0.19–9.39	SNV, PLS	0.737	0.96	
Melon	Soluble solid content—SSC, %		SGS, MSC, CARS, PLS			[408]
	stylar end	5.5–13.9		0.72	0.82	
	equatorial	5.7–13.6		0.53	1.03	
	cut	4.00–14.00	1st der., PLS	0.85	0.49	[411]
	intact		2nd der., PLS	0.65	0.93	
	Calyx	5.70–15.70	Smoothing, PLS	0.89	1.05	[414]
	Equator	5.30–14.85	Smoothing, normalization, PLS	0.91	0.86	
	Stem	5.10–13.15	Smoothing, normalization, PLS	0.87	0.95	
	Calyx	5.70–15.70	Smoothing, PLS	0.93	0.85	
	full spectra		MC-UVE-SPA, LS-SVM or CARS LV-SVM	0.91	0.96	
	Variable selection		MC-UVE-SPA, MLR	0.91	0.95	
	Texture—using intact fruit spectra					[410]
	Initial firmness, N/mm	0.22–11.17	MSC, PLS	0.387	2.13	
	Ruprure force, N	1.05–18.05	Min-max normalization, PLS	0.850	1.70	
	Average firmness, N/mm	0.22–8.82	SNV, 1st der., PLS	0.502	1.55	
	Rupture distance, mm	0.31–9.17	Min-max normalization, PLS	0.561	1.52	
	Toughness, N/mm	0.18–36.28	SLS, PLS	0.674	3.85	
	Average penetrating force, N	2.59–18.77	Constant offset elimination, PLS	0.845	1.59	
	Penetrating energy, N/mm	446.61–336.46	2nd der., PLS	0.749	35.40	
	Moisture, %	89.4 → 17.8	SNV, 2nd der., PLS	0.99	2.49	[412]
	Water activity	0.9994 → 0.4666	SNV, 2nd der., PLS	0.97	0.03	
	Colour		SNV, 2nd der., PLS			
	a*	7.92 → 22.48		0.91	1.13	
	b*	24.61 → 48.78		0.86	2.49	
	C*	25.81 → 53.81		0.87	2.52	
	Browning index	64 → 150	SNV, 2nd der., PLS	0.86	11.00	
	Classification	pesticide residue	1D-CNN	accuracy 91.67–95% (validation) accuracy 90.00–95.85 (test set)		[415]

**Table 18:** Overview of NIR Results for Tropical Fruits.

Sample	Investigated Parameter	Concentration Range	Chemometrics Data			Ref.
			Pre-Processing, Regression	R^2^	Root Mean Square Error	
Pineapple	Soluble solid content—SSC, %	11.90–18.60	MSC, PLS	0.854	0.842	[422]
		7.0–18.5	MSC, PLS	0.88	1.04	[423]
	Maturity index (colour based)	0.55–1.20	1st der., PLS	0.97	0.034	[423]
	Nitrate level, mg/kg					[421]
	Individual spectrum model	3.71–51.07	MSC, SNV, 1st der., PLS	<0.90	n.i.	
	Average spectrum model	6.56–28.27	1st der., PLS	0.94	2.08	
	Classification	organic and inorganic fruits	MSC, kNN or MSC, LDA	accuracy 100%		[422]
Passion fruit	Soluble solid content—SSC, %	13.70–20.07	2nd der., PLS	0.908	0.76	[424]
	Titratable acidity—TA, %	0.38–2.85	2nd der., PLS	0.68	0.26	
	Ascorbic acid, mg/100 g	14.20–27.67	2nd der., PLS	0.663	2.46	
	EtOH, g/L	0.60–2.94	2nd der., PLS	0.849	0.25	
	Peal firmness, N	4.85–22.76	2nd der., PLS	0.829	2.38	
	Pulp percent, %	43.55–82.31	2nd der., PLS	0.883	3.76	
Avocado	Dry matter, %	19.4–34.2	PLS	0.75–0.89	1.14–2.60	[425]
		14.15–39.59	PLS	0.95	2.49	[426]
	Moisture content, %	65.8–80.6	PLS	0.84–0.92	1.14%	[425]
		63.89–85.85	PLS	0.95	2.49	[426]
Bananas	Soluble solid content—SSC, %	n.i.	PLS	0.99	0.80	[303]
		6.47–24.10	PLS	0.81	3.91	[420]
	mesocarp	11.07 ± 7.79	PLS	0.97	1.77	[342]
	ripe, over ripe	18.62 ± 2.06		0.79	0.54	
	pH	5.23–6.31	PLS	0.83	n.i.	[303]
		n.i.	PLS	0.69	0.36	[420]
	Dry matter, %	n.i.	MLR	0.83	n.i.	[303]
	mesocarp	24.60 ± 1.53	PLS	0.88	0.73	[342]
	ripe, overripe	24.53 ± 1.58		0.88	0.54	
Pomegranate	Soluble solid content—SSC, %	18.42–19.2	SNV, median filter, 1st der., MC, PLS	0.94	0.21	[427]
	pH	3.42–3.65	SNV, median filter, 2nd der., MC, PLS	0.86	0.069	
	Firmness, N	38.5–41.97	SNV, median filter, 1st der., MC, PLS	0.94	0.68	
Papaya	Soluble solid content—SSC, %	3.47–8.9	MSC, PLS	0.9	0.12	[428]
	Starch, mg/g	0.3–5.31	MSC, 1st der., PLS	0.9	0.12	

### 5.6. Luxury Items

Coffee, tea and chocolate are sought-after luxury items. They do not belong in our regular diet; thus their intake is insignificant. When ingested in sufficient amounts, the alkaloids and polyphenol chemicals included in them also have a positive physiological impact.

It is no accident that most research on luxury products concentrate on identifying these vital physiological components. Thanks to the evolution of the instrumental analytical methods employed as a reference, today, e.g., not only can we establish the total polyphenol content, but we can also identify them individually and estimate their number using the NIR spectroscopic approach. 

Since these are expensive foods, it is crucial to identify their origin (e.g., Arabica or Robusta in the case of coffee), their location (varying quality depending on geological origin), and any potential adulteration.

Most publications from 2004 to 2014, as Table 19, Table 20, Table 21 and Table 22 illustrate, focused on the analysis of different luxury goods.

#### 5.6.1. Tea

Teas, derived from *Camellia sinensis*, are complex products whose quality and sensory attributes are influenced by a variety of factors such as geographical origin, processing methods, and storage conditions. Generally, there are huge amounts of types and brands of teas in the market, and the price and quality grading are distributed in a large range [430]. After being plucked, the fresh tea leaves are sent immediately to tea factories for manufacturing. Due to the different ways of processing, especially the extent of oxidation, tea is usually divided into three basic types: green tea, oolong tea, and black tea. Alternatively, with the combination of the ways of processing and the characteristic quality of manufactured tea, tea is classified into six types: green tea, yellow tea, dark tea (containing brick tea and pu-erh tea), white tea, oolong tea, and black tea [431].

A wide range of analytical methods and standards are available for testing the quality parameters of tea. The importance of the measurements lies in the fact that the above-mentioned factors determine the price of tea to a large extent. Therefore, NIR spectroscopy has proven highly effective in assessing key quality parameters, including moisture content, polyphenol concentration, caffeine content, and the levels of other bioactive compounds, such as catechins and theanine. In addition to conventional desktop instruments, several studies have examined the applicability of handheld NIR spectrometers.

Based on the reviewed publications, the most frequently studied types of tea were green and black teas. Numerous studies focused on the classification of teas, with a particular emphasis on distinguishing tea types or their geographical origin. For pre-processing the spectral data, the most commonly used technique was SNV correction. Both linear and non-linear mathematical methods were applied for modelling, including PLS-DA, SVM, SIMCA, kNN, and ANN. In all cases, the accuracy of the models exceeded 83%.

Another key area of study was the characterization of teas in different oxidation states through their chemical composition. The most important parameters in tea characterization were sensory properties, caffeine content, total polyphenol content, various catechins, pigments (e.g., thearubigins, theaflavins), and theanine concentration. For quantitative estimation, a variety of chemometric methods were employed, such as PLSR, SVMR, MLR, and PCR. During method development, variable selection techniques were often used, including GA, SPA, CARS, LTSA, RF, ACO, IVSO, FPA, IRIV, IVISSA, and BOSS (Table 19).

**Table 19:** Overview of NIR Results for Tea.

Sample	Investigated Parameter		Concentration Range	Chemometrics Data			References
				Pre-Treatment, Regression	R^2^	Root Mean Square Error	
Green tea	TAC, μmol Trolox/25 μg leaf		14.53–35.79	PCR	0.76	1.81	[432]
	Ranking		0–58	SNV, MC, 2nd der., PLS	0.99	3.05	[433]
	Sensory		61–94	SNV, BP AdaBoost	0.77	6.0807	[434]
	Classification of origin			1st der., PLS	100.0%		[435]
	Moisture, %		6–76.75	Z-score, PCA-SVM	0.97	0.046	[436]
Whole leaves	Caffeine, μg/kg		n.i.	1st der., PLS	0.96	0.18	[437]
	Catechin, μg/kg		0.14–1.08	GA-PLS	0.98	0.99	[438]
			n.i.	SPA-PLS	0.931	1.002	[439]
	CG		n.i.	SPA-PLS	0.892	0.487	
	EC, μg/kg		n.i.	SNV, PLS	0.61	0.071	[437]
			n.i.	SPA-MLR	0.955	1.033	[439]
			0.15–0.39	siPLS	0.91	0.78	[438]
	EGCG, μg/kg		n.i.	1st der., PLS	0.85	0.54	[437]
			7.65–14.30	siPLS	0.97	0.85	[438]
			n.i.	SPA-PLS	0.964	2.143	[439]
	ECG, μg/kg		1.76–3.78	siPLS	0.96	0.78	[438]
			n.i.	SPA-PLS	0.989	0.664	[439]
	Gallocatechin		n.i.	SPA-MLR	0.985	0.199	[439]
	GCG		n.i.	SPA-MLR	0.890	0.302	[439]
	Theanine, μg/kg		0.86–2.80	SA-PLS	0.93	0.8	[438]
	AC, Trolox eq		n.i.	1st der., PLS	0.92	88	[437]
	AC, %		65.07–80.59	SA-PLS	0.80	0.72	[438]
	Discrimination of grade			MSC, MC, siPLS	93%		[440]
	EGC		n.i.	SPA-PLS	0.981	0.658	[439]
	Gallic acid		n.i.	SPA-PLS	0.894	0.094	[439]
Green powder	Caffeine,	%	n.i.	2nd der., PLS	0.97	0.19	[437]
	m/g		4.6–35.9	weighted MSC, mPLS	0.97	1.538	[441]
	%		2.2611–3.7616	SNV, PLS	0.97	0.08	[442]
	mg/g		16.09–55.31	SNV, SVM	0.95	2.4	[443]
	Catechin,	%	0.1–2.8	MSC, mPLS	0.91	0.25	[441]
	mg/g		92.05–194.13	SNV, SVM	0.97	7.23	[443]
	Gallic acid, mg/g		0.02–0.89	weighted MSC, mPLS	0.85	0.045	[441]
	Gallocatechin, mg/g		0.3–2.9	DT, mPLS	0.78	0.374	
	EC, mg/g		2.0–15.2	SNV, DT, mPLS	0.95	0.848	
Green powder	EGC, mg/g		1.0–59.8	weighted MSC, mPLS	0.95	3.333	
	EGCG,	%	7.34–14.30	DT, SNV, GA-siPLS	0.96	0.35	[444]
	mg/g		5.6–143.9	mPLS	0.97	4.313	[441]
	ECG, mg/g		1.9–26.6	SNV, DT, mPLS	0.94	1.419	[441]
	EGC-3-(3′-O-methyl) gallate, mg/g		0.07–2.60	SNV, mPLS	0.58	0.256	[441]
	GCG, mg/g		0.08–3.28	mPLS	0.85	0.2	[441]
	Total catechins, mg/g		22.1–206.8	weighted MSC, mPLS	0.97	9.463	[441]
	Total polyphenol content,%		19.1543–30.2329	2nd der., PLS	0.93	1.11	[442]
			14.93–25.46	SNV, siPLS	0.96	0.7327	[445]
	EGC,%		2.126–5.428	MC, PLS	0.99	13.65	[446]
	EC,%		0.131–0.397	MC, PLS	0.96	1.74	
	EGCG,%		7.340–14.088	SNV, PLS	0.98	38.39	
	ECG,%		1.764–3.784	SNV, PLS	0.98	11.76	
	AC, Trolox eq.		n.i.	DT, PLS	0.88	124	[437]
	Antioxidant activity		0.442–0.806	min/max norm., SVM	0.97	0.02	[447]
	Lutein,%		0.285–1.063	DT, SPA-MLR	0.98	0.003	[440]
	Chlorophyll-a,%		0.075–1.041	MSC, SPA-MLR	0.97	0.005	
	Chlorophyll-b,%		0.012–0.536	1st and 2md der.,	0.99	0.001	
	Pheophytin a,%		0.131–0.343	N, SPA-MLR	0.92	0.001	
	Pheophytin b,%		0.299–1.205	SPA-MLR	0.96	0.006	
	ß-carotene,%		0.119–0.879	1st and 2nd der., SPA-MLR	0.97	0.004	
	Sensory score		69.5–90.0	LTSA-RVM	0.96	1.461	[448]
	Physical quality		19–25	MSC, 1st der., PLS	0.90	0.496	[449]
	Total cup quality		77–83	VN, 1st der., PLS	0.90	0.504	
	Colour		7–10	MSC, PLS	0.91	0.217	
	Aroma		20–25	VN, 1st der., PLS	0.90	0.371	
	Taste quality		19–29	MSC, 2nd der., PLS	0.89	0.744	
	Leaf		7–10	MSC, 1st der., PLS	0.90	0.214	
	Bitterness		1–5	VN, 1st der., PLS	0.91	0.306	
	Flavour		1–5	MSC, 1st der., PLS	0.95	0.297	
	Body		1–5	MSC, PLS	0.96	0.261	
	Overall quality		1–5	MSC, 1st der., PLS	0.92	0.376	
	Classification	Grade		SNV, MC, SOLPP	100%		[450]
	Varieties			SNV, MC, SOLPP	100%		
	Origin			SNV, MC, SOLPP	100%		
Green powder	Adulteration			SNV, SVM	97.47%		[451]
	with sugar, glutinous rice						
	with sugar, %		0.2–40	SNV, IRIV-SVM	0.998	0.67	
	with glutinous rice, %		0.2–15	SNV, SVM	0.97	1.16	
Powder and granules	Caffeine, mg/100 mL		ca. 15–95	2nd der., PLS	1.00	1.81	[452]
Roasted	Classification of origin			SNV, SVM	100%		[453]
Infusion	Polyphenols: amino acids ratio		2.724–4.575	SNV, PLS	0.87	0.316	[454]
Chinese green	Classification of grade			SNV, PLS-DA	>92.4%		[455]
Instant	Caffeine, %		1.95–9.89	SNV, PLS	0.99	0.165	[456]
	Catechin, %		3.51–23.4	SNV, GA-PLS	0.96	1.13	
	EGC, %		2.41–9.94	SNV, PLS	0.88	0.654	
	EGCG, %		0.24–9.43	SNV, GA-PLS	0.95	0.578	
	EC, %		0.64–3.29	SNV, PLS	0.96	0.533	
	ECG, %		0.06–5.92	SNV, PLS	0.94	0.349	
Black tea	Moisture, %		n.i.	SNV, PCA, SNV-PCA	0.99	0.00953	[457]
			2.8–5.0	SGS, Normalization, PLS	0.89	0.19	[458]
	Colour		10.0–19.0	SGS, Normalization, PCR	0.84	0.81	
	Body		11.0–19.0	SGS, SNV, PLS	0.97	0.29	
	Quality		7.0–19.0	SG, MSC, PLS	0.85	0.9	
	Appearance		6.0–19.0	SGS, Normalization, PCR	0.93	0.62	
	Density		127.0–550.0	SNV, PLS	0.89	29.66	
	Water extract		27.6–42.0	SGS, Normalization, PCR	0.81	1.39	
	Cellulose		10.0–18.7	SGS, SNV, PLS	0.66	1.07	
	Catechin, mg/g		5.97–7.46	SNV, CARS-LSSVMR	0.98	0.0024	[459]
	CG, mg/g		0.03–0.05	SPA-LSSVMR	1.00	0.0005	
	EC, mg/g		0.77–5.61	MSC, CARS-LSSVMR	0.99	0.001	
	ECG, mg/g		1.77–2.09	SNV, CARS-LSSVMR	0.98	0.0021	
	EGC, mg/g		0.80–1.18	SNV, CARS-LSSVMR	0.98	0.004	
	EGCG, mg/g		2.55–4.00	MSC, SPA-LSSVMR	0.99	0.0009	
	Gallocatechin, mg/g		7.64–18.2	SNV, CARS-LSSVMR	0.99	0.0006	
	GCG, mg/g		1.17–1.63	SNV, CARS-LSSVMR	1.00	0.0002	
	Ash, %		5.84–7.95	IVISSA-PLS	0.95	0.0192	[460]
Black powder	Caffeine,	%	2.13–4.28	MSC, PLS	0.96	0.16	[461]
	mg/g		ca. 0.5–5	SNV, BP_AdaBoost	0.94	0.21	[462]
	mg/g		0.98–3.55	biPLS	0.92	0.209	[463]
			20.65–56.67	SNV, SVM	0.93	2.51	[443]
	Catechins, mg/g		48.33–156.29	SNV, SVM	0.97	8.4	[443]
	EGCG, mg/g		0.78–19.62	CARS-PLS	0.94	1.74	[464]
	Total catechins, mg/g		ca. 0–8	SNV, BP_AdaBoost	0.72	0.95	[462]
	Water extracts,	%	22.63–49.50	min/max norm., PLS	0.96	0.685	[461]
	mg/g		ca. 20–46	SNV, BP_AdaBoost	0.91	1.73	[462]
	mg/g		26.31–42.09	GA-PLS	0.88	1.47	[463]
	Free amino acids, %		0.52–3.69	SNV, PLS	0.93	0.273	[461]
	TPC,	%	4.21–20.52	min/max norm., PLS	0.95	0.594	[461]
	mg/g		ca. 2–20	SNV, BP_AdaBoost,	0.71	2.35%	[462]
	Colour Sensory score		5.5–9.5	GA-BP-ANN	0.86	0.461	[465]
	Taste quality		1–10	SNV, BP_AdaBoost	0.85	0.64	[462]
	Free amino acids, mg/g		ca. 2.5–6	SNV, BP_AdaBoost	0.89	0.36	[462]
			2.87–5.56	GA-PLS	0.95	0.214	[463]
	Theaflavin-3-gallate, mg/g		ca. 0–1	SNV, BP_AdaBoost	0.72	0.18	[462]
	Theaflavin-3′-gallate, mg/g		ca. 0–0.6	SNV, BP_AdaBoost	0.81	0.08	[462]
	Theaflavins, mg/g		ca. 0–2.5	SNV, BP_AdaBoost	0.77	0.34	[462]
			0.09–1.91	biPLS	0.92	0.162	[463]
	Bitterness		1.83–7.00	CARS-MLR	0.94	0.5058	[464]
	Astringency		1.57–6	CARS-PLS	0.91	0.541	
	Caffeine, mg/g		16.60–57.92	CARS-PLS	0.95	3.13	
	Classification	Origin		SNV, kNN	93.30%		[466]
	Quality categories			SG, SNV, IGA-PSO	95.28%		[467]
Congou black	Theaflavins: thearubigins ratio		0.090–0.156	SNV, BP_AdaBoost	0.89	0.0044	[468]
Darjeeling black	Classification, authentication			SNV + 2nd der., PLS-DA	95.45%	4.55	[469]
Black infusion	Caffeine,	%	1.35–2.39	VN, PLS	0.97	0.08	[470]
Black and green—powder	mg/g		16.94–55.31	SNV, SVM	0.91	2.93	[443]
	mg/g		7.34–29.26	SNV, ACO-PLS	0.91	1.04	[471]
	Catechins, mg/g		48.33–190.02	SNV, SVM	0.98	9.83	[443]
	TPC, mg GAE/g		46.05–169.02	SNV, ACO-PLS	0.83	14.38	[471]
	Classification	Origin		SG-1st der., SPA-LDA	100%		[472]
	Categories			SNV, SVM	>90%		[473]
partially fermented	Total catechins, mg/g		3.95–138.37	S, 1st der, 2nd der., mPLS	0.90	13.52	[474]
	Theanine, mg/g		1.43–6.04	smoothing, 1st der., PLS, 2nd der., PLS	0.90	0.29	
Black, green, yellow oolong	Caffeine, mg/g		16.08–65.24	IVSO-PLSR	0.92	3.96	[475]
	Catechin, mg/g		32.28–198.21	SG + 1st der., IVSO-PLS	0.95	11.41	
	Theanine, mg/g		0.51–24.50	SGS, SNV, IVO-PLS	0.84	2.53	
Chinese tea	TPC g GAE/100 g DM		6.08–34.29	MSC + 1st der., SGS, CARS-PLS	0.99	0.595	[476]
dark, black, oolong, green							
	Caffeine, %		2.10–4.99	MSC + 1st der., SGS, CARS-PLS	0.99	0.07	
	Free amino acids, TE%		0.96–3.65	MSC + 1st der., PLS + SGS, CARS-PLS	0.99	0.063	
Fresh tea leaves	Caffeine, mg/g		12.871–25.965	SGS, CARS-SPA-MLR	0.89	0.9506	[477]
	EC, mg/g		9.815–17.515	MSC, SGS, CARS-SPA-MLR	0.92	0.4595	
			ca. 30–70	SNV, CARS-LS-SVM	1.00	0.41	[478]
	EGC, mg/g		ca. 40–140	SNV, CARS-LS-SVM	1.00	1.586	[478]
			11.996–33.365	MSC, SGS, CARS-SPA-MLR	0.94	1.5494	[477]
	EGCG, mg/g		28.79–69.533	SGS, CARS-SPA-MLR	0.92	2.6633	[477]
			ca. 75–300	none, CARS-LS-SVM	0.99	4.23	[478]
	ECG, mg/g		7.730–25.979	SGS, CARS-SPA-MLR	0.89	1.3881	[477]
			ca. 30–110	none, CARS-LS-SVM	0.99	1.799	[478]
Lusan-Yunwu powder	TPC		n.i.	biPLS	0.95	8.33	[479]
	Free amino acids		n.i.	siPLS	0.91	4.96	
	TPC/FAA		n.i.	siPLS	0.93	0.437	
Matcha	TPC,	mg/g	11.848–18.943	1st der., SPA-siPLS	0.97	0.4806	[480]
	%		2.10–3.76	SNV, RF-PLS	0.86	0.82	[481]
	Free amino acids, %		8.51–14.58	SNV, RF-PLS	0.96	0.14	
	Free amino acids, mg/g		3.035–4.785	SGS, GA-siPLS	0.98	0.0887	[480]
	Polyphenols: amino acids ratio		2.421–6.214	SNV, SPA-siPLS	0.99	0.1602	
Oolong	Theanine		1.4262–6.0383	S, DT, PLSR, SVMR, GPR varsel. RC, UVE, VIP, SR, FPA	0.88	0.3219	[482]
	Theanine		1.42–6.04	DT, FPA-GPR	0.88	0.3191	
	Classification of origin			SNV + 2nd der., PLS-DA	85%		[483]
Green, oolong	Identification of varieties			SNV, ANN	100.00%		[445]
Pu-erh	Theanine, mg/g		5.32–19.41	SNV, weighted PLS	0.85	1.317	[462]
	Polysaccharides, g glucose/100 g extract		0.065–0.33	SGS, SNV, weighted PLS	0.84	0.0192	
	Total flavonoid, rutin/100 g ext.		0.568–1.798	SGS, MSC, weighted PLS	0.84	0.1528	
	Antioxidant activity		0.25–0.73	SNV, weighted PLS	0.87	0.0652	
	TPC, g GAE/100 g		7.02–13.55	SGS, MSC, weighted PLS	0.83	0.4532	
Pu-erh ripen powder	Caffeine, mg/g		18.7–33.4	1st der.,DT, PLS	0.87	1.58	[484]
	Catechin, mg/g		0.036–0.799	N, PLS	0.84	0.091	
	CG, mg/g		0.006–0.829	SNV, MSC, PLS	0.85	0.082	
	Gallocatechin, mg/g		0.009–0.797	MSC, SGS, 1st der., PLS	0.91	0.074	
	GCG, mg/g		0.004–0.326	MC, PLS	0.79	0.097	
	EC, mg/g		0.029–0.808	MSC, PLS	0.86	0.093	
	ECG, mg/g		0.007–0.703	MC, DT, PLS	0.85	0.077	
	EGC, mg/g		0.018–1.51	MC, DT, PLS	0.84	0.16	
	EGCG, mg/g		0.006–1.14	N, DT, PLS	0.81	0.066	
	Bitterness		2.15–5.20	1st der., 2nd der., PLS	0.57	0.391	
	Astringency		2.125–5.125	MC, 1st der., PLS	0.76	0.252	
Yuezhou Longjing	Caffeine, %		2.435–4.291	MC, CARS-PLS	0.91	0.1401	[485]
			2.5–4.3	BOSS-SVM	0.96	0.11	[486]
	Total catechins, %		10.1–27.693	SNV, VCPA-IRIV-PLS	0.88	0.8823	[485]
			10.10–23.66	MSC, CARS-PLS	0.79	1.06	[486]
	Sensory score		ca. 65–95	SNV, VCPA-IRIV-PLS	0.91	2.5784	[485]
			72.55–92.92	BOSS-SVM	0.94	2.06	[486]
Tea leaf	Caffeine, %		1.42–5.94	SVM	0.65	0.07	[487]
Tea varieties	Classification			SNV, SIMCA	α-error 0.2		[488]
White, albino	Discrimination			SNV, DA	100%		[489]
Partially fermented	Classification	origin		1st der., SVM	>83%		[490]
	type and origin			1st der., SVM	100%		
Commercial	TPC, mg/kg		6.56–15.11	MSC, iSPA-PLS	0.93	0.599	[472]
	Classification			1st der., SVM	93%		[491]

#### 5.6.2. Coffee

The green coffee beans that we roast, grind, and brew to produce the popular beverage known all over the world are actually the seeds contained in fruits from trees and shrubs naturally grown in the shade of African forests, including the islands of Madagascar and Mauritius, and cultivated in tropical areas such as equatorial Africa, Java, Sumatra, and other islands of the Dutch East Indies, West Indies, India, Arabia, the islands of the Pacific, Mexico, and Central and South America [492].

Various species and cultivars of the coffee plant are cultivated, which fundamentally determine the chemical composition of green coffee. Additionally, different growing conditions, climatic factors, and the processing methods of green coffee also influence the quality of the final product, thus affecting its price. The assessment of coffee quality involves numerous aspects related to the coffee plant, green coffee, and the roasted coffee produced from it. Assessment of coffee quality is usually focused on factors that influence utilization of the final product, with consumer preferences being assessed in three primary ways: physical (e.g., bean size), sensorial (cup quality) and chemical analysis (key compounds attributed to quality). However, coffee quality results from interaction among many different factors, including genotype (G) and environment (E) [493]. Due to the high price of coffee, it is also worth investigating coffee adulteration, which can help prevent consumer deception and financial harm.

The potential of NIR spectroscopy to replace traditionally applied methods was examined in numerous cases, particularly in the classification and identification of various coffee types, as well as in relation to their physicochemical parameters and sensory properties. The models developed in connection with these different applications and their key characteristics are summarized in Table 20.

It is important to emphasize the moisture content in the case of green coffee, which must not exceed 12% to ensure microbiological stability. Several standards for reference, routine and rapid methods are already established for the determination of water content in green coffee [494].

Since the price of coffee can be significantly influenced by its geographical origin, NIR spectroscopy is often employed in combination with various chemometric methods to determine this factor. Primarily, scatter correction methods have been used for data pre-processing, while both linear (such as LDA, PLS-DA) and non-linear (such as ANN) multivariate statistical methods have been applied to develop classification models. In terms of chemical composition, the alkaloids of coffee, 5-caffeoylquinic acid (5-CQA), various sugars, and acidity have typically been analyzed. In addition to these, a new research direction has emerged, focusing on the elemental composition of coffee [495].

Green coffee becomes consumable through roasting, during which its chemical composition undergoes significant transformation. Pyrolysis and the Maillard reaction produce numerous compounds that are not characteristic of green coffee. NIR spectroscopy can be applied to monitor the roasting process, either by using spectral data alone or in combination with colour data or by monitoring the first and second cracks. Key quality attributes of roasted coffee include caffeine content, acidity, and sensory properties, which are typically determined using cupping tests. Among the latest research efforts, the analysis of aroma profiles determined by gas chromatography in combination with NIR spectroscopy gained attention. Coffee adulteration can be carried out by adding various ingredients such as chicory, corn, barley, or even sticks of the coffee plant. Additionally, Arabica coffee is often adulterated with Robusta, as the two species represent different price categories, although this price gap has diminished in recent times. The results of the research related to these analyses are summarized in Table 21.

**Table 20:** Overview of NIR Results for Green Coffee.

Sample	Investigated Parameter		Concentration Range	Chemometrics Data			Ref.
				Pre-Treatment, Regression	R^2^	Root Mean Square Error	
Green coffee	Caffeine, %		0.95–4.13	normalization + 1st der., PLS	0.86	0.4	[496]
			0.07–3.53	1st der., OPS-PLS	0.98	0.08	[497]
	Theobromine, %		0.10–0.67	normalization + 1st der., PLS	0.85	0.1	[496]
	Cafestol, mg/100 g		182.62–1392.28	SNV, mPLS	0.92	111.01	[498]
	Khaweol, mg/100 g		182.69–1265.41	SNV, mPLS	0.88	92.6	
	Acidity		6.75–9.0	SNV, PLS	0.83	0.21	[499]
			6.64–8.57	MSC, PLS	0.74	0.25	[500]
	Aftertaste		6.5–9.0	1st der., +SNV, PLS	0.8	0.22	[499]
			6.25–8.57	1st der., PLS	0.77	0.29	[500]
	Aroma		6.5–9.0	1st der., PLS	0.59	0.33	[499]
	Body		6.5–9.0	1st der., +MSC, PLS	0.78	0.22	[499]
			6.64–8.32	1st der., PLS	0.85	0.16	[500]
	Flavour		6.5–9.0	1st der., +SNV, PLS	0.66	0.29	[499]
			6.61–8.82	1st der., PLS	0.79	0.25	[500]
	Overall cup preference		6.5–9.0	1st der., +MSC, PLS	0.89	0.9	[499]
			6.57–8.68	1st der., PLS	0.73	0.29	[500]
	Preliminary cup quality		42–57	1st der., +SNV	0.67	1.72	[499]
			71–91	SLS, PLS	0.48	3.63	
	Total specialty cup quality		76.8–92.5	MSC, PLS	0.81	1.31	
			75.57–90.07	1st der., PLS	0.73	1.72	[500]
	Moisture content,	%	6–22	EMSC, PLS	0.9817	0.57	[501]
		g/kg	104.6–134.7	2nd der., PLS	0.81	2.946	[502]
	Electrical conductivity, us/cm/g		104.09–193.65	2nd der., PLS	0.94	7.94	[503]
	Potassium leaching, ppm		40.41–64.92	2nd der., PLS	0.8	3.22	
	Ph		5.70–5.84	1st der., PLS	0.781	0.022	
	Titratable acidity, ml NaOH n/100 g		108.46–150.65	SNV, PLS	0.921	3.752	[504]
	Balance		6.71–8.5	1st der., PLS	0.81	0.22	[500]
Green coffee	Fragrance		6.82–8.61	1st der., PLS	0.81	0.17	
	TPC, mg GAE/g		40.97–51.86	MSC, PLS	0.89	0.61	
	5-caffeoylquinic acid, %		0.75–4.69	1st der., OPS-PLS	0.96	0.27	[497]
	Trigonelline, %		0.14–1.62	1st der., OPS-PLS	0.96	0.07	
	Lipids, %		12.88–16.29	OSC, PLS	0.982	0.106	[505]
	Protein, %		13.06–15.98	OSC, PLS	0.991	0.053	
	Reducing sugar content, g/kg		0.10–2.60	SNV, PLS	0.781	0.236	[502]
	Soluble solids, g/kg		271.2–315.1	MSC, PLS	0.516	0.48	
	Total sugar content, g/kg		74.21–102.97	SNV, PLS	0.694	2.91	
Africa	d13C, ‰ vs. V-PDB		(−28.9573)–(−26.4017)	EMSC, PLS	0.88	0.28	[495]
	d18O, ‰ vs. V-SMOW		29.8348–32.2833	EMSC, PLS	0.92	0.32	
	d2H, ‰. vs. V-SMOW		(−50.2579)–(−34.7610)	EMSC, PLS	0.91	2.48	
	Lithium, ppm		0.011–0.0109	EMSC, PLS	0.88	0.0012	
	Sodium, ppm		10.0200–24.4300	EMSC, PLS	0.91	5.35	
	Manganese, ppm		11.5752–49.1093	EMS, PLS	0.89	5.30	
	Nickel, ppm		0.1504–0.4721	EMS, PLS	0.71	0.062	
	Selenium, ppm		0.0506–0.2050	EMS, PLS	0.62	0.024	
	Strontium, ppm		3.0243–6.4790	EMS, PLS	0.71	0.52	
	Molybdenum, ppm		0.0653–0.2221	EMS, PLS	0.7	0.018	
	Cadmium, ppm		0.0031–0.0068	EMS, PLS	0.91	0.00085	
	Barium, ppm		2.5606–5.9386	EMS, PLS	0.77	0.54	
	Lanthanum, ppm		0.0019–0.0473	EMS, PLS	0.88	0.0066	
South America	D13c, ‰. vs. V-PDB		(−29.4865)–(−25.9086)	EMS, PLS	0.93	0.37	[495]
	D18o, ‰. vs. V-SMOW		22.1487–29.6306	EMS, PLS	0.93	0.89	
	D2h, ‰. vs. V-SMOW		(−82.1523)–(−56.8713)	EMS, PLS	0.88	4.68	
	Lithium, ppm		0.0010–0.0080	EMS, PLS	0.7	0.0015	
	Boron, ppm		1.2369–20.8171	EMS, PLS	0.79	2.55	
	Nickel, ppm		0.0711–0.5460	EMS, PLS	0.73	0.088	
South America	Rubidium, ppm		3.4758–41.9333	EMS, PLS	0.69	5.39	
	Molybdenum, ppm		0.0529–0.5719	EMS, PLS	0.86	0.14	
	Caesium, ppm		0.0021–0.1844	EMS, PLS	0.74	0.038	
Classification	Natural, washed Arabica and Robusta			SNV, LDA	100%		[506]
	Origin			MSC, SVM	100%		[507]
				MSC, PLS-DA	98.00%		[508]
				PDS, SSOM	71%		[509]
				MSC	99.81%		[510]
				SNV + SGS, PCA-DA	57.60%	19.10%	[511]
	Species			EMSC, PLS-DA	90.50%	0.3641	[512]
	Continent			EMSC, RF	0.99		[513]
	Region			EMSC, RF	0.88		
	Country			EMSC, RF	0.88		
Discrimination	Civet coffee			FFBBANN	99.98%		[514]

**Table 21:** Overview of NIR Results for Roasted Coffee.

Sample	Investigated Parameter		Concentration Range	Chemometrics Data			Ref.
				Pre-Treatment, Regression	R^2^	Root Mean Square Error	
Roasted Coffee	Bitterness		1–5	SNV, C, IPW-PLS	0.9402	4.7364	[515]
			1–5	OPS, PLS	0.87	0.35	[516]
			1–10	MSC, BLC^,^ PLS	0.8351	0.0996	[517]
			1–5	2nd der., Jack-Knife PLS	0.835	0.2	[518]
	Mouthfeel		1–5	CC, IPW-PLS	0.8318	7.0117	[515]
	Aftertaste		1–5	CC, IPW-PLS	0.8676	6.5683	[515]
	Caffeine, mg/g		n.i.	MC, SELECT-OLS	0.998	0.0195	[519]
			12.037–15.115	2nd der., SCARS-PLS	0.918	0.375	[520]
	Colour (L, a*, b*)		n.i.	1st der., ISE-PLS	0.9732	1.624	[519]
			40–60 AU	1st der., iPLS	0.87	1.28	[521]
	Moisture content, %		ca. 0–26	VN, PLS	0.9773	0.39	[522]
			<1.28–>1.6	SNV, PLS	0.52	0.14	[523]
	Whole beans, %		0.79–4.04	SNV, 2nd der., PLS	0.95	0.15	[524]
	Ground coffee, %		1.03–4.97	SNV, 2nd der., PLS	0.97	0.13	
	Weight loss, g/dm^3^		ca. 0.5–1.2	SLS, PLS	0.9544	1.23	[522]
	Density, %		ca. 0–10	SLS, 1st der., PLS	0.9864	0.02	
	Perceived acidity		1–5	SNV, C, IPW-PLS	0.946	6.7675	[515]
	Acidity		1–5	OPS, PLS	0.84	0.28	[516]
			1–10	MSC, BLC, PLS	0.7986	0.1104	[517]
			0–4	2nd der., Jack-Knife-PLS	0.83	0.3	[518]
	Titratable acidity, ml NaOH/g		0.6–2.6	SNV, PLS	0.89	0.16	[525]
	Flavour		1–5	OPS, PLS	0.93	0.31	[516]
			1–10	MSC, BLC, PLS	0.7724	0.1313	[517]
	Residual flavour		1–10	MSC, BLC, PLS	0.7469	0.1545	[517]
	Cleanliness		1–5	OPS, PLS	0.91	0.38	[516]
	Body		1–5	OPS, PLS	0.88	0.27	[516]
			1–10	MSC, BLC, PLS	0.7988	0.2849	[517]
			1–5	2nd der., Jack-Knife-PLS	0.967	0.1	[518]
Roasted coffee	Overall quality		1–5	OPS, PLS	0.91	0.39	[516]
	5-caffeoylquinic acid,	%	1.7–10.3	SNV, PLS	0.76	1.1	[526]
		mg/mL	5–10	SPAs-PLS	0.795	0.695	[527]
	Mixture of defects, *w*/*w*		0–0.3	BLC, PLS	0.913	0.029	[528]
	Light sour, *w*/*w*		0–0.3	PLS	0.837	0.038	
	Dark sour, *w*/*w*		0–0.3	PLS	0.953	0.026	
	Black, *w*/*w*		0–0.3	PLS	0.918	0.028	
	Immature, *w*/*w*		0–0.3	BLC, PLS	0.903	0.029	
	Arabica/robusta ratio, %		20–100	SNV.1st der., 2nd der., BLC, iPLS	0.97	4.34	[521]
	Arabica/robusta ratio, %		0–100	2nd der., PLS	>0.9567	2.8–6.6	[529]
	Tapped density, g/L		<364–>396	SNV, PLS	0.7	13.7	[520]
	Powder granulometry, %		<18.9–>24.0	SNV, PLS	0.92	1.23	
	Astringency		1–10	MSC, BLC, PLS	0.8398	0.1339	[517]
	Power fragrance		1–10	MSC, BLC, PLS	0.7514	0.1493	
	Drink aroma		1–10	MSC, BLC, PLS	0.7533	0.1633	
	Overall quality		1–10	MSC, BLC, PLS	0.7357	0.1594	
	First crack start, Au		−0.0788–0.0730	MSC, PLS	0.95	0.0068	[530]
	First crack end, Au		−0.0895–0.0772	MSC, PLS	0.92	0.0091	
	Second crack start, Au		−0.0875–0.0818	MSC, PLS	0.99	0.0041	
	Second crack end, Au		−0.0094–0.0892	MSC, PLS	0.93	0.007	
	Roasting monitoring. Agtron scale whole bean		ca. 25–100	MSC, SGS, VIP-PLS	0.95	4.48	[531]
	Roasting monitoring. Agtron scale ground		ca. 20–120	MSC, SGS, VIP-PLS	0.98	3.67	
	Roasting degree			PLS-DA	>0.9		[532]
	Grading of specialty coffee		ca. 81–91	OSC, MC, PLS	0.98	0.52	[533]
	Intensity		4–13	2nd der., Jack-Knife-PLS	0.915	0.4	[518]
	Roast		1–5	2nd der., Jack-Knife-PLS	0.842	0.2	[518]
	HMF, mg/kg		148.11–435.15	MSC, RF	0.92	20.49	[534]
Roasted coffee	2-methyl-furan			PLS	0.92	0.34	[532]
	2.5-dimethyl-furan			PLS	0.94	0.28	
	2.3-pentadione			PLS	0.79	0.32	
	2.6-dimethyl 2.6-octadiene			PLS	0.81	0.48	
	1-methyl-1h-pyrrole			PLS	0.81	0.61	
	pyridine			PLS	0.91	0.31	
	2-pentyl-furan			PLS	0.81	0.33	
	tetrahydro-2-furancarbonyl chloride			PLS	0.77	0.9	
	2-furfurylthiol			PLS	0.93	0.52	
	2-[(methylthio)methyl]-furan			PLS	0.8	0.83	
	2.3-dimethyl-2-cyclopenten-1-one			PLS	0.8	0.62	
	propanoate 2-furanmethanol			PLS	0.92	0.25	
	2.2′-methylenebis-furan			PLS	0.85	0.45	
	4-hydroxy-butanoic acid			PLS	0.84	0.43	
	2-(2-furanylmethyl)-5-methyl-furan			PLS	0.86	0.53	
	5-methyl-2-furanmethanol			PLS	0.82	1.01	
	ethyl 2.3.6.7-tetrahydro-4-oxepinecarboxylate			PLS	0.88	0.45	
	3-methyl-2-butenoic acid			PLS	0.92	0.42	
	1-(2-furanylmethyl)-1h-pyrrole			PLS	0.84	0.38	
	2-methoxy-phenol			PLS	0.77	0.51	
	2.2′-[oxybis(methylene)]bis-furan			PLS	0.84	0.57	
	3-methyl-phenol			PLS	0.75	1.04	
	4-ethyl-2-methoxy-phenol			PLS	0.77	0.78	
	4-methyl-2(1h)-quinolinone			PLS	0.9	0.78	
	cyclopropyl carbinol			PLS	0.8	0.95	
Adulteration	arabica with robusta,		1–100%	1st der., OWAVEC, PLS	0.9996	0.79	[535]
	with corn		0–100%	2nd der., PLS	0.8589	11.4	[529]
			0–25%	SNV, auto scaling, PLS	1.00	0.64	[536]
	with peels/sticks		0–100%	2nd der., PLS	0.9788	4	[529]
	with chicory		0–25%	auto scaling, CNN	0.99	0.76	[536]
			2.5–27.5%	2nd der., LDA-MLR	0.997	1.54%	[537]
	with barley		0–25%	SNV, auto scaling, iPLS	1.00	0.60	[536]
	with robusta. %		2.5–27.5	2nd der., LDA-MLR	0.998	1.11%	[537]
Classification	Arabica/robusta			1st der., LDA	100%		[506]
				1st der., PLS-DA	100%/95%		[538]
	Cup profiles			PLS-DA	73–95%		[539]
	Origin			2nd der., SIMCA	100%		[540]
				RF	0.20/0.27/0.93	15.27/19.51/24.10	[534]
	Variety			2nd der., SIMCA	100%		[540]
	Roasting			SVM	0.86/0.59	38.64/15.31	[534]
Discrimination	F regular instant coffee			RMS, BO, iPLS-DA	100%	[541]	
	Wild and feeding coffee			Boruta filter-RF	100%	[542]	
Authentication	Gourmet coffee			OC, linear BLC, SNV, DD-SIMCA	100%	[543]	
	Decaffeinated instant coffee			RMS, OBC, DD-SIMCA	100%	[541]	
	Geographical origin. Whole bean			SNV, 2nd der., SVM	0.97		[544]
	Geographical origin. Ground bean			MSC, 2nd der., NN	0.96		[544]

#### 5.6.3. Chocolate

Cocoa (*Theobroma cacao* L.) and its products, such as chocolate, are widely consumed globally and are valued for their flavour and health benefits [545]. The cocoa or cacao tree originated in South and Central America but is now grown commercially in suitable environments between 20° north and 20° south. 

NIR spectroscopy can be an ideal tool for on-site applications, enabling continuous monitoring of cocoa beans during crucial stages such as post-harvest processing, fermentation, drying, and storage. By providing real-time data, producers can make informed decisions to optimize these processes, ultimately improving the flavour, texture, and shelf-life of the final product.

The application of NIR spectroscopy in cocoa beans extends to several critical areas, including the assessment of cocoa maturity, for which classification methods such as PLS-DA can be applied [546]. The quality of cocoa beans significantly influence the price of the raw material, and the sensory quality of the products made from them. In addition to the type of cocoa, the fermentation process also affects quality, which can be monitored by examining various parameters, such as the fermentation and fermentation index [545,547,548,549], the content of alkaloids [547,550], polyphenols [547,549,550,551], fats [549,550,552,553], acidity-influencing compounds [547,548,549,550,554], carbohydrates [547,553], and proteins [547,549,550,553]. The amount of cocoa shell in cocoa powder is also a quality parameter, which has been studied in both cocoa powder and cocoa products using PLS regression after applying various data pre-processing methods [550,555,556,557]. 

For proper drying and storage of cocoa, the determination of moisture content is also essential [548,550,552,553].

Cocoa beans are most commonly used to produce different types of chocolate. During production, it may be useful to analyze the cocoa mass, whose sucrose content was estimated by da Costa Filho et al. using PLS regression from samples collected during the production process [558]. NIR spectroscopy can also be applied to the analysis of final products (chocolate), where the nutritional information and cocoa content indicated on the packaging are of particular importance [559,560,561].

To build mathematical models, PLS regression was most frequently applied for both cocoa beans and other cocoa-based products, either alone or in combination with variable selection methods (Table 22).

**Table 22:** Overview of NIR Results for Cocoa bean and Chocolate.

Sample	Investigated Parameter	Concentration Range	Chemometrics Data			References
			Pre-Treatment, Regression	R^2^	Root Mean Square Error	
Cocoa bean	fermented/unfermented	n.i.	SG	0.86/0.84	n.i.	[545]
Pods	maturity	n.i.	none	86%	n.i.	[546]
Powder	detection of cocoa shell	0–10	1st der., PLS, TD	0.94	0.687	[557]
Fermented	sugar, %	26.16–39.49	PLS	0.88	0.21	[547]
	moisture, %	3.90–6.36	PLS	0.8	1.05	[547]
	fat, %	49.30–59.00	PLS	0.87	0.06	[547]
	N-value, %	1.95–2.46	PLS	0.88	0.14	[547]
	organic acid, %	0.32–2.04	PLS	0.67	0.1	[547]
	acetic acid, %	0.00–0.80	PLS	0.85	0.11	[547]
	lactic acid, %	0.26–1.37	PLS	0.82	0.32	[547]
	carbohydrates, %	0.49–4.00	PLS	0.82	0.25	[547]
	free amino acids, %	0.49–2.76	PLS	0.93	0.25	[547]
	phenols (HPLC), %	0.44–4.18	PLS	0.93	0.22	[547]
	epicatechin, %	0.04–3.69	PLS	0.88	0.75	[547]
	phenols, %	4.48–13.82	PLS	0.74	0.2	[547]
	methyl-xanthines, %	2.23–3.67	PLS	0.26	0.17	[547]
	caffeine, %	0.28–1.11	PLS	0.79	0.14	[547]
	theobromine, %	1.73–3.02	PLS	0.94	0.11	[547]
	pH-value	4.79–6.72	PLS	0.92	0.94	[547]
	fermentation time, day	0–10	PLS	n.i.	n.i.	[547]
Unfermented, dried	dry matter,%	90.9–97.3	SNV, DT, 2nd der., mPLS	0.98	0.16	[551]
	fat, %	46.1–64.2	SNV, DT, 2nd der., mPLS	0.94	0.89	[551]
	caffeine, %	0.05–0.94	SNV, DT, 2nd der., mPLS	0.94	0.05	[551]
	theobromine	0.49–1.68	SNV, DT, 2nd der., mPLS	0.88	0.08	[551]
	(-)-epicatechin, %	0.03–1.83	SNV, DT, 2nd der., mPLS	0.96	0.18	[551]
Whole	dry matter, %	93.30–95.76	mPLS	0.72	0.31	[549]
	protein, %	8.32–15.43	mPLS	0.66	0.06	[549]
	fat, %	36.96–48.39	mPLS	0.69	0.15	[549]
	ash, %	2.34–3.66	mPLS	0.51	0.02	[549]
	pH	4.84–6.47	mPLS	0.58	0.24	[549]
	titratable acidity, mmol NaOH/100 g	8.20–26.81	mPLS	0.46	2.98	[549]
	TPC, mg/g dry defatted powder	32.58–98.04	mPLS	0.03	0.67	[549]
	fermentation index	0.57–2.24	mPLS	0.07	0.03	[549]
Ground	fat, %	36.96–48.39	mPLS	0.76	0.13	[549]
	protein, %	8.32–15.43	mPLS	0.91	0.03	[549]
	TPC, mg/g dry defatted powder	32.58–98.04	mPLS	0.16	0.59	[549]
	pH-value	4.84–6.47	mPLS	0.88	0.13	[549]
	titratable acidity, mmol NaOH/100 g	8.20–26.81	mPLS	0.86	1.43	[549]
	fermentation index	0.57–2.24	mPLS	0.42	0.38	[549]
	dry matter, %	93.30–95.76	mPLS	0.9	0.18	[549]
	ash, %	2.34–3.66	mPLS	0.89	0.01	[549]
Diff. Varieties	moisture, %	5.64–29.13	MC, PLS	0.899	2.931	[548]
		6.0–10.3	1st der., PLS	0.68	0.42	[550]
		6.74–12.08	EMSC, PLS	0.92	0.37	[552]
		6.56–10.28	1st der., PLS	0.95	0.27	[553]
		6.56–10.28	1st der., PLS	0.96	0.26	[553]
	protein, %	13.8–16.0	MSC, PLS	0.75	0.25	[550]
		12.43–15.52	1st der., PLS	0.97	0.18	[553]
		12.43–15.52	SNV, PLS	0.81	0.46	[553]
	fat, %	35.26–45.75	EMSC, PLS	0.98	0.27	[552]
		41.0–48.7	1st der., PLS	0.67	1	[550]
		41.38–48.85	1st der., PLS	0.97	0.45	[553]
		41.38–48.85	1st der., PLS	0.95	0.67	[553]
	carbohydrates, %	26.65–31.45	MSC, 1st der., PLS	0.96	0.39	[553]
		26.65–31.45	MSC, 2nd der., PLS	0.91	0.57	[553]
	ash, %	3.25–4.13	MSC, 1st der., PLS	0.95	0.07	[553]
		3.25–4.13	MSC, 1st der., PLS	0.95	0.08	[553]
	pH	0.35–1.08	2nd der., PLS	0.815	0.171	[548]
		4.4–5.9	MSC, PLS	0.71	0.2	[550]
		4.45–6.78	SNV, PLS	0.824	0.251	[554]
	acidity, %	0.7–2.1	SNV, PLS	0.77	0.12	[550]
	total acidity, mEg NaOH/100 g	6.13–29.99	SNV, PLS	0.861	2.813	[554]
	shell, %	10.3–17.3	1st der., PLS	0.76	0.96	[550]
	total phenolic, %	3.0–7.9	MSC, PLS	0.89	0.43	[550]
	caffeine, %	0.04–0.26	MSC, PLS	0.79	0.02	[550]
	theobromine, %	0.8–1.5	MSC, PLS	0.77	0.06	[550]
	L*	44.00–47.68	n.i.	0.8	0.97	[553]
	a*	14.09–16.91	MSC, 1st der., PLS	0.73	0.54	[553]
	b*	7.81–15.35	2nd der., PLS	0.75	0.54	[553]
	fermentation index	4.78–5.88	2nd der., PLS	0.87	0.121	[548]
	discrimination of fermentation		MSC, PLS	100%		[548]
Cocoa products	cocoa shell content	0–10	MSC, PLS	0.72	1.7	[556]
Chocolate	physicochemical data		ANN	0.99	0.01	[562]
	discrimination of different types		MSC, SGS, MC, PLS-DA	80–100%		[560]
	cocoa nibs, %	30–90	SNV, PLS	0.998	0.7	[563]
Dark chocolate	theobromine, mg/g	4.41–11.90	SGS, PLS	0.801	0.78	[561]
	caffeine mg/g	0.55–1.20	SGS, PLS	0.825	0.09	[561]
Dark, milk chocolate	water, %	0.47–1.31	EMSC, PCR	0.998	4.7	[564]
	protein, %	7.44–10.43	EMSC, PCR	0.989	1.55	[564]
	fat, %	31.77–46.52	EMSC, PCR	0.992	0.57	[564]
	sugar, %	26.16–39.49	EMSC, PCR	0.998	0.85	[564]
Various chocolates (white, milk, dark, filled)	carbohydrates, %	43–64.9	RS, ANN	n.i.	1	[559]
	fat, %	24.5–44.0	RS, ANN	n.i.	1	[559]
	energy kJ/100 g	1678.0–2508.0	RS, ANN	n.i.	50	[559]
	cocoa content, %	6–75	RS, ANN	n.i.	1.4	[559]
Mass	sucrose	20–60	SNV, PLS	0.998	0.75	[558]

### 5.7. Honey

Honey is an extremely complex product, mainly composed of sugars and water, but various organic acids, proteins, minerals, vitamins, polyphenols, enzymes, etc., can also be found in it [565]. It is a natural sweetener that can be used in a highly versatile way. For the aforementioned reasons, it is often subjected to adulteration, such as the addition of foreign substances (e.g., sugar syrup), mislabelling, early extraction, or mixing high-quality honey with low-quality honey. The general quality requirements for honey are addressed by Directive 110/2001 [566]. However, due to the properties of honey, continuous development is needed in the area of quality assessment to detect adulteration [567,568]. 

NIR spectroscopy is applied to honey for various reasons, including quality control, botanical origin identification, geographical origin identification, and the detection of adulteration. Honey’s NIR results are summarized in Table 23.

#### 5.7.1. Botanical/Geographical Origin Identification 

The composition, colour, and taste of honey depend on the plant source. EU Regulation 110/2001 allows not only the geographical origin but also the floral source to be indicated on the honey label, provided its physical, chemical, sensory, and pollen composition match the characteristics of the specific floral source. In the case of polyfloral honeys, where the dominant pollen is less than 45%, and in some cases, such as acacia, less than 20%, the honeys do not show distinct physical and chemical characteristics. These honeys are highly variable in every aspect, making their identification/authentication more challenging [569]. 

Seven different botanically-sourced honeys were investigated. It was observed that each spectrum could visibly be associated with a specific type of honey. This was reflected in the intensity of absorbance. Distinct shape differences were noted between 4200 and 7100 cm^−1^. The greatest variation was caused by saccharides between 4200 and 5200 cm^−1^ [570]

Certain minerals, such as K, Mg, Ca, and P, can be found in honey. These elements depend on climate changes and botanical origin, with K being found in large quantities [571]. Escuredo et al. aimed to develop an NIR method for analyzing the pollen and mineral composition of honey collected from Northwest Spain, and then distinguish honeys of different botanical origins using multivariate statistical methods. The prediction was excellent for K (RPD = 5.2), Ca (RPD = 4.7), Mg (RPD = 4.7), and P (RPD = 4.0) [572].

In another study, the botanical origin of Galician, Mel de Galicia honey, protected by a geographical indication (PGI), was investigated using NIR spectroscopy. Pattern recognition techniques such as D-PLS, SIMCA, kNN, and MLF-NN were applied. The data were pre-processed with SNV. Among all the models, the best result was achieved with SIMCA, which provided a sensitivity of 93.3% [573]. Chinese honeys were also examined (Chen et al., 2012) according to their floral origin. NIRS was combined with BP-ANN and MD-DA classification methods. Based on their results, more accurate classification was achieved using the BP-ANN model [574]. 

Bodor et al. developed an LDA model to test NIRS for the identification of the botanical origin of honey. It was found that sunflower honey was the most successfully classified, alongside acacia, honeydew, and linden honey [575]. Woodcock et al. examined the potential of NIRS for determining geographical origin. Unfiltered samples from Ireland, Mexico, and Spain, and filtered samples from Ireland, Argentina, the Czech Republic, and Hungary were collected. It was found that SIMCA was the most effective classification model for unfiltered samples, while D-PLS provided better classification results for filtered samples [576].

Brazilian floral honeys were examined by Nunes et al., with their observations focusing on the carbohydrates and water content present in large quantities in the honey. Descriptive models created by calculating the principal components from the NIR spectrum dataset did not detect the sample groups based on geographical origin and harvest period. This was explained by the fact that Brazil is one of the most diverse regions in terms of plant species [577].

#### 5.7.2. Quality Control

When examining honey, challenges arise because the evaluation cannot rely on the analysis of a single parameter. For quality control, detection of adulteration and identification of botanical origin, is important to assess the physical-chemical properties, sensory attributes, and perform pollen analyses. However, these tests often require lengthy and complex sample preparation and measurements. Additionally, separate measurements must be applied for each chemical parameter determination. Previous studies did not investigate the effect of heat treatment used to dissolve crystals on NIR spectra [573,578,579,580]. One study conducted a two-factor experiment to examine the combined effect of honey phase and heat treatment on the moisture, colour, and NIR spectral data of honey. It was found that honey treated at 39 °C for 30 min did not show spectral sensitivity to heat treatment. Since long-term, high-temperature treatment (55 °C, 24 h) affects the colour, moisture, and HMF content of honey, it is recommended to use short-term and low-temperature preparation [581].

Previously, NIR spectroscopy was successfully applied in both transmission and transflection modes for the quantitative determination of individual parameters. Transmission provided better resolution and sharper peaks, and the performance of calibration using mPLS regression was found to be 30–70% better. It was determined that the shortest optical path length examined (1 mm) produced the least saturated spectrum in the range of 1300 to 2500 nm, resulting in the lowest standard error of cross-validation (SECV) for all analyzed components.

The methods were developed for the determination of moisture (SECV = 0.08, R² = 1.0), HMF (SECV = 0.60, R² = 0.88), glucose (SECV = 0.52, R² = 0.90), fructose (SECV = 0.57, R² = 0.94), sucrose (SECV = 0.28, R² = 0.91), maltose (SECV = 0.31, R² = 0.92), free acid (SECV = 3.51, R² = 0.75), and lactone (SECV = 0.44, R² = 0.42) content [582]. NIR and MIR methods were compared by Ruoff et al. regarding the sugar, moisture, acidity, proline, HMF content, and pH of honey. It was found that NIR showed better repeatability in many cases. The calibration models demonstrated good accuracy for determining water, glucose, fructose, sucrose, and total monosaccharides, as well as the fructose/glucose and glucose/water ratios. However, the prediction accuracy for smaller compounds, such as HMF and proline, free acidity, and other carbohydrates present in small amounts, as well as pH value and electrical conductivity, was low [578].

The HMF content is an important parameter in honey analysis, with the 110/2001 directive establishing a general limit of 40 mg/kg, and 80 mg/kg for tropical honeys. Several studies have explored the possibilities of using NIR spectroscopy for its detection. Good results were achieved using a PLS model in the 4252–4848 cm^−1^ region, and sufficient results were also achieved in the 4000–1000 cm^−1^ range [583,584]. The possibility of determining small components using NIR spectroscopy has also been investigated. Tahir et al. (2021) attempted to quantify volatile compounds (VCs) present in Sudanese honey samples. It was found that NIR was more effective for determining these compounds than FT-IR; however, promising results were obtained when the data were combined with CSA [585].

#### 5.7.3. Detection of Adulteration

The Codex Alimentarius and the EU Directive 110/2001 emphasize that no other food ingredients may be added to honey [566]. It is often mixed with cheaper materials for economic gain. Well-known adulterants include inverted syrups, which can be tailored to mimic the natural sucrose-glucose-fructose profile of honey and are generally difficult to detect. 

Various analytical techniques are applied to detect honey adulteration [568], such as stable carbon isotope ratio analysis [586], chromatographic techniques [587,588], spectroscopic techniques [589], and sensor-based techniques [590].

Although the usefulness of these methods for evaluating honey adulteration has been proven, they are time-consuming, destructive, and sometimes expensive. Therefore, fast, non-destructive, easy-to-use, and low-cost analytical methods need to be developed for detecting and quantifying honey adulteration. Most of the articles published on the topic of NIR and honey focus on methods developed to detect adulteration. The popularity of this research field also highlights the importance of the topic, as honey is the third most frequently adulterated food, after milk and olive oil [591].

Bázár et al. adulterated acacia honey samples with high-fructose corn syrup (HFCS) at levels ranging from 0 to 40%. The most accurate NIR prediction of the adulteration level was achieved using the full spectral range of 1300–1800 nm, which included absorption bands for both water and carbohydrates [580].

Chen et al. developed an NIR method to differentiate honey adulterated with HFCS. The best data processing was achieved through the DPLS regression equation using various pre-processing techniques, such as mean centring (MC) and the first derivative. The NIR spectra of unadulterated honey and honey samples adulterated with high-fructose corn syrup were recorded in the spectral range of 10,000–4000 cm^−1^. The aim was to use the DPLS method to distinguish between adulterated and unadulterated samples. During classification, 95% of the adulterated samples and 100% of the unadulterated samples were correctly classified [592].

Huang et al. examined 112 pure and 112 sugar syrup-adulterated samples. The aim was to develop a model by integrating NIR and ATR-FTIR spectral data to create a highly accurate and robust model for detecting honey adulteration. The best SVM model, optimized with specific parameters, demonstrated 100% accuracy, sensitivity, and specificity [593].

Rust et al. investigated the spectral data of honey, focusing on various factors such as storage temperature, adulteration, irradiation, and time. The data were evaluated using ANOVA-simultaneous component analysis (ASCA). Significant effects were observed in factors such as temperature, time, and adulteration on the spectra, while irradiation was not significant. A particularly strong interaction was observed between time and adulteration, with the largest deviation occurring immediately after fresh adulteration, which decreased within three months [594].

Zhu et al. used NIR spectroscopy with various chemometric methods to detect honey adulteration. PCA was performed for data compression, followed by wavelet transformation (WT). Five classification models were also tested: LS-SVM, SVM, BP-ANN, LDA, and KNN. It was found that WT proved to be better than PCA for data compression. The best classification model (95.1% accuracy) was achieved using LS-SVM [594].

The aim of the work by Benković et al. was to develop PLS and ANN models for the detection and quantification of acacia honey adulteration with glucose syrup. Their results showed that ANN modelling was more effective in predicting adulterated honey and its properties [595]. 

Jaggery is a common adulterant, especially in India, and contains sucrose, inverted sugar, moisture from honey, and insoluble substances from honey [596]. A method was developed by Kumaravelu and Gopal to detect honey adulteration with jaggery using the PLS model (SEC = 0.00751) (R² = 0.9924) [597].

Rust et al. (2021) applied ASCA (ANOVA-simultaneous component analysis) to examine and characterize the effects of storage temperature, the presence of sugar syrup adulterants, irradiation treatment, and ageing on the NIR spectra of honey samples over time [598].

**Table 23:** Overview of NIR Results for Honey.

Sample	Investigated Parameter	Concentration Range	Chemometrics Data			Ref.
			Regression	R^2^	Root Mean Square Error	
Botanical origin	*Tilia amurensis Rupr.*	n.i.	MSC, MD-DA	Classification: 100%		[574]
	*Robinia pseudoacacia* L.			Classification: 86.7%		
	*Vitex negundo yar. heterophylla Rehd*.			Classification: 40.0%		
	*Brassica campestris* L.			Classification: 100%		
	*Ziziphus jujuba Mill.* var. *inermis (Bunge) Rehd*			Classification: 86.7%		
	*Tilia amurensis Rupr.*		MSC, BP-ANN	Classification: 100%		
	*Robinia pseudoacacia* L.			Classification: 93.3%		
	*Vitex negundo yar. heterophylla Rehd*.			Classification: 80.0%		
	*Brassica campestris* L.			Classification: 100%		
	*Ziziphus jujuba Mill.* var. *inermis (Bunge) Rehd*			Classification: 73.3%		
	K, mg/100 g	37.7–294.9	MSC, 2nd der.	0.963	28.0; (RPD 5.2)	[572]
	Ca, mg/100 g	4.8–45.9	MSC, 2nd der.	0.956	2.8; (RPD 4.7)	
	Mg, mg/100 g	1.7–23.7	SNV, 2nd der.	0.955	2.3; (RPD 4.7)	
	P, mg/100 g	2.7–24.5	1st der.	0.939	1.3; (RPD 4.0)	
	Castanea, %	0.0 -87.9	1st der.	0.765	17.9; (RPD 2.1)	
	Eucalyptus, %	0.0–94.8	MSC, 2nd der.	0.837	21.1; (RPD 2.5)	
	Rubus, %	0.0- 73.1	DT, 1st der.	0.74	14.0; (RPD 2.0)	
	Erica, %	0.0–49.4	MSC, 2nd der.	0.965	2.5; (RPD 5.3)	
Quality control	Water, %	13.4–24.6	PLS	0.960	0.3	[578]
	Fructose, %	26.4–49.8		0.759	1.6	
	Glucose, %	18.5–40.0		0.814	1.6	
	Sucrose, %	0.0–6.7		0.629	0.6	
	Turanose, %	0.0–5.5		0.134	0.7	
	Nigerose, %	0.0–5.3		0.227	1.1	
	Maltose, %	0.0–4.9		0.197	0.9	
	Kojibiose, %	0.0–2.1		0.335	0.3	
	Trehalose, %	0.0–4.6		0.426	0.6	
	Isomaltose, %	0.0–3.4		0.313	0.5	
	Erlose, %	0.0–4.1		0.462	0.5	
	Melezitose, %	0.0–5.3		0.626	0.7	
	Raffinose, %	0.0–2.2		0.554	0.3	
	Gentiobiose, %	0.0–1.1		0.041	0.1	
	Melibiose, %	0.0–1.3		0.029	0.1	
	Maltotriose, %	0.0–1.9		0.009	0.2	
Quality control	Monosaccharides sum, %	44.9–78.2		0.743	2.5	
	Fructose/glucose ratio	0.89–2.11		0.833	0.08	
	Glucose/water ratio	1.09–2.60		0.814	0.12	
	Free acidity (meq/kg)	5–44		0.636	5	
	Hydroxymethylfurfural, mg/kg	0–112		0.435	2	
	Proline, mg/kg	158–1189		0.588	125	
	Electrical conductivity (mS/cm)	0.100–1.699		0.794	0.17	
	pH	3.5–6.1		0.622	0.3	
	HMF, mg/kg	10–231	PLS	0.98	7.44; (RPD 3.3)	[583]
	2-Furanmethanol, %	0.08–1.54	SG; SNV, PLS	0.764	0.29; 0.33	[599]
	Benzyl alcohol, %	0.17–2.59		0.836	0.38; 0.36	
	Phenyl ethyl alcohol, %	0.25–4.76		0.868	0.63; 0.66	
	Furfural, %	1.78–28.9		0.961	2.78; 2.45	
	Benzaldehyde, %	0.85–6.15		0.866	0.69; 0.43	
	5-Methyl furfural, %	0.29–2.40		0.801	0.33; 0.26	
	2-Heptanone, %	0.07–0.91		0.936	0.10; 0.10	
	Phenol, 2-methoxy, %	0.12–0.69		0.738	0.11; 0.10	
	4-Ketoisophorone, %	0.25–5.99		0.906	0.84; 0.53	
	Moisture, %	n.i.	S, 1st der., PLS	0.98	0.125	[600]
		13.40 ± 0.71	MSC, PLS	0.6623	0.7131	[595]
		13.40 ± 0.71	ANN, MLP	0.8503	0.6017	
	Soluble solids content, °Brix	n.i.	S, 1st der., PLS	0.99	0.127	[600]
		n.i.	SNV, PLS	0.98	1.79	[601]
	Conductivity (μS/cm)	17.83 ± 0.09	RS, PLS	0.7222	25.3602	[595]
	Total colour change	2.08(..)	MSC, PLS	0.2101	0.8631	
	TPC (mg GAE/kg of honey)	n.i.	RS, PLS	0.3308	19.8989	
	FRAP (µM Fe(II))	n.i.	RS, PLS	0.5015	7.7951	
	Total colour change	2.08(..)	ANN, MLP	0.9261	0.5244	
	Conductivity (μS/cm)	17.83 ± 0.09	ANN, MLP	0.8994	21.4561	
	TPC (mg GAE/kg of honey)	n.i.	ANN, MLP	0.5639	17.7901	
	FRAP (µM Fe(II))	n.i.	ANN, MLP	0.6726	8.2014	
Adulteration	jaggery, %	0–30	PCA, PLS	0.66	6.45	[596]
	Robinia honey	0–40	SNV, 2nd der., PCA, PLS	0.987	1.48	[580]
	with HFCS, %					
	with sugar syrup, %	10–60	2nd der., SVM, PCA	Sensitivity, Specificity, Accuracy 100%; 78.57%; 89.29%		[593]
	Amount of adulterant, %	0–100	MSC, PLS	0.8660	11.4736	[595]
	Amount of adulterant, %	0–100	ANN, MLP	0.9987	1.9674	
Origin	Protected geographical indication	-	SNV, SIMCA	Sensitivity: 93.3%; Specificity: 100%		[573]
	Irish honey	-	2nd der., SIMCA	Correct classification: 95.5%		[576]
	Mexican honey	-	SNV, SIMCA	Correct classification: 94.4%		
	Spanish honey	-	RS, SIMCA	Correct classification: 96.0%		

## 6. Conclusions

The independent use of near-infrared radiation for analytical purposes dates back to the 1980s. Improvements in device technology, the development of computers, and the introduction of data evaluation software have been key to the evolution of near-infrared techniques from a complementary method to an independent analytical technique. This review article aimed to summarize the NIR results published so far in the 21st century in the context of food testing. The early manuscripts from the 2000s mainly deal with work on the determination of macronutrients occurring in percentage quantities in raw materials and processed foods, such as dry matter, moisture, sugar, fat, and protein content. Newer chemometric programmes have made it possible to improve on previously developed models. A growing number of different variable selection methods have been used to establish more accurate correlations between the deformation and/or stretching vibrations of molecular groups and spectral regions. Consequently, several publications include a quantitative analysis of the components present in milligrammes per kilogramme (ppm). The development of chemometric techniques is, in fact, helping to narrow the range of observable concentrations, but it must be emphasized that their accuracy in modelling is in much doubt. The development of analytical tools and data processing has made it possible to use NIR not only for quantitative estimation but also for recognizing different samples and groups. These classification procedures are often based solely on spectral data and does not require reference measurements. 

The application of pattern recognition techniques can be quite extensive. They are used for origin determination, which is crucial for foods and raw materials where quality depends on origins, such as determining the botanical origin of honey, the animal origin of various dairy products or meats, or even the geographical origin of coffee or tea. A remarkable application of these models could be the qualitative prediction of pesticide residues, certain damages, or potential microbial contaminations. However, the most widespread application of pattern recognition techniques is related to the detection of food fraud. Counterfeiters often mix low-quality materials or agricultural waste with high-quality materials to sell them at a significant profit. This type of fraud is common in the coffee and tea industry, where the ground nature of the products masks the adulteration, or in the case of honey, where the higher quality honey is diluted with the less valuable one. In the case of meat products, these rapid, non-invasive methods are also capable of identifying ground meat with dubious composition, soy, and unwanted bone or connective tissue in processed meats, while for dairy products, they can detect diluted milk, which is often sold as natural.

In summary, NIR spectroscopy is an unavoidable technique in the analytical toolbox, combining with modern chemometric methods, it becomes one of the most promising analytical procedures in the food industry. The online application and the development of various portable, handheld devices make it increasingly suitable for rapidly monitoring of manufacturing technology processes and inter-process products, as well. 

## Figures and Tables

**Table 1:** Characteristic qualifying parameters of PLS regression.

Parameters	Calibration	Validation	Aim
	Notation		
Square of the determination coefficient	R^2^	Q^2^	The value of Q^2^ is pertinent for the correlation rating, ideally as close to 1 as possible.
Mean squared error	RMSEC	RMSECV; RMSEP	The goal is to attain the smallest value possible.
PLS principal component	3–10	3–10	The value is highly dependent on the number of samples. Generally, for approximately 100 samples, a cut-off range of 3–10 is advised. Below 3, the function tends to be underfitted, while above 10, it tends to be overfitted.
RPD— Ratio of Performance to Deviation	(1 − R^2^)^−0.5^	(1 − Q^2^)^−0.5^	If greater than 3, the function is appropriate for quantitative assessment. The calculated value is not independent of Q^2^.
bias		<0.1·RMSECV; <0.1·RMSEP	The goal is to be at least an order of magnitude smaller than the average validation error.

**Table 3:** NIR test results for meat.

Sample	Investigated Parameter		Concentration Range	Chemometrics Data			References
				Pre-Treatment, Regression	R^2^	Root Mean Square Error	
Beef—fresh	Dry matter, %		21.5–26.8	1st der., SNV, DT, PLS	0.77	0.58	[123]
			25.15 ± 1.22	1st der., 2nd der, MPLS	0.92	0.26	[79]
	Moisture, %		59.6–72.9	MLR	Grinder diameter (4 mm 8 mm) 0.75/0.81		[67]
			40.53–80.72	SNV, DT, PLS	0.72	2.18	[72]
	Lipid, %		6.2–21.7	MLR.	Grinder diameter (4 mm 8 mm) 0.73/0.88		[67]
			1.99 ± 1.20	1st der., 2nd der, MPLS	0.99	0.20	[79]
			0.08–14.11	1st derivative, SNV, DT, PLS	0.93	1.00	[61]
			0.43–46.03	SNV, DT, PLS	0.93	1.25	[72]
	Ash, %		0.93–1.2	SNV, DT, PLS	0.66	0.03	[123]
			1.00 ± 0.06	1st der., 2nd der, MPLS	0.86	0.09	[79]
	Protein, %		18.1–20.7	MLR	Grinder diameter (4 mm 8 mm) 0.23/0.27		[67]
			18.3–22.6	2nd der., PLS	0.82	0.48	[123]
			10.36–23.84	1st der., PLS	0.89	0.99	[72]
			22.16 ± 0.47	1st der., 2nd der, MPLS	0.99	0.20	[79]
	Collagen, mg/100 g		0.31–1.9	2nd der., PLS	0.18	0.30	[123]
			18.43 ± 5.30	1st der., 2nd der, MPLS	0.74	8.52	[79]
	Fatty acids			1st derivative, SNV, DT, PLS			[61]
	Total Fatty acids, %		0.58–16.3		0.90	1.16	
	C16:0		101–4051		0.86	355	
	C18:0		89–3086		0.93	202	
	C18:1 n-9		123–5339		0.90	402	
	C18:2 n-6		62.0–502		0.70	57.0	
	C18:3 n-3		6.35–128		0.51	19.8	
	C20:4 n-6		11.9–115		0.49	14.6	
	C20:5 n-3		0.00–39.0		0.10	7.61	
	C22:5 n-3		0.00–86.8		0.11	15.1	
	C22:6 n-3s		0.00–11.3		0.16	2.03	
Beef—fresh	Total SFAs		216–8116	1st derivative, SNV, DT, PLS	0.90	14.2	[61]
	Total MUFAs		185–7019		0.90	45.6	
	Total cis-MUFAs		163–6526		0.90	560	
	Total trans-MUFAs		8.4–545.6		0.78	514	
	Total CLAs		1.9–114		0.67	490	
	Total n-3 PUFAs		10.4–264		0.28	52.8	
	Total n-3 LC PUFAs		0.00–149		0.06	24.9	
	Total PUFA		148–955		0.71	105	
	Individual Fatty acids, mg/100 g			1st derivative, SNV, DT, PLS	0.86	355	
	Total CLAs, mg/100 g		1.9–114	2nd der., MPLS	0.67	14.2	
	Total PUFAs, mg/100 g		148–955	2nd der., MPLS	0.71	105	
Beef—freeze-dried	Dry matter, %		25.15 ± 1.22	1st der., 2nd der, MPLS	0.96	0.35	[79]
	Lipid, %		1.99 ± 1.20	1st der., 2nd der, MPLS	0.99	0.13	
	IMF, %		0.88–8.48	SNV, DT, 1st der. PLS	0.94	0.39	[124]
	Ash, %		1.00 ± 0.06	1st der., 2nd der, MPLS	0.44	0.03	[79]
	Protein, %		22.16 ± 0.47	1st der., 2nd der, MPLS	0.85	0.33	
	Collagen, mg/100 g		18.43 ± 5.30	1st der., 2nd der, MPLS	0.56	3.05	
	Tenderness		2.0–7.2	SNV, DT, PLSM	0.981	0.353	[66]
	Myoglobin (mg/g of muscle)		2.55–5.08	RS, PLSM	0.914	0.260	
	WHC (%of liquid expelled)		21.17–29.17	RS, PLSM	0.892	1.338	
	Total CLAs, mg/100 g		1.9–114	2nd der., MPLS	0.76	11.3	[61]
	Total PUFAs, mg/100 g		148–955	2nd der., MPLS	0.78	84.9	
	Hydroxyproline, %		0.1–3.3	SNV, PLSR	0.89	0.25	[125]
	L*		23.85–50.77	SNV, DT, PLSR	0.765	2.51	[69]
	a*		4.63–27.02	SNV, DT, PLSR	0.878	2.51	
	b*		3.27–21.14	SGS, 1st der., SNV, PLSR	0.767	1.44	
	Hue		21.74–58.06	SGS, 1st der., SNV, PLSR	0.924	4.06	
	Chroma		6.19–32.43″	SGS, 1st der., SNV, PLSR	0.867	2.43	
	Fatty acids, %			1st derivative, SNV, DT, PLS			[61]
	Total Fatty acid		0.58–16.3		0.88	319	
	C16:0		101–4051		0.92	212	
	C18:0		89–3086		0.91	370	
	C18:1 n-9		123–5339		0.67	58.6	
	C18:2 n-6		62.0–502		0.67	16.4	
Beef—freeze-dried	C18:3 n-3		6.35–128		0.55	14.0	[61]
	C20:4 n-6		11.9–115		0.17	6.55	
	C20:5 n-3		0.00–39.0		0.32	13.1	
	C22:5 n-3		0.00–86.8		0.22	1.74	
	C22:6 n-3		0.00–11.3		0.90	570	
	Total SFAs		216–8116		0.90	473	
	Total MUFAs		185–7019		0.90	457	
	Total cis-MUFAs		163–6526		0.79	50.4	
	Total trans-MUFAs		8.4–545.6		0.76	11.3	
	Total CLAs		1.9–114		0.47	37.9	
	Total n-3 PUFAs		10.4–264		0.25	24.8	
	Total n-3 LC PUFAs		0.00–149		0.78	84.9	
	Total PUFA		148–955		0.88	319	
	Adulteration with turkey meat		0–10%	SNV, PLS	Classification: 80.3%		[126]
			15–20%		Classification: 85%		
			30–40%		Classification: 90%		
			50%		Classification: 100%		
			100%		Classification: 100%		
	Ether extract, %		0.47–6.10	2nd der., PLS	0.82	0.44	[123]
	Adulteration, %		0–35 0–35				[127]
	with pork			RS, DA, PLS	0.9580	7.27 accuracy: 100%	
	with pork and duck			MSC, SGS, DA, PLS	0.9569	9.27; accuracy: 9.27	
	with chicken		0–100%	1st der., PLS	0.99	3.5	[128]
	with chicken and pork				0.93	4.7	
Ox	Protein, g/kg DM		588.7–851.0	MSC, 2nd der., PLS	0.874	20.33	[74]
	Myoglobin, g/kg DM		17.7–37.0	MSC, 2nd der., PLS	0.440	3.45	
	Collagen, g/kg DM		5.7–21.3	2nd der., PLS	0.472	3.82	
	Ether extract, g/kg DM		92.2–359.8	MSC, 2nd der., PLS	0.924	16.22	
	Gross energy, MJ/kg DM		24.0–28.7	MSC, 2nd der., PLS	0.941	0.29	
	Dry matter, g/kg FM		271.0–339.1	RS, PLS	0.874	6.75	[74]
	Ash, g/kg FM		31.7–57.7	RS, PLS	0.168	5.15	
Hamburger meat	Iron, mg/100 g		0.43–2.54	MC, 1st der., PLS	0.73	0.34	[129]
	Calcium, mg/100 g		5.69–36.99	MC, MSC, 1st der., PLS	0.72	22.59	
	Potassium, mg/100 g		208.48–391.15	MC, MSC, 1st der., PLS	0.93	68.01	
	Sodium, mg/100 g		49.44–978.65	MC, MSC, 1st der., PLS	0.96	2.78	
Pork	Fat, %		2.58–3.15	MSC, 1st der., PLSR	0.767	0.087	[94]
	Protein, %		19.15–23.01	MSC, 1st der., PLSR	0.757	0.405	[94]
			22.2 ± 0.7	1st and 2nd der., PLS	0.57	0.49	[130]
	Water, %		65.32–73.62	MSC, 1st der., PLSR	0.794	0.776	[94]
			73.7 ± 1.5	1st and 2nd der., PLS	0.71	0.94	[130]
	pH		5.06–5.98	MSC, 1st der., PLSR	0.824	0.104	[94]
	pH ultimate		5.12–6.27	MSC, 1st der., PLS	0.70; 0.75	0.11; 0.11	[76]
	Shear force, N		11.17–28.89	MSC, 1st der., PLSR	0.278	0.360	[94]
	IMF, %		0.51–2.75	2nd der., MLR	0.35	0.36	[75]
			0.1–4.3	2nd der., MPLS	0.70–0.86	0.26–0.36	[78]
	Intact		32.4–51.1	PCA, 1st and 2nd der. SGS, PLS	0.33	4.0	[112]
	IMF, g/kg						
	Moisture, %						
	Homogenized		694.3–713.0	PCA, 1st and 2nd der. SGS, PLS	0.66	3.1	
	IMF, g/kg						
	Moisture, %						
	IMF (g/kg)		0.22–7.12	n.i.	0.22; 0.33	1.09; 1.03	[76]
	IMF, %		3.2 ± 1.8	1st and 2nd der., PLS	0.84	0.73	[130]
	L*		38.6–63.35	PLS	0.84; 0.77	1.80; 2.02	[76]
	a*		(-) 1.78–4.67	MSC, PLS	0.75; 0.84	0.61; 0.61	
	b*		6.59–15.82	MSC, PLS	0.74; 0.81	1.14; 1.07	
	WBSF, N		25.87–62.03	MSC, 1st der., PLS	0.30; 0.25	4.98; 5.51	
	Tenderness			PLS	Accuracy 72%		
	Juiciness			PLS	Accuracy 73%		
	Fatty acids, %			Normalization, 1st der., PLS			[83]
	SFA		34.5–45.9		0.98	0.36	
	MUFA		40.5–53.6		0.88	0.77	
	PUFA		7.0–20.9		0.96	0.54	
	16:0		20.3–26.2		0.88	0.39	
	18:0		10.7–17.6		0.94	0.32	
Pork	18:1		37.1–49.1		0.92	0.59	[83]
	18:2n-6		5.8–17.7		0.86	0.84	
	18:3n-3		0.01–4.02		0.76	0.33	
	LC-PUFA		0.78–2		0.88	0.09	
	TFA		0.3–2.3		0.83	0.12	
	in vivo						[131]
	C16:0		17.8–25.5	SNV, DT, 1st der., PLS	0.74	1.24	
	C18:0		6.9–12.5	SNV, DT, 1st der., PLS	0.72	0.67	
	C18:1		46.7–59.1	SNV, DT, 1st der., PLS	0.77	1.42	
	C18:2		6.5–10.2	SNV, DT, 2nd der., PLS	0.60	0.36	
	carcass						
	C16:0		17.8–25.5	SNV, DT, 1st der., PLS	0.87	0.82	
	C18:0		6.9–12.5	SNV, DT, 1st der., PLS	0.46	0.94	
	C18:1		46.7–59.1	SNV, DT, 1st der., PLS	0.80	1.48	
	C18:2		6.5–10.2	SNV, DT, 2nd der., PLS	0.31	0.55	
	Minced			MSC, 2nd der., PLS			[132]
	L*		35.90–53.58		0.75	1.03	
	Myoglobin, mg/g		1.04–2.64		0.74	0.11	
	Hardness, N		2.68–20.31		0.74	0.99	
	Cohesiveness		0.17–0.39		0.79	0.02	
	Springiness, mm		0.52–2.15		0.79	0.08	
	Chewiness, N × mm		1.20–8.83		0.78	0.50	
	Intact						
	L*		35.90–53.58	MSC, 1st der., PLS	0.68	1.36	
	Myoglobin, mg/g		1.04–2.64	MSC, 2nd der., PLS	0.67	0.18	
	Hardness, N		2.68–20.31	MSC, 2nd der., PLS	0.80	1.00	
	Cohesiveness		0.17–0.39	MSC, 2nd der., PLS	0.61	0.03	
	Springiness, mm		0.52–2.15	MSC, 2nd der., PLS	0.60	0.17	
	Chewiness, N × mm		1.20–8.83	MSC, 2nd der., PLS	0.69	0.97	
	TBARS (malondialdehyde/kg)		0.16–0.68	MSC, HSI-PLS	0.932	0.0305	[91]
Lamb	Moisture, %		72.0–78.6	SNV, DT, MSC, PCA, 2nd der., PLS	0.67	0.69	[96]
	Fatty acid, mg/100 g	C14:0	10.2–154.84	1st and 2nd der., GA-PLS	0.70	11.98	[88]
	C16:0		170.52–1055		0.70	87.01	
	C16:1		7.8–56.7		0.63	5.43	
	C17:0		9.7–56.9″		0.60	4.69	
	C17:1		4.4–23.1		0.55	2.32	
	C18:0		173.6–761.2		0.53	73.09	
	C18:1 c9		269.4–1503.4		0.69	128.31	
	C18:1 c11		8.42–30.7		0.73	2.01	
	C18:2 n-6		45.2–107.9		0.62	5.88	
	C18:2 c9 t11		5.70–81.0		0.68	7.10	
	C18:1 t11		20.5–197.09		0.61	21.10	
	C18:3 n-3		27.91–79.13		0.53	6.11	
	C20:4		14.39–30.92		0.40	2.30	
	C20:5		15.19–31.51		0.50	2.41	
	C22:5		16.23–26.89		0.47	1.57	
	C22:6		3.38–10.54		0.32	1.69	
	SFA		393.13–2065		0.60	192.21	
	MUFA		289.3–1678.5		0.60	168.72	
	PUFA		191–533.9		0.67	27.86	
	IMF, %		0.3–4.6	SNV, DT, MSC, PCA, 2nd der., PLS	0.84	0.41	[96]
			3.49–18.54	1st and 2nd der., GA-PLS	0.69	1.6	[88]
			1.2–6.79	MSC, PCA, PLS	0.79	0.38	[100]
	Protein, %		53.49–84.33	2nd der., PLS	1.00	0.92	[98]
	Fat, %		7.30–51.80	2nd der., PLS	1.00	0.43	
	Dry matter, %		90.55–95.92	2nd der., PLS	0.96	0.38	
	Ash, %		2.27–4.67	2nd der., PLS	0.97	0.15	
	K, mg/kg freeze-dried meat		8300–11,500	2nd der., PLS	0.86	600.00	
	P, mg/kg freeze-dried meat		5400–10,400	1st der., PLS	0.88	900.00	
	Na, mg/kg freeze-dried meat		960–1629	normalized, PLS	0.89	77.89	
	Mg, mg/kg freeze-dried meat		500–700	1st der., PLS	0.92	40.00	
	Fe, mg/kg freeze-dried meat		26.20–47.90	normalized, PLS	0.88	3.15	
	Zn, mg/kg freeze-dried meat		51.50–72.30	normalized, PLS	0.86	3.59	
Mutton	Rebound		-	2nd der., SPA, PLS	0.94	0.05	[62]
	Volatile basic nitrogen		-	MSC-UVE, PLS	0.74	1.81	
Rabbit	Fatty acid, %	C14:0	1.66–3.12	1st and 2nd der., MSC, MPLS	0.21	0.26	[133]
	C16:0		22.85–34.76		0.83	1.21	
	C16:1 cis n-7		0.91–6.83		0.77	0.64	
	C18:0		5.03–9.74		0.50	0.63	
	C18:1 n-9		18.52–30.18		0.84	1.26	
	C18:1 n-7		0.96–1.73		0.33	0.15	
	C18:2 n-6		14.99–41.19		0.91	2.08	
	C18:3 n-3		1.82–4.72		0.59	0.47	
	C20:1		0.19–0.53		0.08	0.07	
	C20:2 n-6		0.23–0.63		0.23	0.08	
	C20:3 n-6		0.15–0.47		0.54	0.04	
	C20:4 n-6		0.65–3.17		0.63	0.31	
	SFA		30.26–46.03		0.85	1.43	
	MUFA		20.81–37.21		0.83	1.81	
	PUFA		20.11–46.78		0.93	2.03	
	SFA		162–858	SNV, DT, 1st der., 2nd der., MPLS	0.96	32.2	[90]
	MUFA		92–778		0.98	24.2	
	PUFA		143–568		0.83	37.2	
	n-6 PUFA		110–493		0.87	27.8	
	n-3 PUFA		23.6–82.2		0.50	7.87	
	Protein, %		18.1–26.3	SNV, DT, 1st der., 2nd der., MPLS	0.77	0.41	
	IMF, %		0.75–3.25	SNV, DT, 1st der., 2nd der., MPLS	0.98	0.07	
Chicken	Dry matter, %		20.45–26.43	RS, PLS	0.72	0.69	[134]
	Moisture, %		73.57–79.55	RS, PLS	0.72	0.69	
	Protein, %		48.47–66.74	MSC, 2nd der., MPLS	0.86	2.012	[135]
			13.89–19.4	RS, PLS	0.73	0.65	[134]
	Fat, %		15.15–34.66	MSC, 2nd der., MPLS	0.93	1.723	[135]
	Ash, %		7.67–11.08	MSC, 2nd der., MPLS	0.71	0.795	
			1.68–3.08	RS, PLS	0.74	0.19	[134]
Chicken	L*		38.14–49.99	PLS	0.69	1.73	[97]
			47.3–66.4	1st der., MPLS	0.74	2.3	[136]
			46.08–63.91	RS, PLS	0.71	3.30	[71]
			58.28–74.59	RS, PLS	0.84	1.40	[134]
	pH		5.51–6.15	PLS	0.71	0.09	[97]
			5.64–6.33	RS, PLS	0.58	0.24	[71]
			6.35–6.7	RS, PLS	0.78	0.03	[134]
	pHu		5.3–6.4	2nd der., MPLS	0.36	0.2	[136]
	DFD			n.i.	Accuracy 77.78%		[71]
	Normal or PSE			n.i.	Accuracy 82.35% or 75.00%		
	a*		−3.29–0.04	PLS	0.88	0.29	[97]
			5.1–13.3	1st der., VN, MPLS	0.51	1.2	[136]
			0.6–1.21	RS, PLS	0.72	0.08	[134]
	b*		−4.86–16.33	PLS	0.93	1.16	[97]
			3.6–12.1	MPLS	0.55	1.3	[136]
			14–21.95	RS, PLS	0.77	1.00	[134]
	Ether extract, %		3.55–4.98	RS, PLS	0.83	0.18	
	Thawing loss, %		1.16–12.42	PLS	0.70	1.00	[97]
	Cooking loss, %		13.36–29.18	PLS	0.76	1.88	
	Shear force, N		8.14–29.06	PLS	0.41	3.18	
	Drip loss, %		0.7–7.0	1st der., MPLS	0.73	0.8	[136]
Hen	Protein, %		83.0–93.5	SNV-DT, 1st der., MPLS	0.91	0.74	[137]
	Lipid, %		1.9–11.8	DT, 1st der., MPLS	0.99	0.24	
	Dry matter, %		91.8–94.8	DT, 1st der., MPLS	0.96	0.19	
	Ash, %		4.0–7.5	SNV, DT, 1st der., MPLS	0.05	0.65	
Poultry	hydroxyproline, %		0.4–1.5	SNV, PLS	0.82	0.11	[125]
Yak	Classification	400–780 nm	Grazing or Feedlot Yaks	original, PLS-DA	0.870	0.521	[138]
				SNV, PLS-DA	0.967	0.347	
				1st der., SNV, PLS-DA	0.829	0.590	
				2nd der., SNV, PLS-DA	0.795	0.724	
	780–2500 nm			original, PLS-DA	0.844	0.738	
				SNV, PLS-DA	0.705	0.724	
				1st der., SNV, PLS-DA	0.975	0.478	
				2nd der., SNV, PLS-DA	0.958	0.429	
Yak	400–2500 nm			original, PLS-DA	0.861	0.548	[138]
				SNV, PLS-DA	0.893	0.465	
				1st der., SNV, PLS-DA	0.904	0.481	
				2nd der., SNV, PLS-DA	0.989	0.449	
Alpaca	Classification	Pork	0–50%	SGS, SNV, MC, PLS	0.90	6.34	[139]
	Chicken			SGS, 1st der., MC, PLS	0.87	6.69	
	Beef			SGS, 1st der., MC, PLS	0.88	5.11	
Ostrich (freeze dried)	Crude protein, %		85.45–93.93	2nd der., PLS	0.97	0.64	[140]
	Fat, %		1.41–8.33	2nd der., PLS	0.99	0.18	
	Dry matter, %		94.53–99.37	2nd der., PLS	0.85	0.75	
	Ash, %		4.31–5.50	normalization, PLS	0.71	0.23	
Meat-type classification	Horse vs. beef vs. chicken vs. mutton vs. turkey vs. Pork (meat pieces)			2nd der., SNV, PCA, SVM-c	Prediction Accuracy 38.1%		[64]
	Horse vs. beef vs. chicken vs. mutton vs. turkey vs. Pork (minced meat)				Prediction Accuracy 42.9%		
	Horse vs. beef (meat pieces)				Prediction Accuracy 62.5%		
	Horse vs. beef (minced meat)				Prediction Accuracy 100.0%		
	Horse vs. chicken (meat pieces)				Prediction Accuracy 87.5%		
	Horse vs. chicken (minced meat)				Prediction Accuracy 75.0%		
	Horse vs. mutton (meat pieces)				Prediction Accuracy 87.5%		
	Horse vs. mutton (minced meat)				Prediction Accuracy 87.5%		
	Horse vs. turkey (meat pieces)				Prediction Accuracy 100.0%		
	Horse vs. turkey (minced meat)				Prediction Accuracy 75.0%		
	Horse vs. pork (meat pieces)				Prediction Accuracy 75.0%		
	Horse vs. pork (minced meat)				Prediction Accuracy 75.0%		
Adulteration in Meat	Chicken		0–100%	2nd der., SNV, PCA, PLS	0.85	13.83; RPD: 3.05	
	Mutton				0.94	7.52; RPD: 5.68	
	Pork				0.88	11.95; RPD: 2.19	
	All adulterated		5–50%	2nd der., PLS 2nd der., PLS RS, PLS 2nd der., PLS SNV, PLS RS, PLS 2nd der., PLS, SNV, PLS RS, PLS	0.5348	0.1914	[141]
	Lamb-pork				0.9381	0.0706	
	Lamb-chicken				0.9693	0.0490	
	Lamb-duck				0.9218	0.0782	
	Beef-pork				0.9207	0.0791	
	Beef-chicken				0.9542	0.0599	
	Beef-duck				0.9016	0.0872	
	Pork-chicken				0.9119	0.0842	
	Pork-duck				0.8932	0.1018	

**Table 4:** NIR test results for meat products.

Sample	Investigated Parameter		Concentration Range	Chemometrics Data			References
				Pre-Treatment, Regression	R^2^	Root Mean Square Error	
Sausages	control samples vs. treated		dry fermented	2nd der., SNV, OPLS-DA	Classification rate: 100%		[117]
	0 vs. 0.5 vs. 1 vs. 2 vs. 3						
	0 kGy				Classification rate: 46.7%,		
	0.5 kGy				Classification rate: 41.7%		
	1 kGy				Classification rate: 100%		
	2 kGy				Classification rate: 91.7%		
	3 kGy				Classification rate: 100%		
	Intact, %			SNV, DT, MSC, MPLS			[142]
	Fat		15.3–43.2		0.98	1.47	
	Moisture		29.5–41.9		0.93	0.97	
	Protein		20.1–36.1		0.97	1.08	
	Homogenized, %						
	Fat		15.3–43.2		0.99	0.71	
	Moisture		29.5–41.9		0.98	0.41	
	Protein		21.1–36.1		0.97	0.95	
	Minced, %						[143]
	Fat		8–31.7	PCR	0.97	1.38	
	Moisture		50.2–68.4		0.98	1.01	
	Protein		13.6–20.5		0.93	0.83	
	Homogenized, %						
	Fat		8–31.7	MSC, SNV, DT, MPLS	0.99	0.94	
	Moisture		50.2–68.4		0.98	0.77	
	Protein		13.6–20.5		0.93	0.87	
	Cured pork sausage, cured beef Hydroxyproline, %		0.13–0.74	SNV-D, MSC, 1st der., MPLS	0.80	0.05	[115]
	On-contact probe						[116]
	Moisture, %		16.98–65.82	1st der., MSC, PLS	0.997	0.675	
	aw		0.765–0.982	1st der., VN, PLS	0.988	0.006	
	NaCl, %		1.13–3.80	1st der., VN, PLS	0.974	0.117	
	Remote probe						
	Moisture, %		16.98–65.82	1st der., MSC, PLS	0.998	0.622	
	aw		0.765–0.982	1st der., MSC, PLS	0.985	0.007	
	NaCl, %		1.13–3.80	1st der., MSC, PLS	0.974	0.116	
Sausages	Emulsion-type	Moisture, %	41.19–69.98	MSC, PLS	0.99	0.86	[119]
	Fat, %		9.08–45.39		0.99	1.27	
	Protein, %		10.30–18.30		0.99	0.36	
	Residual nitrite, ppm		0.00–74.32		0.92	12.02	
	Remote Q410/A	Moisture, %	16.77–66.14	min-max norm., PLS	0.990	1.56	[116]
	aw		0.754–0.982	VN, PLS	0.984	0.01	
	NaCl, %		1.07–3.86	SLS, PLS	0.910	0.22	
	On-contact IN 268-2						
	Moisture, %		16.77–66.14	1st der., VN, PLS	0.983	1.86	
	aw		0.754–0.982	1st der., VN, PLS	0.948	0.01	
	NaCl, %		1.07–3.86	1st der., SLS, PLS	0.804	0.33	
	Dry-cured						[144]
	C12:0		0.06–0.10	SNV, DT, 2nd der., MPLS	0.03	0.01	
	C14:0		1.22 1.78	SNV, DT, 1st der., MPLS	0.63	0.07	
	C16:0		22.83–28.00	SNV, DT, 1st der., MPLS	0.84	0.58	
	C16:1		2.25–3.71	SNV, DT, 2nd der., MPLS	0.41	0.26	
	C17:0		0.13–0.35	SNV, DT, 2nd der., MPLS	0.04	0.04	
	C17:1		0.15–0.33	SNV, DT, 1st der., MPLS	0.03	0.04	
	C18:0		10.57–14.83	SNV, DT, 2nd der., MPLS	0.78	0.55	
	C18:1		42.97–52.59	SNV, DT, 2nd der., MPLS	0.58	1.51	
	C18:2		4.54–10.34	SNV, DT, 2nd der., MPLS	0.56	0.86	
	C18:3		0.37–1.14	SNV, DT, 2nd der., MPLS	0.56	0.16	
	C20:0		0.16–0.28	SNV, DT, 2nd der., MPLS	0.02	0.02	
	C20:1		0.39–1.09	SNV, DT, 1st der., MPLS	0.07	0.17	
	SFA		35.65–44.79	SNV, DT, 2nd der., MPLS	0.86	0.98	
	MUFA		46.85–56.82	SNV, DT, 2nd der., MPLS	0.53	1.47	
	PUFA		4.92–11.23	SNV, DT, 2nd der., MPLS	0.61	0.88	
Ham	Remote	Moisture, %	19.92–66.11	normalization, PLS	0.929	3.51	[121]
	aw		0.823–0.929	RS, PLS	0.618	0.01	
	NaCl, %		0.67–14.02	VN, 1st der., PLS	0.910	1.13	
	On-contact						
	Moisture, %		19.92–66.11	normalization, PLS	0.899	4.17	
	aw		0.823–0.929	VN, PLS	0.451	0.02	
	NaCl, %		0.67–14.02	normalization, PLS	0.861	1.40	

## Data Availability

No new data were created or analyzed in this study. Data sharing is not applicable to this article.

## Abbreviations

$\sqrt{Ref\;}$	Square Root of Reflectance
1/Ref	Inverse Reflectance
1D-CNN	The One-Dimensional Convolutional Neural Network
1st der.	1st derivative
2nd der.	2nd derivative
AA	Amino Acid
ABTS	2,2′-Azinobis-(3-ethylbenzothiazoline-6-sulfonic acid
AC	Accuracy
AC	Antioxidant Capacity
ANN	Artificial Neural Network
ASCA	ANOVA–Simultaneous Component Analysis
ASR	Averagely Segmentation of Spectral Graph Area-to-Perimeter Ratio Characteristic
BC	Baseline Correction
biPLS	Backward Interval Partial Least Squares
BLC	Base Line Correction
CARS	Competitive Adaptive Reweighted Sampling
CBAM-CNN	Convolutional Block Attention ModuleConvolutional Neural Networks
CC	Column Centering
CG	Gallocatechin
CT	Cooked Texture
CV	Computer Vision
DA	Discriminant Analysis
DMVN	Diagonal Modified Confusion Entropy
DPPH	2,2-Diphenyl-1-picrylhydrazyl
DT	Detrend
DW	Dry Weight
ECG	Epicatechin Gallocatechin
EGC	Epigallocatechin
EGCG	Epigallocatechin Gallate
EMSC	Extended Multiplicative Scatter Correction
EN	Electronic Nose
EPO	External Parameter Orthogonalization
Exp(R)	Exponential Reflectance
F	Fresh
FA	fatty acid
F_int_	Average values of the forces measured after failure point, the Flesh shearing (g)
FiLDA	Fuzzy Feature Extraction Method, Called Improved Null Linear Discriminant Analysis
FM	Fresh Muscle
FRAP	Ferric Reducing Ability of Plasma
FrD	freeze dried
GA	Genetic Algorithm
GAEq	Gallic acid equivalent
GCG	Gallocatechin Gallate
GLSW	Generalized Least Square Weighting
GSA	Gravitational Search Algorithm
HIS	Hyperspectral Imaging
ICA	Independent Component Analysis
inLDA	Improved Null Linear Discriminant Analysis
IMF	Intramuscular Fat
iPLS	Interval Partial Least Squares Regression
IPW-PLS	Iterative Predictor Weighting
IRIV	Iteratively Retaining Informative Variables
ISE-PLS	Iterative Stepwise Elimination PLS
KM	Kubelka-Munk spectra
kNN	k-nearest Neighbour
KPLS	Kernel PLS
LARS	Least Angle Regression
LDBN	Linear Deep Belief Network
Ln(Ref)	Base 10 Logarithmic Scale of the Reflectance Data
LS-SVM	Least-Squares Support-Vector Machines
LVA	Latent Variables Analysis
LWR-PLS	Locally Weighted Regression PLS
MAD	Mean Absolute Deviation
MC	Mean Centering
MCR-ALS	Multivariate Curve Resolution-Alternating Least Squares
MC-UVE-SPA	Monte Carlo Uninformative Variable Elimination Combining Successive Projections Algorithm
MD-DA	Mahalanobis discriminant analysis
MEMS	Microelectromechanical System
MH	Mahalanobis Distance
MLP	Multilayer Perceptron
MLR	Multiple Linear Regression
MN	Mean Normalized
MPLS	Modified Partial Least Square
MSC	Multiplicative Scatter Correction
MSE	Mean Square Error
MUFA	Monounsaturated Fatty Acid
MWPLS	Moving Window Partial Least Squares Regression
n.d.	Not Detected
n.i.	No Information
n.p.	No Pre-processing
NB	Naïve bayes
NCL	Normalization by Closure
OC	Offset correction
OCC	One-Class Classifiers
OLS	Ordinary Least Squares
OLSR	Ordinary Least Squares Regression
OPS	Ordered Predictors Selection
ORAC	Oxygen Radical Absorbance Capacity—μMol Eq trolox/g
OSC	Orthogonal Signal Correction
OWAVEC	Combination of Wavelet Analysis and an Orthogonalization Algorithm
PCA	Principal Component Analysis
PCR	Principal Component Regression
P_e_	Penetrating Energy in the Flesh
PLS	Partial Least Squares
PLS2-CM	PLS Soft Multiclass Compliant Classification Method
PLS-DA	Partial Least Squares Discriminant Analysis
PLS-kNN	K Nearest Neighbours—PLS
PLSR	Partial Least Squares Regression
PR	Prediction Rate
PSP	Purple Sweet Potato
PUFA	Polyunsaturated Fatty Acid
RBF-NN	Radial Basis Function Neural Networks
RS	Range Scaling
RC	Regression Coefficient
Ref^2^	Square of Reflectance
RF	Random Forest
RMSECV	Root Mean Square Error of Cross Validation
RMSEP	Root Mean Square Error of Prediction
ROC	Receiver Operating Characteristic
RR	Recognition Rate
RS	Raw Spectra
RT	Raw Texture
S	Smoothing
SENS	Sensitivity
SFA	Saturated Fatty Acid
SGS	Savitzky–Golay Smoothing
siPLS	Synergy Interval PLS
siSVR	Synergy Interval Support Vector Regression
SLS	Straight Line Subtraction
SMLR	Stepwise Multiple Linear Regression
SNV	Standard Normal Variate
SNV, DT	Standard Normal Variate transformation combined with Detrend
SPA	Successive Prediction Algorithm
SPEC	Specificity
SRRC	Stepwise Regression Combined with the Regression Coefficient
SS	Stability Selection
SSC	Soluble Solid Content
SVD	Singular Value Decomposition
SVM	Support Vector Machines
SVMc	Support Vector Machine Classification
TA	Titratable Acidity
TAC	Total Anthocyanin Content
TAC	total antioxidant capacity
TBARS	degree of lipid oxidation
TCA	Transfer Component Analysis
TEAC	Trolox Equivalent Antioxidant Capacity—μMol Eq trolox/g
TPC	Total Phenolic Content
Tr	Trolox
UVE	Uninformative Variable Elimination
VIP PLS	Variable Importance PLS
VN	Vector Normalisation
WHC	Water Holding Capacity
W_p_	Mechanical Work Needed to Reach Failure Point (gmm)
WSP	White Sweet Potato
